# Academic Community Partnership in Acute Promyelocytic Leukemia and Early Mortality: The ECOG-ACRIN EA9131 Trial

**DOI:** 10.1001/jamaoncol.2024.7033

**Published:** 2025-02-27

**Authors:** Anand P. Jillella, Sandra J. Lee, Jessica K. Altman, Selina M. Luger, Martin S. Tallman, James M. Foran, Danielle Bradshaw, Lisa Y. Law, Locke J. Bryan, Abdallah Abou Zahr, Kebede H. Begna, Alexander E. Perl, Joseph J. L. Vadakara, Rubina Qamar, Raymond C. Bergan, Michael J. Fisch, Ruth C. Carlos, Lynne I. Wagner, Vamsi K. Kota, Mark R. Litzow

**Affiliations:** 1 Division of Hematology and Oncology, Georgia Cancer Center, Augusta University, Augusta, Georgia; 2 Dana-Farber Cancer Institute, Boston, Massachusetts; 3 Robert H. Lurie Comprehensive Cancer Center, Northwestern University Feinberg School of Medicine, Chicago, Illinois; 4 Abramson Cancer Center, University of Pennsylvania School of Medicine, Philadelphia; 5 Mayo Clinic Comprehensive Cancer Center, Jacksonville, Florida; 6 Roseville Medical Center, Roseville, California; 7 Sanford Roger Maris Cancer Center, Fargo, North Dakota; 8 Geisinger Medical Center, Danville, Pennsylvania; 9 Advocate Aurora Health Care, Milwaukee, Wisconsin; 10 Fred and Pamela Buffett Cancer Center, University of Nebraska, Omaha; 11 Department of General Oncology, The University of Texas MD Anderson Cancer Center, Houston; 12 Department of Radiology, University of Michigan, Ann Arbor; 13 Department of Health Policy and Management, University of North Carolina at Chapel Hill

## Abstract

**Importance:**

Acute promyelocytic leukemia (APL) is an acute illness that presents with cytopenia, infections, and disseminated intravascular coagulation. Achieving remission has been shown to make a major difference in patient outcomes; however, early death rates in the first month have been as high as 30% due to acute presentation, comorbidities, the rarity of APL, and clinician inexperience.

**Objective:**

To develop treatment strategies that would decrease estimated 1-month mortality from 30% to below 15%.

**Design, Setting, and Participants:**

In this nonrandomized clinical trial, a treatment algorithm that focused on supportive care was used to prevent early death in patients with APL treated at academic and community health centers between August 2017 and July 2021. Because of the rarity of the disease, expert support was available 24/7 from 7 designated APL experts at 6 participating academic lead centers, and included an additional 293 community centers. When a patient presented with APL, an expert was contacted and a consensus treatment plan was developed using the algorithm and expert suggestions. There were no exclusion criteria and all patients with a confirmed diagnosis of APL regardless of age or comorbid conditions were enrolled. Expert support was provided throughout induction. Initial data analysis was conducted May 2023.

**Main Outcomes and Measures:**

One-month mortality; additional objectives were to compare outcomes in academic and community centers and assess 1-year and overall survival.

**Results:**

A total of 201 patients were enrolled from 43 centers; 62 at lead centers and 139 from 37 community centers. The median age was 53 years (range, 18-91 years), with 72 patients (35.8%) who were aged 60 years or older; 105 patients (52.2%) were male. Fifty-two patients (26.4%) were diagnosed with high-risk APL. The 1-month mortality rate was 6 deaths of 201 patients (3.0%; 95% CI, 1.1%-6.4%) after adjusting for 1 interim analysis. In a secondary analysis using the Kaplan-Meier method, the 1-month overall survival (OS) rate was 97.0% (95% CI, 93.5%-98.6%) and the 1-year OS rate was 94.5% (95% CI, 90.3%-96.9%).

**Conclusions and Relevance:**

In this nonrandomized clinical trial, use of an algorithm combined with expert support resulted in a dramatic decrease in early death in academic and community centers. Population-wide survival improved in this highly curable disease, which suggests that implementing an accessible support system with APL experts for comanagement is the most logical next step.

**Trial Registration:**

ClinicalTrials.gov Identifier: NCT03253848

## Key Points

**Question:**

Does the use of a standardized algorithm and expert support in the treatment of acute promyelocytic leukemia academic and community centers decrease induction mortality?

**Findings:**

In this nonrandomized clinical trial including 201 patients, 1-month mortality was reduced from an estimated 30% to 3% using specialist support and an algorithmically developed treatment plan.

**Conclusion:**

The use of a standardized algorithm with expert support decreases early deaths in acute promyelocytic leukemia and increases population-wide survival.

## Teaser

This nonrandomized clinical trial examines the effectiveness of treatment for acute promyelocytic leukemia with a standardized algorithm with export support to reduce early deaths.

## Introduction

Large multicenter trials that resulted in the current standard of care for acute promyelocytic leukemia (APL) have used a combination of all-trans retinoic acid (ATRA) and arsenic trioxide (ATO), with and without anthracyclines and/or gemtuzumab ozogamicin. The cure rates in recent benchmark clinical trials have been 94% to 98%.^1,2,3^ Patients with APL present with cytopenias, a bleeding and clotting tendency due to disseminated intravascular coagulation (DIC), thrombocytopenia, and infection. However, bleeding, clotting, and differentiation syndrome (DS) that develop after initiating therapy make the induction period challenging. In clinical trials, early deaths during the first month occur in 2% to 6% of patients. Considering that patients in clinical trials are younger, highly selected with good organ function, and treated in experienced centers under strict protocol guidelines, early death is an underrepresentation of what occurs in standard practice in academic and community centers. Reports from single institutions, pooled data from multiple institutions, and population-based registries report early deaths rates (ie, during the first 30 days of therapy) of 17% to 37%.^4,5,6,7,8,9,10,11,12,13,14,15^ In addition, population-wide survival from national registries and US Surveillance, Epidemiology, and End Results Program (SEER) data suggest that 1-year survival is 65% to 70% and does not match survival in trials.^5,6,7,11^ Current trials that are changing the sequence of drugs, using oral drugs, and altering treatment duration will have little impact in improving population-wide survival. The only intervention to improve survival among all comers is to decrease induction mortality.

We recognized early deaths as a problem as early as 2008, and this was subsequently shown to be true by other groups.^4,5,6^ A critical analysis of the problem at our institution and a subsequent study revealed that most centers lacked a treatment algorithm.^16^ Because of the rarity of the disease, treating physician inexperience may be a contributing factor. In a pilot study conducted in Georgia and South Carolina to decrease early deaths, we developed a simplified treatment algorithm that succinctly defined treatment from diagnosis to completion of induction. In addition, 5 regional APL experts were identified that could be contacted to help manage induction both at academic and community centers. In a cohort of 120 patients, early deaths were decreased from an expected 30% to 8.5%.^17^ In the prospective, multicenter EA9131 trial (NCT03253848) a similar approach was implemented on a wider scale to decrease 1-month mortality from 30% to 15% or below. Additional objectives compared outcomes for academic and community centers and assessed overall survival (OS).

## Methods

Patients aged 18 years or older were included and required a confirmed diagnosis of APL. The confirmation required a positive test for t(15;17) using fluorescence in situ hybridization, cytogenetics, or a positive promyelocytic leukemia–retinoic acid receptor α by polymerase chain reaction. This trial was conducted through the ECOG-ACRIN National Cancer Institute Community Oncology Research Program (NCORP) Research Base (EA9131), and open to NCORP Community Sites and Minority or Underserved Sites, a geographically expansive network of community-based oncology practices that brings cancer clinical trials to patients in their own communities. The treating community oncologist had to contact an APL expert within 3 days after starting APL directed therapy. Comanagement and treatment could start as soon as the diagnosis was suspected and contact was made, but consent could be delayed until 7 days after initiating therapy. There were no exclusion criteria (including age or comorbid conditions) except refusal to undergo treatment and supportive care such as blood transfusions. Race and ethnicity data were self-reported (American Indian, Asian, Black, Native Hawaiian, White, and unknown or unreported, with Hispanic ethnicity categorized separately). The study was approved for all trial sites by an institutional review board central to the ECOG-ACRIN Cancer Research Group, and written informed consent was obtained from all patients. This study followed the Consolidated Standards of Reporting Trials (CONSORT) reporting guideline.

### Study Treatments and Supportive Care

Based on extensive review or prior research, the algorithm on supportive care and standard treatment options was modified by ECOG-ACRIN study investigators (see trial protocol in Supplement 1). The focus was expeditious workup, induction regimens, management of coagulopathy, and prevention and treatment of DS and infections. Additionally, management of complications induced by therapy such as leukocytosis, elevated liver function tests, and prolonged QT interval were addressed.

Adherence to standard APL therapy as recommended by established guidelines was encouraged. Suggested regimens were ATRA and/or ATO for non–high-risk patients, and ATRA and/or ATO with idarubicin and/or gemtuzumab ozogamicin for high-risk patients.^1,2,3^ The supportive care measures were per previously published guidelines.^18,19,20^ The recommended platelet count and fibrinogen levels were above 50 × 10^3^/μL (to convert to × 10^9 ^per liter, multiply by 1.0) and 150 mg/dL (to convert to grams per liter, multiply by 0.01), respectively. An oral prednisone dose of 0.5 mg/kg was recommended at diagnosis in non–high-risk patients and dexamethasone, 10 mg, administered intravenously twice a day in high-risk patients. Patients were weighed at admission on a bedside scale (not bed scale), and diuretics were used to maintain patients at admission weight.^21,22^ At the first sign of DS, interventions were increasing the dose of steroids, decreasing the dose of ATRA and ATO or holding them. Hyperleukocytosis was managed with hydroxyurea and other cytotoxic drugs. Increased liver function tests to 3 to 5 times the upper limit of normal were managed by holding ATRA or ATO or both and restarting after normalization.

Because there were no exclusion criteria, standard guidelines had to be modified for very old and frail patients with organ dysfunction. Given the wide age variation and the protean manifestations, doses and sequence of drugs had to be adjusted. We recommended that patients aged 60 years or older and/or with significant comorbidities receive dose-reduced ATRA at 25 mg/m^2^, with ATO added after 10 to 14 days provided there was no DS or leukocytosis.^17,23^ The algorithm was amended as we gained more experience and doses of ATRA and ATO were decreased further patients older than 70 years. Single agent ATRA at 10 mg/m^2^ was initiated and ATO added much later or given only during consolidation.

### APL Expert Support

**Figure 1.**
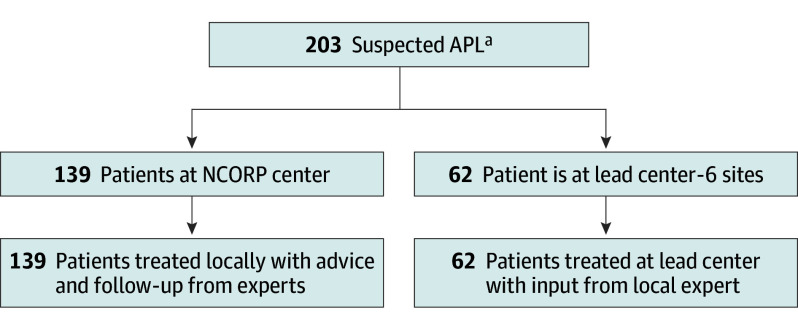
Trial Flowchart APL indicates acute promyelocytic leukemia; NCORP, National Cancer Institute Community Oncology Research Program. ^a^One patient was excluded from analysis due to a registration error and 1 patient withdrew consent. No patients were excluded on enrollment.

Seven APL experts at 6 lead centers were designated and available 24/7 to help manage the patients. The 6 lead centers were the Medical College of Georgia (Augusta, Georgia); Memorial Sloan Kettering Cancer Center (New York, New York); University of Pennsylvania (Philadelphia, Pennsylvania); Northwestern University (Chicago, Illinois); and 2 locations of the Mayo Clinic (Rochester, Minnesota; Jacksonville, Florida). At the 6 lead centers, patients were treated with input from the local expert. When a patient presented to an NCORP site, 1 of the 7 APL experts was contacted. The APL expert and the treating community oncologist developed a consensus treatment plan, but the patient was treated at the community site (Figure 1). An email of approximately 15 suggestions tailored to the patient was sent to the treating physician (eFigure in Supplement 2). There was ongoing communication by phone and/or email between the APL expert and the community oncologist throughout induction. For patients with more complicated APL cases, communication took place between all 7 APL experts and a consensus treatment plan was communicated to the treating site. A communication log documenting the correspondence between the APL expert and community oncologist was maintained and recorded in the ECOG-ACRIN database.

Because the end point of the study was to decrease early deaths, consolidation, maintenance, and follow up were per treating center discretion. Relapse and survival data were collected and follow up was for 1 year after the last patient was enrolled.

### Statistical Analysis

With 200 patients enrolled (180 eligible), the study was designed to have at least 90% power with a 2-sided type I error rate of <.05 for the hypothesis testing of reducing 1-month mortality rate from 30% to 15% or below. One interim analysis was planned at 50% information time using the O’Brien and Fleming boundary, and 95% repeated confidence intervals (RCI) were generated. One-month mortality rate was estimated by dividing the total number of deaths within 1 month of initial diagnosis by total number of cases with at least 1-month follow-up data. The OS data were also evaluated using the method of Kaplan-Meier. OS in the lead and community centers was compared using the log-rank test. Initial data analysis was conducted May 2023.

## Results

Between August 2017 and July 2021, 203 patients were registered in 43 centers. One patient was excluded due to a registration error; and a second patient withdrew consent. Of the 201 eligible patients, 62 were treated at the 6 lead centers and 139 at 37 NCORP community centers with collaboration from the lead centers. The Augusta University APL experts (A.P.J. and V.K.K.) were the first site of contact for the 109 of 139 patients (78.4%) treated at the NCORP community centers. The median age was 53 years (range, 18-91 years), with 72 patients (35.8%) who were aged 60 years or older; 105 patients (52.2%) were male; 11 patients identified as Asian (5.5%), 23 as Black (11.4%), and 158 as White (78.6%), with 36 (17.8%) reporting Hispanic ethnicity across all categories (Table 1). Fifty-two patients (26.4%) were high-risk, defined as having a white cell count of 10 000 or higher at diagnosis. During the COVID-19 pandemic, 19 of 52 patients reported high-risk disease (36.5%).

**Table 1.** Patient Characteristics

Characteristic	Patients, No. (%)		
	Lead center (n = 62)	Community center (n = 139)	All (N = 201)
Age, median (range), y	57 (18-87)	51 (19-91)	53 (18-91)
WBC count ≥10 000/μL	17 (27.4)	36 (25.9)	53 (26.4)
BMI			
No. of patients	62	138	200
Median (range)	33.1 (19.5-63.3)	30.8 (16.4-71.5)	31.3 (16.4-71.5)
Unknown or unreported	0	1	1
Sex			
Female	36 (58.1)	60 (43.2)	96 (47.8)
Male	26 (41.9)	79 (56.8)	105 (52.2)
Charlson Comorbidity Index score ≥3	21 (33.9)	34 (25.2)	55 (27.5)
Race			
American Indian	1 (1.6)	2 (1.5)	3 (1.5)
Asian	1 (1.6)	10 (7.2)	11 (5.5)
Black	10 (16.3)	13 (9.3)	23 (11.4)
Native Hawaiian	0	1 (0.5)	1 (0.5)
White	49 (79.1)	109 (78.4)	158 (78.6)
Unknown or unreported	1 (1.6)	4 (2.9)	5 (3.0)
Ethnicity			
Hispanic	3 (4.8)	33 (23.7)	36 (17.8)
Non-Hispanic	58 (93.6)	103 (74.1)	161 (80.1)
Unknown or missing	1 (1.6)	3 (2.2)	4 (2.0)

Abbreviation: BMI, body mass index (calculated as weight in kilograms divided by height in meters squared); WBC, white blood cell.

Body mass index (BMI; calculated as weight in kilograms divided by height in meters squared) evaluation noted that 118 of 200 patients (59.0%) were obese (BMI of 30 or higher), of which 57 (28.5%) were severely obese (BMI 35 or higher). Fifty-five patients (27.4%) had a Charlson Comorbidity Index score of 3 or more. Eighteen patients had a prior solid tumor that was treated. By treatment site, patient characteristics were median age 57 years (range, 18-87 years) at the lead centers vs 51 years (19-91 years) at the community centers; 27 of 62 patients (43.5%) at lead centers were aged 60 years or older vs 45 of 139 (32.4%) at community centers; 17 of 62 patients (27.4%) had high-risk disease at lead centers vs 36 of 139 (25.9%) at community centers; 21 of 62 patients (34.4%) at lead centers had a Charlson Comorbidity Index score of 3 or more vs 34 of 139 (24.4%) at community centers; and 42 of 62 (67.7%) were assessed as obese at lead centers vs 76 of 138 (55.1%) at community centers. Leukocytosis during treatment developed in 119 patients (59.2%)—cytotoxic drugs used were hydroxyurea, cytarabine, idarubicin, and gemtuzumab ozogamycin.

Of 182 patients assessed for relapse, 8 reported relapses, of whom 4 were alive at survival analysis. One of the surviving patients was noncompliant and received suboptimal consolidation. Four patients died of relapse; 1 was noncompliant and received suboptimal consolidation and 2 patients refused consolidation.

### Deaths

There were 6 deaths (3.0%) in month 1 (between days 2 and 27) and all deaths were due to DS (Table 2). Five of the 6 deaths were in older patients (ie, ages 70 to 81 years). There were 5 additional deaths in year 1: 1 patient died on day 35 from complications of APL induction (DS) and the other 4 were unrelated to APL treatment. There were 11 deaths after year 1, of which 4 were due to relapse. With a median follow-up time of 37.9 months (range, 12.1-64.2 months) as of April 27, 2023, there had been 22 deaths, 12 from lead centers and 10 from community centers (Table 2).

**Table 2.** Deaths During and After Month 1

Center	OS, d	Sex	Age, y	Race	COD
**Month 1 (6 deaths)**					
Lead center	1	Female	Early 70s	Unknown	DS
	5	Male	Early 70s	White	DS
	17	Male	Early 80s	White	DS
Community center	11	Male	Late 30s	White	DS
	16	Female	Mid 70s	White	DS
	24	Male	Late 70s	Black	DS
**Post 1 mo (16 deaths)**					
Lead center	53	Female	Mid 80s	Black	Discharged to hospice
	254	Female	Late 80s	White	Acute MI
	291	Female	Mid 70s	White	Ovarian cancer
	444	Male	Early 70s	White	APL relapse
	506	Female	Mid 70s	White	Breast cancer
	524	Male	Mid 70s	White	APL relapse
	567	Female	Mid 70s	White	Endometrial cancer
	960	Female	Early 60s	White	Ovarian cancer
	1019	Female	Mid 60s	White	APL relapse
Community center	36	Female	Early 90s	White	DS
	176	Female	Early 50s	White	COVID-19
	735	Female	Early 90s	White	Cerebrovascular incident
	739	Female	Mid 20s	White	APL relapse
	749	Male	Early 40s	Not reported	COVID-19
	1042	Female	Early 40s	White	APL relapse
	1339	Female	Early 70s	White	Anorectal cancer

Abbreviations: APL, acute promyelocytic leukemia; COD, cause of death; DS, differentiation syndrome; MI, myocardial infarction; OS, overall survival.

Per protocol, 1 interim analysis was conducted with data as of September 19, 2019, on 89 patients. One-month mortality rate was 2 of 89 patients (2.2%; 95% RCI, 0.01%-12.3%). As of April 27, 2023, all 201 patients had at least 1 month of follow-up data. The median follow-up time was 37.9 months (range, 12.1-64.2 months). The 1-month mortality rate was 6 of 201 patients (3.0%; 95% CI, 1.1%-6.4%), adjusting for 1 interim analysis. One-month mortality rate was 3 of 62 patients (4.8%; 95% CI, 1.0%-13.5%) in lead centers and 3 of 139 (2.2%; 95% CI, 0.4%-6.2%) in community centers.

**Figure 2.**
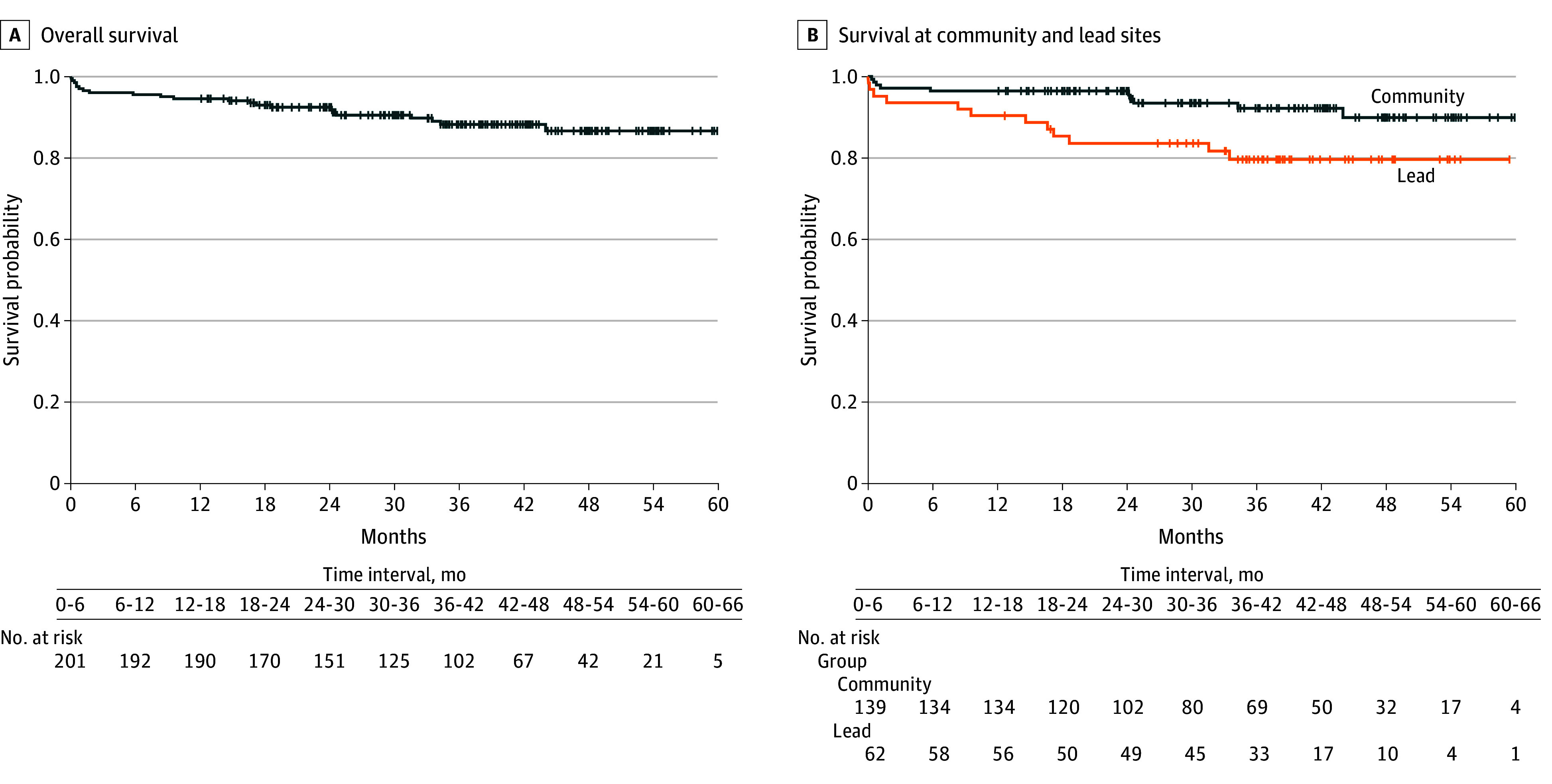
Kaplan-Meier Plot of Survival Probability of Study Patients

OS was measured from the first date of induction therapy to death. Patients who had not died were censored at the last known alive date. OS data were summarized using the Kaplan-Meier method based on all patients (Figure 2A). One-month OS rate was 97.0% (95% CI, 93.5%-98.6%) and 1-year OS rate was 94.5% (95% CI, 90.3%-96.9%). One-month OS rate was 95.2% (95% CI, 85.7%-98.4%) in lead centers and 97.8% (95% CI, 93.5%-99.3%) in community centers. One-year OS rate was 90.3% (95% CI, 79.7%-95.5%) in lead centers and 96.4% (95% CI, 91.6%-98.5%) in community centers. OS of the patients from community centers was significantly better (2-sided *P* = .02 by log-rank test) (Figure 2B).

## Discussion

Among a national sample of patients with APL, continuously available consultation from a team of APL experts resulted in a dramatic decrease in early deaths during the first month of treatment to 3%. The results support the value of a standardized algorithm and centralized expertise for the management of APL, a rare disease that renders patients vulnerable to early mortality. There is a high level of enthusiasm among oncologists to treat patients with APL given the exceptional cure rates, and thus treatment is offered to patients of all ages including those with health issues. When early deaths were initially identified, an observation we made was the lack of a written algorithm despite the complex nature of the disease. Subsequently, in a survey conducted of 23 APL-treating centers, only 3 hospitals (all academic) had a written algorithm and 2 hospitals followed the National Comprehensive Cancer Network (NCCN) guidelines.^16^ However, the NCCN supportive care guidelines are not detailed enough for effective management. Our algorithm was developed in 2009 and revised as additional experience was gained. Outside of clinical trials, supportive care measures are not closely followed. For instance, in the PETHEMA trial, most patients (87%) received furosemide for fluid retention.^24^ Review of our institutional experience prior to algorithm development showed that fluid status was not managed per guidelines.^21^ Review of the Swedish APL experience suggests poor adherence to supportive care recommendations.^5^

APL is an uncommon disease, with approximately 1500 cases diagnosed annually in the US.^25^ There are an estimated 17 500 medical oncologists, averaging 1 patient for every 10 oncologists per year.^26^ Because of the complex presentation and complications they experience, it is recommended that APL be treated in specialized centers.^5,19^ However, this is not practical given distance and social constraints. A pilot study conducted in Georgia and South Carolina showed that 62% of patients are treated in community centers and that 16 hospitals managed only 1 patient each over 3 years.^17^ Also, in the current EA9131 trial, 30 (69.8%) of community centers treated 5 patients or less in 4 years of the study. Over the study period, 3 of the higher accruing lead centers treated 20, 13, and 15 patients representing 3 to 5 patients per year. While the presumption is that academic centers treat more patients, data suggest that large centers manage only 5 to 10 patients per year.^7,8,10^ While some studies show that academic centers have a lower early death rate,^5,11^ there are reports that show the contrary.^8,9,10^ Based on these reports, it is possible that academic centers do somewhat better but could improve.

In our pilot trial, using a simple algorithm along with expert advice revealed no difference in early death between community (8.1%) and academic institutions (9.1%).^17^ Prior to the adoption of our model, early deaths in 2 academic centers that participated in the pilot trial was 37% and 17.5%.^9,10^ In the current trial, there were 3 of 62 (4.8%) deaths in academic centers and 3 of 139 (2.2%) in community centers, clearly showing improvement in academic centers as well.

Older patients and patients with medical problems are excluded from clinical trials. Based on 3 benchmark trials,^1,2,3^ 59 patients would have been excluded from the current trial. These patients have a significantly higher risk of early death than those eligible for clinical trials. In a review of patients ineligible for trials, the early death rate was 48%.^27^ Obesity is a risk factor for poor outcome and 59% of our patients had a BMI of 30 or higher.^28^ Fifty-five patients (27.4%) had a Charlson Comorbidity Index score of 3 or more.^29^ Population-based data show that age 60 years or older is a high-risk factor for death and is above 50%.^5,11,30^ While the median age of patients in trials is in the low to mid 40s,^1,2,3^ the median age in this study was 53 years, which is similar to the Swedish registry data (54 years) and representative of the general population. Seventy-two patients (35.8%) were older than 60 years; 31 patients were older than 70 years, including 9 who were older than 80 years. Taken together, patients in this trial were a much sicker and older cohort.

The superiority of regimens containing ATRA and/or ATO over chemotherapy treatments offers the chance of cure if early deaths can be reduced.^1,2,3^ From our previous experience, doses used in younger trial patients are poorly tolerated by older adults.^31^ A lower dose of ATRA at 25 mg/m^2^ was used in older patients and patients with compounding illnesses.^32^ In very old adults, ATRA dose was decreased further to 10 mg/m^2^. Arsenic trioxide was added later between days 10 to 14 with a 50% or greater dose reduction if there was no evidence of leukocytosis or DS. Among the 6 early deaths, 5 were aged older than 60 years, and the death rate in patients aged 60 years or older was 8.3%, which is considerably less than published population-based studies. The deaths were nonhemorrhagic and caused by DS between days 2 and 27. Although APL is associated with life-threatening bleeding, DS plays a more significant role in the elderly. In the Swedish and the Latin American IC-APL data, early deaths were nearly equally distributed between hemorrhagic and nonhemorrhagic causes.^5,33^ It is likely that the aggressive management of thrombocytopenia and hypofibrinogenemia, widely adapted in the US, has decreased hemorrhagic deaths. The treatment complications of DS might be more important in older adults and those with organ dysfunction, and standard doses of ATRA or ATO are not compatible in this group.

### Limitations

Our study had several limitations. Our algorithm by itself would not be expected to completely eliminate early death. For patients ineligible for clinical trials, it is not possible to develop an all-inclusive algorithm. Consulting with an expert is equally important and helps overcome this problem. The significance of centralized expertise in decreasing early death was shown by Rego et al^33^ in Latin America. Across 6 countries, patients younger than age 75 years treated using a standard protocol with weekly discussion by centralized experts decreased early death from 32% to 15%. One of the limitations of the trial is the lack of a control group. Based on previous experience, it was felt there would be unnecessary preventable deaths in the control group. Also, 1-year survival is from retrospective population and SEER data, and while it may not be complete it is the best available.

Multiple targeted therapies have been approved in this decade for various oncologic indications each with peculiar side effects. A decentralized approach in the application of these therapies might offer better care over a larger area and reduce disparities based on geographic location. A similar approach showed effective management of hepatitis C by primary care physicians in underserved areas in New Mexico under guidance from experts at the University of New Mexico.^34^ In our opinion, a similar approach to comanaging patients will be valuable in other oncologic conditions.

## Conclusion

This multicenter prospective nonrandomized clinical trial found that a simplified algorithm and partnership between experts and treating oncologists can significantly decrease early deaths in APL in academic and community centers. This model also paves the way for use in other conditions where education and academic-community partnerships could lead to better outcomes.

## Notes

Supplement 1.
Trial Protocol and Statistical Analysis Plan

Supplement 2. **eTable.** Resource Utilization Community Centers vs Lead Sites **eFigure.** Example Email Sent to Physicians

Supplement 3.
Data Sharing Statement
