# Supplementary material for: Academic Community Partnership in Acute Promyelocytic Leukemia and Early Mortality: The ECOG-ACRIN EA9131 Trial
Source: JAMA Oncol. 2025 Feb 27;11(4):400–7. doi: 10.1001/jamaoncol.2024.7033 (PMC11869096; doi:10.1001/jamaoncol.2024.7033)
Supplement: Supplement 1. — Trial Protocol and Statistical Analysis Plan [file jamaoncol-e247033-s001.pdf]

ECOG-ACRIN cancer research group
Reshaping the future of patient care

EA9131

A Simplified Patient Care Strategy to Decrease Early Deaths in Acute Promyelocytic Leukemia (APL)

STUDY CHAIR:	Anand P. Jillella, MD
STUDY CO-CHAIR:	Vamsi K. Kota, MD
STUDY STATISTICIAN:	Sandra Lee, ScD
PREVENTION COMMITTEE CHAIR:	Raymond Bergan, MD
CANCER CARE DELIVERY RESEARCH CHAIR:	Ruth Carlos, MD
PATIENT OUTCOMES & SURVIVORSHIPCHAIR:	Lynne I. Wagner, PhD
LEUKEMIA COMMITTEE CHAIR:	Mark R. Litzow, MD

Version Date: December 20, 2016

STUDY PARTICIPANTS	***ACTIVATION DATE***
**ALLIANCE** / Alliance for Clinical Trials in Oncology	***TBD***
**NRG** / NRG Oncology	
**SWOG** / SWOG	
**COG** / Children's Oncology Group	

STUDY CHAIR

Anand P. Jillella, MD
Winship Cancer Institute of Emory University
1365 Clifton Road NE, Room C1152
Atlanta, GA 30322
Office Phone: 404-778-1900
Cell Phone: 706-951-5144
Fax: 404-778-4755
Email: ajillel@emory.edu

STUDY CO-CHAIR

Vamsi K. Kota, MD
Winship Cancer Institute of Emory University
1365 Clifton Road NE, Room C1152
Atlanta, GA 30322
Office Phone: 404-778-1900
Cell Phone: 706-825-4091
Fax: 404-778-4755
Email: vkota@emory.edu

STUDY CHAIR LIAISON (SCL)

Prachi Karkhanis
Winship Cancer Institute of Emory University
1365 Clifton Road NE, Room C1152
Atlanta, GA 30322
Office Phone: 404-778-3663
Fax: 404-778-4755

Email: prachi.karkhanis@emory.edu

ECOG-ACRIN STUDY EXPERTS

Anand P. Jillella, MD
Winship Cancer Institute of Emory University
1365 Clifton Road NE, Room C1152
Atlanta, GA 30322
Office Phone: 404-778-1900
Cell Phone: 706-951-5144
Fax: 404-778-4755
Email: ajillel@emory.edu

Vamsi K. Kota, MD
Winship Cancer Institute of Emory University
1365 Clifton Road NE, Room C1152
Atlanta, GA 30322
Office Phone: 404-778-1900
Cell Phone: 706-825-4091
Fax: 404-778-4755
Email: vkota@emory.edu

Martin S. Tallman, MD
Memorial Sloan-Kettering Cancer Center
Leukemia Service, Department of Medicine
1275 York Avenue, New York, NY 10065
Office Phone: 212-639-3842
Cell Phone: 646-467-4670
Fax: 212-639-3841
Email: tallmanm@mskcc.org

Mark R. Litzow, MD
The Mayo Clinic
Division of Hematology
200 First Street, SW, Rochester, MN 55905
Office Phone: 507-266-0523
Cell Phone: 507-269-4848
Fax: 507-266-4972
Email: litzow.mark@mayo.edu

Jessica K. Altman, MD
Northwestern University Feinberg School of Medicine
Robert H. Lurie Comprehensive Cancer Center
303 E. Superior Street, Chicago, IL 60611
Office Phone: 312-503-1817
Cell Phone: 773-575-2598
Fax: 312-695-6189
Email: j-altman@northwestern.edu

Selina Luger, MD
Abramson Cancer Center of the University of Pennsylvania
2 West, Perelman Center for Advanced Medicine
3400 Civic Center Road, Philadelphia, PA, 19104
Office Phone: 215-662-6348
Cell Phone: 215-279-0657
Fax: 215-662-4064
Email: selina.luger@uphs.upenn.edu

James M. Foran, MD, FRCPC
Mayo Clinic Florida
Division of Hematology & Medical Oncology
4500 San Pablo Road, Jacksonville, FL 32224
Office Phone: 904-953-7292
Cell Phone: 904-955-3679
Fax: 904-953-2315
Email: foran.james@mayo.edu

Table of Contents

Schema 7
1. Introduction 8
  1.1 Rationale for Proposed Study 8
  1.2 Significance of the Study 10
  1.3 Study Overview 11
2. Objectives 13
  2.1 Primary Objective 13
  2.2 Secondary Objectives 13
3. Selection of Patients 14
  3.1 Eligibility Criteria 14
4. Registration Procedures 15
  4.1 Protocol Number 18
  4.2 Investigator Identification 18
  4.3 Patient Identification 18
  4.4 Eligibility Verification 18
  4.5 Additional Requirements 18
5. Methodology Plan 20
  5.1 Lead Sites 20
  5.2 Outlying Facility 21
  5.3 Study Accrual and Timeline 21
  5.4 Adverse Event Reporting Requirements 21
  5.5 Completion of Induction 23
  5.6 Consolidation 23
  5.7 Maintenance 23
  5.8 Duration of Follow-up 23
6. Study Parameters 24
7. Statistical Considerations 25
  7.1 Study Design and Objectives 25
  7.2 Sample Size Considerations and Monitoring Plan 25
  7.3 Statistical Analysis Plan 26
8. Electronic Data Capture 28
9. Patient Consent and Peer Judgment 28
10. References 28
Appendix I Acute Promyelocytic Leukemia Treatment Guidelines 29
Appendix II APL Expert and NCORP Provider Communication 32
Appendix III NCORP PROVIDER INFORMATION SHEET 33
Appendix IV Patient Thank You Letter 34

CANCER TRIALS SUPPORT UNIT (CTSU) ADDRESS AND CONTACT INFORMATION

**To submit site registration documents:**	**For patient enrollments:**	**Submit study data**
CTSU Regulatory Office<br>1818 Market Street, Suite 1100<br>Philadelphia, PA 19103<br>Phone – 1-866-651-CTSU<br>Fax – 215-569-0206<br>Email:<br>CTSURegulatory@ctsu.coccg.org<br>(for submitting regulatory documents only)	Please refer to the patient enrollment section of the protocol for instructions on using the Oncology Patient Enrollment Network (OPEN) which can be accessed at https://www.ctsu.org/OPEN_SYSTEM/ or https://OPEN.ctsu.org.<br>Contact the CTSU Help Desk with any OPEN-related questions at ctsucontact@westat.com.	Data collection for this study will be done exclusively through Medidata Rave.<br>Please see the data submission section of the protocol for further instructions.
The most current version of the **study protocol and all supporting documents** must be downloaded from the protocol-specific Web page of the CTSU Member Web site located at https://www.ctsu.org. Access to the CTSU members' website is managed through the Cancer Therapy and Evaluation Program - Identity and Access Management (CTEP-IAM) registration system and requires user log on with CTEP-IAM username and password.		
**For clinical questions (i.e., patient eligibility or treatment-related)** Contact the Study PI of the Coordinating Group.		
**For non-clinical questions (i.e., unrelated to patient eligibility, treatment, or data submission)** contact the CTSU Help Desk by phone or e-mail:<br>CTSU General Information Line – 1-888-823-5923, or ctsucontact@westat.com. All calls and correspondence will be triaged to the appropriate CTSU representative.		
**For detailed information on the regulatory and monitoring procedures for CTSU sites** please review the CTSU Regulatory and Monitoring Procedures policy located on the CTSU members' website https://www.ctsu.org > education and resources tab > CTSU Operations Information >CTSU Regulatory and Monitoring Policy		
**The CTSU Web site is located at** https://www.ctsu.org		

Schema

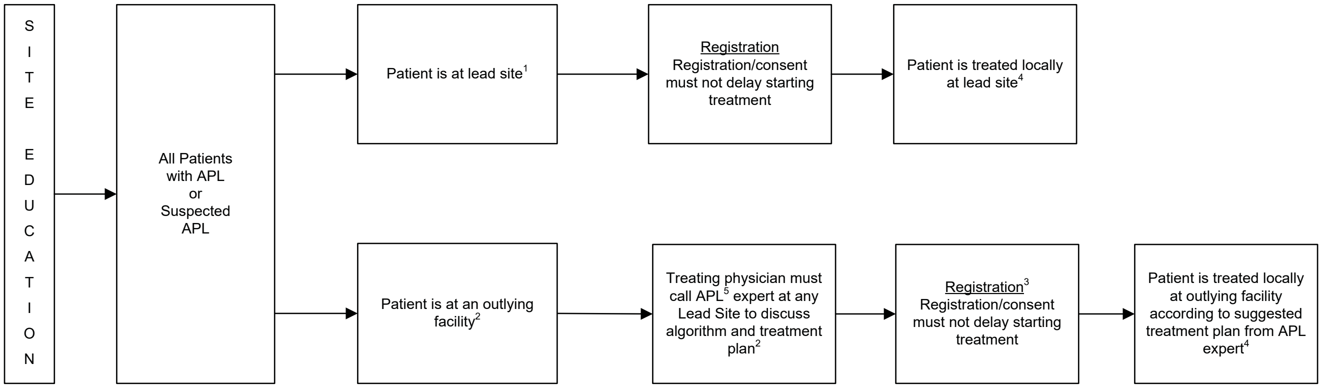

Total Accrual Goal= 200

1. Participation at lead sites will be limited to 30% of overall accrual. Lead sites are identified as: Emory, MSKCC, Mayo Clinic - Rochester, MN, Northwestern, University of Pennsylvania, Mayo Clinic - Jacksonville, FL.
2. It is preferred the APL expert for the catchment area is contacted first. It is suggested that the all be placed as soon as a diagnosis is suspected.
3. Registration is done only if treating physician calls expert within 5 days of starting treatment.
4. Follow treatment guidelines according to Appendix I. If necessary, deviations from guidelines may occur per discretion of treating physician and consulting physician.
5. 7 investigators at the lead sites have been identified as APL experts.

1. Introduction

1.1 Rationale for Proposed Study

Acute Promyelocytic Leukemia (APL) is a highly curable malignancy with most large cooperative group trials showing a survival above 90%. Patients entered in cooperative group trials are a highly selected group of patients and may not be a representation of outcomes in the general population. It is also likely that patients may not even be seen at a large treatment center due to the advanced nature of the disease and/or socioeconomic and distance constraints. However in recent years, data from single institutions as well as large population data bases show that approximately 1 in 3 patients diagnosed with APL die within the first month of diagnosis.[1-5] The first group to systematically analyze this is a population-based study from Sweden. All patients diagnosed with APL between 1997 and 2006 in the Swedish Adult Leukemia Registry were included in the study. A total of 105 patients with APL were diagnosed during that time and thirty (29%) of these patients died within 30 days of diagnosis with 23 of these 30 patients dying within the first week of diagnosis. The major cause of death was reported as bleeding, with differentiation syndrome, sepsis and multi-organ failure being other causes contributing to early mortality.[1]

While high early mortality was suspected in the US, definite evidence was established only recently by a well-designed epidemiologic study conducted by Park et al by utilizing the Surveillance, Epidemiology and End Results (SEER) program.[2] A total of 1,400 patients diagnosed with APL between 1992 and 2000 were identified. Data from 13 population based cancer registries that participate in the National Cancer Institute's SEER program were analyzed. The overall early death rate of all patients diagnosed with APL was 17.3% and 24% in patients aged > 55 years. The authors from the SEER study also concluded that this might be an underestimate due to the flaws in the SEER reporting system and the actual death rate may be higher than what the study showed. MD Anderson Cancer Center also reported their data from various time periods and compared them with SEER data.[3] From 2002-2008, the 5 year relative survival in APL patients was 64% which is contrary to the reported outcomes in clinical trials. This data also suggests that the lower survival observed in the general population may not be due to deaths in only smaller treatment centers but may also be a problem in larger metropolitan areas (Figure 1). Single institution experience from Stanford University showed an early death rate of 26%, suggesting that this problem might be pervasive even in larger treatment centers.[5]

Figure 1: Early Deaths in APL (Day 1 to 30)

Study	Total patients	Patients died	Mortality rate	Percentage of patients with hemorrhage in early death
**Brazil (2007)[4]**	134	43	32%	66%
**Swedish registry (2011)[1]**	105	30	29%	41%
**SEER data (2011)[2]**	1400	238	17% (24% in > 55yr)	Not discussed
**Stanford (2012)[5]**	70	19	26%	54%
**Ankara, Turkey, (2010)[8]**	49	20	40%	65%
**AIIMS, India (2011)[6]**	33	6	18.8%	Not discussed
**Germany > 60 years (2013)[9]**	91	24	26%	Not discussed
**Hiroshima, Japan > 65 years (2013)[7]**	32	7	21.3%	Not discussed
**GRU (our center) ASCO (2012)[10]**	19	7	37%	57%

Georgia Regents University Experience and Pilot Project:

At Georgia Regents University (GRU), Augusta, GA, from July 2005 to June 2009, 19 patients with APL were treated. Seven of these 19 (37%) patients died during induction due to the following reasons; however the 11 surviving patients are in molecular remission and are presumed cured with a median follow up of 2358 days.

1. Delay in diagnosis and transfer to a treating center
2. Bleeding from coagulopathy
3. Differentiation syndrome
4. Infections
5. Inconsistency in the treatment algorithm and physician inexperience.

After a thorough review, the investigators at GRU formulated a strategy to decrease early deaths and implemented it in 2010. This strategy aided physicians to rapidly diagnose the condition when suspected, and also follow a mechanism to rapidly transfer the patient from rural areas to GRU. A 1.5 page concise treatment algorithm to simplify the treatment process was developed and implemented at GRU. The algorithm was made available to physicians at outlying smaller treatment centers as well. In addition, investigators at GRU communicated with treating physicians in the community and offered guidance.

We treated or co-managed 143 patients thus far using this model, with 10 deaths (6.9%) Furthermore, after implementing the algorithm, data showed an encouraging decrease in induction mortality from an estimated 37% (N=19) to 6.9% (N=143) (Figure 2). Interestingly 40% of patients were treated in experienced leukemia centers and 60% were treated in outlying centers. Patients who survive the first 30 days have an excellent quality of life with a low chance of relapse and treatment related complications.

Figure 2: Survival Pre and Post Algorithm

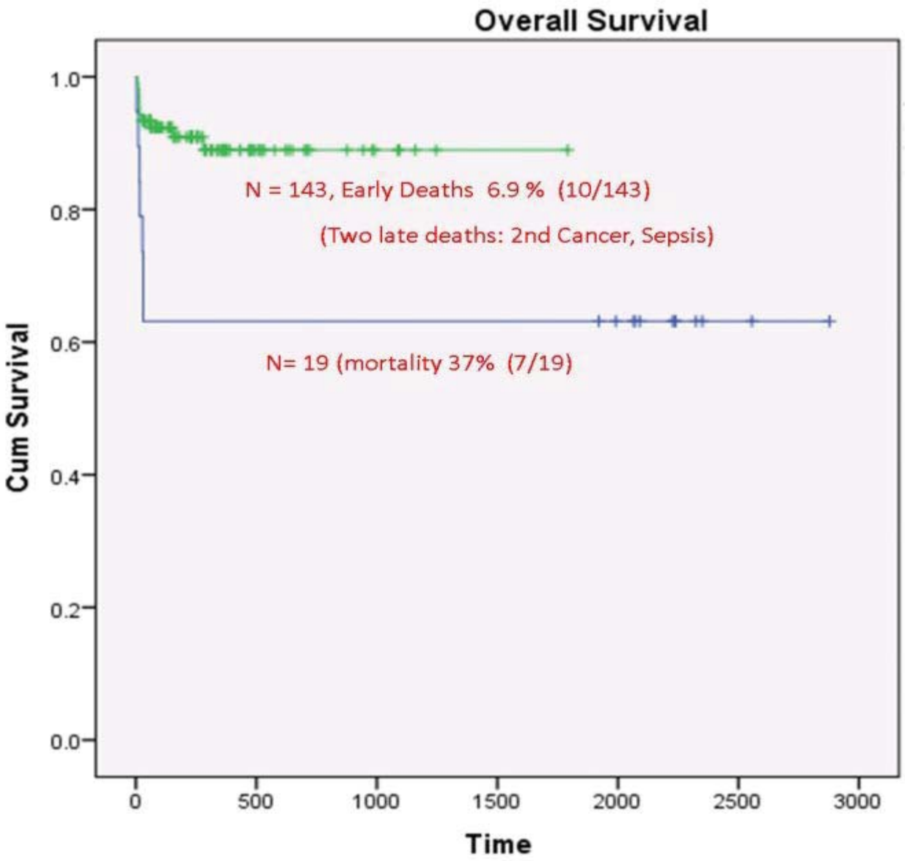

Ongoing Study:

Funding from the Leukemia & Lymphoma Society (LLS) supports the ongoing study efforts to follow the strategy developed at GRU as a model to implement it in the states of Georgia and South Carolina to cover a catchment population of 15 million; the goal is to reduce the induction mortality from an estimated 30% to under 15%.

1.2 Significance of the Study

As discussed earlier, the early mortality in APL continues to be high. The first large scale intervention to decrease early deaths in APL was undertaken by Rego et.al in Brazil, Mexico and Uruguay. In a report published in 2007, it was noted that in Brazil the mortality rate during induction in a cohort of 134 patients was 32%.[(4)] The cumulative mortality during induction and consolidation was 44.7%. The overall survival in this group was 52% which is significantly lower than what is expected in developed countries (62 to 86%). It was obvious that

decreasing induction mortality would make the most impact in improving overall survival in this patient population. Analysis of the causes of early death showed that delayed diagnosis, hemorrhage, differentiation syndrome and infection were major causes of death. This led to the development and implementation of a strategy that addressed all the above mentioned causes of early mortality. This effort was supported by the International Members Committee of the American Society of Hematology (ASH) in 2005. A simple and uniform protocol was developed and launched in Brazil, Mexico, Chile and Uruguay. A standard program for rapid diagnosis, early initiation of treatment, aggressive management of coagulopathy, expedited transfer to a larger center, management of differentiation syndrome and effective treatment of infection was implemented. From 2006 to 2010, 183 patients were enrolled; 122 in Brazil, 30 in Mexico, 23 in Chile and 8 in Uruguay. The cases were individually discussed by a core group of experts with the treating hematologists in outlying hospitals via Internet. One third of the patients were high risk and 52% of the patients were intermediate risk. The induction mortality was 15% a decrease from 32% prior to the implementation of this study. The major cause of early deaths was still hemorrhage (48% of deaths). In addition, the overall survival (OS) improved from 52% to 80% at 2 year follow-up.(11)

APL is an uncommon disease; however excellent treatment modalities are available with cure rates of 90%. Current trials that are changing the treatment schedules, adding new drugs, or withholding maintenance might only have a minimal impact on survival in the general population. The intervention that will increase population wide survival is decreasing early deaths. Treating oncologists, who are not routinely involved in the care of acute leukemia patients in smaller centers as well as in larger treatment centers, may not be familiar with the treatment or expected complications. Addressing these issues by educating these physicians and co-managing patients will help improve survival in a condition that has a very high chance of cure.

Patients that were managed using the simple algorithm (Appendix I) demonstrated remarkable improvement in the overall survival. More importantly, a network was established with other leukemia treatment centers in the catchment area and helped local oncologists manage patients and decreased the mortality to 6.9% in 143 patients managed thus far. By replicating these results across a much wider population under the ECOG/ACRIN umbrella with additional sites, it is expected that adherence to the treatment guidelines and consultation with APL experts will reduce mortality rates even more than what previous trials have shown.

If the study is positive, efforts will be made to implement the concept nationally and manage APL patients with use of the streamlined guidelines and expert support. A complement of experts is required to be made available permanently and the model should be followed for this project to be sustainable.

1.3 Study Overview

This is a multi-center study with 7 lead investigators at 6 lead sites: Emory University, Atlanta, GA; Memorial Sloan Kettering Cancer Center, New York, NY; Mayo Clinic, Rochester, MN; Northwestern University, Chicago, IL; University of Pennsylvania, Philadelphia, PA and Mayo Clinic, Jacksonville, FL. The study will be restricted to the above mentioned **SIX NCTN SITES** and will be referred to as lead sites. **Other Academic sites will not participate in this trial.**

Physicians treating patients at outlying centers will call one of the lead investigators as early as possible but no later than 5 days after APL directed treatment is started. The algorithm will be made available and the contacted lead investigator will provide guidance during induction and follow up treatment. The providing physicians will be encouraged to call as and when there are problems.

1.3.1 Treatment guidelines:

The treatment guidelines were developed after extensive review of the literature on standard treatment of APL. Physicians at the outlying centers will be provided this algorithm. The study does not exclude patients by age or comorbidities and complete adherence might not be feasible in certain clinical circumstances. This study is directed at co-managing patients by discussions between APL experts and treating physicians to improve the care.

1.3.2 Feasibility

The incidence of APL in the US is approximately 1,500 per year. Our goal is to target an accrual of 200 patients over a 4 year period. As this will be a data collection study, we expect to enroll these patients in this time frame. We do not plan to change the treatment from present standard of care and therefore will not include any new interventions. As described earlier, decreasing early mortality in this curable malignancy will have the biggest impact in improving outcomes. APL is a rare disease and physicians in some centers may not see enough APL patients to be proficient enough. Transferring these patients to larger academic centers is not always feasible for multiple reasons. As an increasing number of community hospitals are treating patients with acute leukemia, it is important to improve the survival in inexperienced centers as well. This would also make it easier for the patient and family and help them remain closer to home. From our experience in rural Georgia and South Carolina, most leukemia treatment centers have adequate supportive care infrastructure such as pharmacy, blood bank, pathology and sub-specialty services. We believe that expert guidance will ensure better adherence to the guidelines and earlier recognition of impending complications and thereby improve survival.

2. Objectives

2.1 Primary Objective

2.1.1 To evaluate if the proposed patient care strategy, that includes use of simplified Guidelines along with APL expert support, decreases the one-month induction mortality rate from 30% to under 15%.

2.2 Secondary Objectives

2.2.1 To assess the overall survival 1 year after accrual is completed.

2.2.2 To assess incidence and severity of differentiation syndrome

2.2.3 To correlate outcomes with time to initiation of ATRA from diagnosis or suspicion of diagnosis.

2.2.4 To evaluate outcomes in academic and community centers separately.

2.2.5 To evaluate factors associated with outcome.

3. Selection of Patients

3.1 Eligibility Criteria

_____ 3.1.1 All patients ≥ 18 years of age who are confirmed to have a diagnosis of APL, which is defined as:

_____ 3.1.1.1 Positive t(15:17) by FISH or conventional karyotype

_____ 3.1.1.2 Positive PML/RAR alpha by PCR

_____ 3.1.2 Patients must accept treatment and supportive care guidelines..

_____ 3.1.3 Referrals must be made as early as possible by treating physician (provider) but no later than 5 calendar days after ATRA or APL directed therapy is initiated. Consent can be obtained up till day 7 or earlier.

_____ 3.1.4 Co-management can be started as soon as referral is made including weekends. The Physician at the outlying facility should make every effort to call the APL expert at the first suspicion of APL.

4. Registration Procedures

CTEP Investigator Registration Procedures

Food and Drug Administration (FDA) regulations and National Cancer Institute (NCI) policy require all investigators participating in any NCI-sponsored clinical trial to register and to renew their registration annually.

Registration requires the submission of:

- a completed ***Statement of Investigator Form*** (FDA Form 1572) with an original signature
- a current Curriculum Vitae (CV)
- a completed and signed ***Supplemental Investigator Data Form*** (IDF)
- a completed ***Financial Disclosure Form*** (FDF) with an original signature

Fillabe PDF forms and additional information can be found on the CTEP website at <http://ctep.cancer.gov/investigatorResources/investigator_registration.htm>. For questions, please contact the ***CTEP Investigator Registration Help Desk*** by email at <pmbregpend@ctep.nci.nih.gov>.

CTEP Associate Registration Procedures / CTEP-IAM Account

The Cancer Therapy Evaluation Program (CTEP) Identity and Access Management (IAM) application is a web-based application intended for use by both Investigators (i.e., all physicians involved in the conduct of NCI-sponsored clinical trials) and Associates (i.e., all staff involved in the conduct of NCI-sponsored clinical trials).

Associates will use the CTEP-IAM application to register (both initial registration and annual re-registration) with CTEP and to obtain a user account.

Investigators will use the CTEP-IAM application to obtain a user account only. (See CTEP Investigator Registration Procedures above for information on registering with CTEP as an Investigator, which must be completed before a CTEP-IAM account can be requested.)

An active CTEP-IAM user account will be needed to access all CTEP and CTSU (Cancer Trials Support Unit) websites and applications, including the CTSU members' website.

Additional information can be found on the CTEP website at <http://ctep.cancer.gov/branches/pmb/associate_registration.htm>. For questions, please contact the ***CTEP Associate Registration Help Desk*** by email at <ctepreghelp@ctep.nci.nih.gov>.

CTSU Registration Procedures

This study is supported by the NCI Cancer Trials Support Unit (CTSU).

IRB Approval:

Each investigator or group of investigators at a clinical site must obtain IRB approval for this protocol and submit IRB approval and supporting documentation to the CTSU Regulatory Office before they can be approved to enroll patients. Study centers can check the status of their registration packets by querying the Regulatory Support System (RSS) site registration status page of the CTSU members' website by entering

credentials at https://www.ctsu.org. For sites under the CIRB initiative, IRB data will automatically load to RSS.

Downloading Site Registration Documents:

Site registration forms may be downloaded from the **EA9131**protocol page located on the CTSU members' website. Add if a restricted access protocol: Permission to view and download this protocol and its supporting documents is restricted and is based on person and site roster assignment housed in the CTSU RSS.

- Go to https://www.ctsu.org and log in to the members' area using your CTEP-IAM username and password
- Click on the Protocols tab in the upper left of your screen
- Click on the **ECOG-ACRIN** link to expand, then select trial protocol **EA9131**
- Click on the Site Registration Documents link

Requirements for EA9131 Site Registration:

- CTSU IRB Certification
- CTSU IRB/Regulatory Approval Transmittal Sheet (for sites not participating via the NCI CIRB)

Submitting Regulatory Documents

Submit completed forms along with a copy of your IRB Approval and Model Informed Consent to the CTSU Regulatory Office, where they will be entered and tracked in the CTSU RSS.

CTSU Regulatory Office
1818 Market Street, Suite 1100
Philadelphia, PA 19103
Phone: 1-866-651-2878
FAX: (215) 569-0206
E-mail: CTSURegulatory@ctsu.coccg.org (for regulatory document submission only)

Required Protocol Specific Regulatory Documents

1. CTSU Regulatory Transmittal Form.
2. Copy of IRB Informed Consent Document.

NOTE: At the main sites, the PI and other designated investigators will obtain the consent.

If a patient is being treated at an outlying site, the local PI or Co/Sub-PI will consent the patient. The treating physician at the outlying site will contact an APL expert as soon as the diagnosis is suspected but no later than 5 days after ATRA has been initiated.

NOTE: Any deletion or substantive modification of information concerning risks or alternative procedures contained in the sample informed consent document must be justified in writing by the investigator and approved by the IRB.

3. A. CTSU IRB Certification Form.
   Or
   B. Signed HHS OMB No. 0990-0263 (replaces Form 310).
   Or
   C. IRB Approval Letter

   NOTE: The above submissions must include the following details:
   - Indicate all sites approved for the protocol under an assurance number.
   - OHRP assurance number of reviewing IRB
   - Full protocol title and number
   - Version Date
   - Type of review (full board vs. expedited)
   - Date of review.
   - Signature of IRB official

Checking Your Site's Registration Status:

Check the status of your site's registration packets by querying the RSS site registration status page of the members' section of the CTSU website.

NOTE: Sites will not receive formal notification of regulatory approval from the CTSU Regulatory Office.

- Go to https://www.ctsu.org and log in to the members' area using your CTEP-IAM username and password
- Click on the Regulatory tab at the top of your screen
- Click on the Site Registration tab
- Enter your 5-character CTEP Institution Code and click on Go

Patient enrollment will be facilitated using the Oncology Patient Enrollment Network (OPEN). OPEN is a web-based registration system available on a 24/7 basis. To access OPEN, the site user must have an active CTEP-IAM account (check at < https://eapps-ctep.nci.nih.gov/iam/index.jsp >) and a 'Registrar' role on either the LPO or participating organization roster.

The Study Chair Liaison will use OPEN to enroll patients to this study. It is integrated with the CTSU Enterprise System for regulatory and roster data, and upon enrollment, initializes the patient in the Rave database. OPEN can be accessed at https://open.ctsu.org or from the OPEN tab on the CTSU members' side of the website at https://www.ctsu.org.

Patient enrollment for this study will be facilitated using the Slot-Reservation System in conjunction with the Registration system in the Oncology Patient Enrollment Network (OPEN). Prior to discussing protocol entry with the patient, all site staff must contact the Study Chair Liaison, who will use the CTSU OPEN Slot Reservation System to insure that a slot on the protocol is available to the patient. Once a slot- reservation confirmation is obtained, site staff may then proceed to consent the patient to this study. Upon receipt of the consent form, the Study Chair Liaison will then enroll the patient.

Prior to accessing OPEN, site staff should verify the following:

- All eligibility criteria have been met within the protocol stated timeframes.

- All patients have signed an appropriate consent form and HIPAA authorization form (if applicable).

NOTE: The OPEN system will provide the site with a printable confirmation of registration and treatment information. The Study Chair Liaison will print this confirmation and provide it to the site for records.

Further instructional information is provided on the OPEN tab of the CTSU members' side of the CTSU website at https://www.ctsu.org or at https://open.ctsu.org. For any additional questions contact the CTSU Help Desk at 1-888-823-5923 or ctsucontact@westat.com.

4.1 Protocol Number

4.2 Investigator Identification

- Institution and affiliate name
- Investigator's name

4.3 Patient Identification

- Patient's initials (first and last)
- Patient's Hospital ID and/or Social Security number
- Patient demographics
    - Gender
    - Birth date (mm/yyyy)
    - Race
    - Ethnicity
    - Nine-digit ZIP code
    - Method of payment
    - Country of residence

4.4 Eligibility Verification

Patients must meet all of the eligibility requirements listed in Section 3.

4.5 Additional Requirements

4.5.1 Patients must provide a signed and dated, written informed consent form.

NOTE: Copies of the consent are not collected by the ECOG-ACRIN Operations Office

4.5.1.1 Data collection for this study will be done exclusively through the Medidata Rave clinical data management system. Access to the trial in Rave is granted through the iMedidata application to all persons with the appropriate roles assigned in Regulatory Support System (RSS). To access Rave via iMedidata, the site user must have an active CTEP-IAM account (check at < https://eapps-ctep.nci.nih.gov/iam/index.jsp >) and the appropriate Rave role (Rave CRA, Read-Only, Site

Investigator) on either the LPO or participating organization roster at the enrolling site.

Upon initial site registration approval for the study in RSS, all persons with Rave roles assigned on the appropriate roster will be sent a study invitation e-mail from iMedidata. To accept the invitation, site users must log into the Select Login (https://login.imedidata.com/selectlogin) using their CTEP-IAM user name and password, and click on the "accept" link in the upper right-corner of the iMedidata page. Please note, site users will not be able to access the study in Rave until all required Medidata and study specific trainings are completed. Trainings will be in the form of electronic learnings (eLearnings), and can be accessed by clicking on the link in the upper right pane of the iMedidata screen.

Users that have not previously activated their iMedidata/Rave account at the time of initial site registration approval for the study in RSS will also receive a separate invitation from iMedidata to activate their account. Account activation instructions are located on the CTSU website, Rave tab under the Rave resource materials (Medidata Account Activation and Study Invitation Acceptance). Additional information on iMedidata/Rave is available on the CTSU members' website under the Rave tab at www.ctsu.org/RAVE/ or by contacting the CTSU Help Desk at 1-888-823-5923 or by e-mail at ctsucontact@westat.com.

5. Methodology Plan

There will be 7 investigators at 6 different centers (LEAD SITES) who will serve as the APL experts for this proposal. The patients treated at these 6 centers will be enrolled at their particular institution. **The study will not be open at other academic centers**. The APL expert at each site will either manage the patient or provide consultative oversight to the treating team. The sub-investigators at lead academic sites can consult patients but it is expected that the local PI will provide oversight. We expect to enroll a maximum of 30% of the patients at these academic sites. Induction mortality is a problem in larger centers as well from extensive data that has been published. We postulate that using the guidelines along with expert input will improve outcome in lead sites. This will give us the opportunity to assess the problem as well as outcome in academic centers and compare the results to community centers. Since the accrual goal is 200 patients over a 4 year period, the accrual at lead academic centers will be limited to 15 (30%) patients per year. The Study Chair and ECOG-ACRIN office will monitor this closely and once the target is achieved in a year, enrollment at lead sites will be put on hold. Investigators from the LEAD sites will meet or have a conference call twice a year to discuss the progress and results.

Prior to enrolling patients, while the study is being reviewed by the IRBs at the respective sites, the study investigators will visit practices that are likely to treat APL in their catchment area. These visits will also be a part of publicizing the trial to ensure other treating physicians are aware of the study and also the risk of early deaths in this disease. In addition to physician visits, flyers and regular emails will also be sent out to ensure that there are frequent reminders about the study. At sites that open the trial there will also be a webinar to raise awareness for the trial and provide training on the treatment guidelines prior to study activation.

The trial will be conducted at NCORP community sites in addition to the Lead Academic Sites. We conducted a survey of NCORP sites to assess their interest in participating in the study. The survey had 3 questions. a. Does your center treat patients with APL? b. Are you aware the induction mortality in APL is approximately 30%? c. Will your Center participate in a trial focused on decreasing induction mortality? Eleven sites responded and expressed interest. The PI and Co-PI will make personal contact with the other NCORP sites and present the goals of the trial and get other sites to open. We have also asked the NCORP leadership to give us an opportunity to participate in their annual meeting and conference calls and publicize the study to get more sites to participate.

5.1 Lead Sites

5.1.1 Lead Sites

- Winship Cancer Institute of Emory University
- Memorial Sloan-Kettering Cancer Center, New York, NY
- The Mayo Clinic, Rochester, MN
- Northwestern University Feinberg School of Medicine, Chicago, IL
- Abramson Cancer Center of the University of Pennsylvania, Philadelphia, PA
- The Mayo Clinic, Jacksonville, FL

5.1.2 Patients treated at Lead Sites will be treated locally at the Lead Site using treatment guidelines. The local PI will provide expert support to

the treating team. The presentation of APL can be highly variable; hence adherence to the guidelines is not always feasible. Deviations that are made from the guidelines based on particular clinical situations will be documented in the medical record.

5.2 Outlying Facility

5.2.1 Any subjects that are treated and have a treating physician outside of the Lead Sites will be considered a subject at an Outlying Facility. The treating physician from the Outlying Facility should call the APL Expert in their catchment area for treatment guidance. Seeking advice from an APL expert is a key component of our model. The call should be made as soon as APL is suspected but no later than 5 days after starting ATRA and communication should be ongoing based on the clinical developments till a hematologic remission is achieved. The presentation in APL can be highly variable; hence complete adherence to the guidelines is not always feasible. Deviations that are made from the guidelines based on particular clinical situations will be documented in the medical record.

5.2.1.1 The provider at outlying sites will be the Hematologist/Oncologist attending caring for the patient. Initial contact has to be made by a Hematologist/Oncologist.

5.2.1.2 It is expected that the initial contacting Hematologist/Oncologist will continue the communication; alternatively, other members of the practice can communicate with the APL expert on an ongoing basis.

5.2.1.3 Trainees or advanced level practice providers (physician assistants or nurse practitioners) and trainees can be in communication subsequently with APL expects to continue the care.

5.2.2 A log will be maintained by the APL expert (Please see Appendix II). This will include information on Date admitted, date APL suspected, date APL confirmed, date APL therapy started and date APL expert was contacted. Subsequent calls will be documented on the log sheet.

5.2.3 It will be suggested that the treating physician will also document the communication on an ongoing basis.

5.3 Study Accrual and Timeline

The study will accrue from years one to 4. Follow up will be for one year after accrual is completed. The total study duration is 5 years.

5.4 Adverse Event Reporting Requirements

The adverse events to treatment will be collected from the hospital records obtained from the patients induction period and also follow up at the treating physician's office. An AE log need not be maintained since this study will not include any new drugs. The complications from treatment will be noted in the database and will be used for analysis of outcomes. The only SAE that has to be reported is death.

5.4.1 Reporting Procedure

This study requires that expedited serious adverse event reporting use CTEP's Adverse Event Reporting System (CTEP-AERS). The CTEP's guidelines for CTEP-AERS can be found at http://ctep.cancer.gov. A CTEP-AERS report must be submitted electronically to ECOG-ACRIN and the appropriate regulatory agencies via the CTEP-AERS Web-based application located at http://ctep.cancer.gov.

In the rare event when Internet connectivity is disrupted a 24-hour notification is to be made by telephone to

- the SAE Team at ECOG-ACRIN (617-632-3610)
- the NCI (301-897-7497)

An electronic report MUST be submitted immediately upon re-establishment of internet connection.

Supporting and follow up data: Any supporting or follow up documentation must be uploaded to the Supplemental Data Folder in Medidata Rave within 48-72 hours. In addition, supporting or follow up documentation must be faxed to the NCI (301- 230-0159) in the same timeframe.

CTEP Technical Help Desk: For any technical questions or system problems regarding the use of the CTEP-AERS application, please contact the NCI Technical Help Desk at ncictephelp@ctep.nci.nih.gov or by phone at 1-888-283-7457.

5.4.2 Expedited Reporting Requirements for EA9131

**Grade 5**[a]	
Unexpected	Expected
24-Hour 5 Calendar Days	24-Hour 5 Calendar Days
24-Hour 5 Calendar Days	24-Hour 5 Calendar Days
**24-Hour; 5 Calendar Days** – The AE must initially be reported via CTEP-AERS within 24 hours of learning of the AE, followed by a complete expedited report within 5 calendar days of the initial 24-hour report.	
[a] This includes all deaths within one year of registration, regardless of cause. Thereafter, deaths must only be reported on the protocol specific case report forms.	

5.4.3 Other recipients of adverse event reports and supplemental data

ECOG-ACRIN will forward CTEP-AERS reports to the appropriate regulatory agencies and pharmaceutical company, if applicable.

Serious Adverse events determined to be reportable via CTEP-AERS must also be reported by the institution, according to the local policy and procedures, to the Institutional Review Board responsible for oversight of the patient.

5.5 Completion of Induction

Induction will be continued until the patient achieves hematologic remission (as per guidelines).

5.6 Consolidation

Consolidation will be at the treating physician's discretion.

5.7 Maintenance

Maintenance will be per treating physician's discretion.

5.8 Duration of Follow-up

For this protocol, all patients, including those who discontinue treatment early, will be followed for survival for 1 year after confirmed diagnosis.

6. Study Parameters

NOTE: Please refer to Appendix I for all recommended tests and procedures

	Baseline
Confirmed APL Dianosis[1]	X
Referral to APL Expert[2]	X
Informed Consent[3]	X
Subject Registration[4]	X

1. Positive t(15:17) by FISH or conventional karyotype and PML/RARα by PCR, rare variants can also be included if confirmed.
2. It is preferred that the APL expert for the catchment area is contacted, however, any APL Expert at any Lead Site may be contacted. Referrals must be made within 5 calendar days after ATRA or APL directed therapy is initiated.
3. Written informed consent must be obtained within 7 days of confirmed diagnosis.
4. Registration can be done after initiating treatment.

7. Statistical Considerations

7.1 Study Design and Objectives

This study will accrue 200 APL patients over a 4-year period. The primary hypothesis is that the proposed process will lower the one-month mortality rate from 30% to < 15%. The primary endpoint is the one-month mortality rate Secondary endpoints include: i) overall survival, one-year survival rate, ii) safety, iii) initiation of treatment from diagnosis, iv) survival in academic and community centers v) compliance with treatment guidelines and vi) evaluation of factors associated with outcome.

7.2 Sample Size Considerations and Monitoring Plan

Of the 200 patients accrued, it is expected to have 180 eligible patients after adjusting for ineligible and/or lost-to-follow up cases. We allowed 10% for ineligible and/or lost-to-follow-up cases. We often allow additional cases (up to 10%) to account for ineligible cases in ECOG studies. We do not expect any lost-to-fu cases at 1 month. However, we used 10% ineligibility and/or lost-to-follow-up to be conservative and general in this study.

After 100 patients (for 90 eligible) are enrolled and have followed for one-month mortality endpoint, one interim analysis will be conducted. Using the critical value corresponding to the O'Brien and Fleming boundary at 50% information time, 95% Repeated Confidence Interval (RCI) for the one-month mortality rate will be constructed. The result will be discussed with the ECOG-ACRIN DSMC members. If deemed efficacious, accrual will continue to 200 (for 180 eligible) patients. This design will have at least 90% power with a two-sided type I error rate of < 0.05. Another rationale for the sample size of 200 is that we would like to build a 95% RCI with a tight interval. For the expected one-month mortality rate, if the observed rate is 10% (18/180) the 95% RCI will be 6-15.4%.If the observed one-month mortality rate is 15%, the 95% RCI will be 10.1-21.1%.

We plan to limit the accrual from the large academic centers to a maximum of 30% of the total patients. Thus 60 patients (54 eligible) from major academic centers and 140 (126 eligible) from NCORP community sites are anticipated. The primary analysis and monitoring will be based on all patients regardless of enrollment site. After the study is completed, one-month mortality rate will be estimated for patients enrolled from NCORP and academic sites separately as a secondary analysis. With 126 eligible patients from NCORP sites, there will be at least 90% power for the hypothesis testing of reducing mortality rate from 30% to 15%, with a two-sided error rate of 0.05. There is sufficient power to test if one-month mortality rate for NCORP sites can be reduced from 30% to < 15%

For NCORP sites, 95% CI is 5.6-17% for the observed one-month mortality rate of 10.3% (13/126) and 95% CI is 9.3-22.5% for the observed one-month mortality rate of 15.1%. (19/126). With 54 eligible patients from academic sites, there will be less than 80% power for the hypothesis testing of reducing mortality rate from 30% to 15%, with a two-sided error rate of 0.05. For academic sites, 95% CI is 3-20.3%% for the observed one-month mortality rate of 9.3% (5/54) and 95% CI is 6.6-27.1% for the observed one-month mortality rate of 14.8%. (8/54). Statistical analysis will be mostly descriptive for the secondary endpoints.

At 50% information time (i.e. 90 eligible patients have one-month mortality assessed), 95% Repeated Confidence Interval will be built for the one-month mortality estimate using the O'Brien-Fleming boundary for 50% information time. This analysis will be presented to the ECOG-ACRIN DSMC and discussed further.

We do not expect major safety issues in this study. However adverse events data will be monitored in ongoing basis and will be brought to ECOG-ACRIN DSMC attention.

7.3 Statistical Analysis Plan

7.3.1 Primary Objective

One-month mortality rate will be estimated by dividing total number of deaths within one month of study entry by total number of cases with at least one-month follow up data. 95% RCI will be provided for this endpoint. We will also evaluate the OS data using the Kaplan-Meier plot.

7.3.2 Secondary Objectives

Using the method of Kaplan-Meier, overall survival (OS) data will be described. The median OS and one-year OS rate will be estimated and 95% CI will be provided.

We confirm that the primary objective is to assess one-month mortality rate among all cases. Secondary analysis will involve evaluating outcome data in NCORP and academic sites separately. We clarified this further in Section 7.2. Overall the expected one-month mortality rate is in the range of 10-15% across all patients as well as in two subgroups of NCORP and academic sites.

With 200 (180 eligible) patients, we expect to build a confidence interval not wider than 11% for the expected one-month mortality rate of 10-15%. With 140 (126 eligible) cases from NCORP sites, the confidence interval for the one-month mortality rate will be not be wider than 13%.

For the safety endpoints, the severity and duration of coagulopathy, bleeding, infections and differentiation syndrome will be measured based on the CTC version 4.0. Analysis of the safety data will be mostly descriptive. The proportion of patients with the worst degree toxicity of grade 3 or higher will be assessed and presented with 95% CI. The length of hospital stay is defined as a period between the discharge and admission dates for the first hospitalization. For patients with no hospitalization, this will be defined as 0. To assess the associations between the length of hospital stay and toxicity measures (bleeding, infections, differentiation syndrome), each toxicity measures will be dichotomized as "mild" vs. "severe" and the length of hospital stay will be compared using the two-sample t-test.

The time from diagnosis and initiation of treatment will be assessed and dichotomized as "short' vs. "long" using the median value. One-month mortality rate will be compared in these two groups (short vs. long) using the Fisher's exact test. Overall-survival will also be

compared in these two groups using the logrank test. In this analysis, the time of diagnosis will be determined by both: i) confirmed diagnosis date and ii) date of suspicious APL. The dichotomized value of long vs. short based on the both times will be evaluated. Similarly the one-month morality rate and overall survival between the lead institutions and outlying centers will be compared. For all comparisons, two-sided p-values will be reported.

Compliance with treatment guidelines will be assessed as binary outcome and the successful compliance rate will be described with 95% confidence interval. A regression model will be developed to evaluate the factors associated with outcome. Data items such as age, risk status, comorbidity will be collected and evaluated.

8. Electronic Data Capture

Please refer to the **EA9131** Forms Completion Guidelines for the forms submission schedule. Data collection will be performed exclusively in Medidata Rave. In case of withdrawal from the study, data will be collected to the point of withdrawal.

9. Patient Consent and Peer Judgment

Current FDA, NCI, state, federal and institutional regulations concerning informed consent will be followed.

10. References

1. Lehmann S, Ravn A, Carlsson L, Antunovic P, Deneberg S, Mollgard L, et al. Continuing high early death rate in acute promyelocytic leukemia: a population-based report from the Swedish Adult Acute Leukemia Registry. Leukemia. 2011;25(7):1128-34.
2. Park JH, Qiao B, Panageas KS, Schymura MJ, Jurcic JG, Rosenblat TL, et al. Early death rate in acute promyelocytic leukemia remains high despite all-trans retinoic acid. Blood. 2011;118(5):1248-54.
3. Chen Y, Kantarjian H, Wang H, Cortes J, Ravandi F. Acute promyelocytic leukemia: a population-based study on incidence and survival in the United States, 1975-2008. Cancer. 2012;118(23):5811-8.
4. Jacomo RH, Melo RA, Souto FR, de Mattos ER, de Oliveira CT, Fagundes EM, et al. Clinical features and outcomes of 134 Brazilians with acute promyelocytic leukemia who received ATRA and anthracyclines. Haematologica. 2007;92(10):1431-2.
5. McClellan JS, Kohrt HE, Coutre S, Gotlib JR, Majeti R, , et al.Treatment advances have not improved the early death rate in acute promyelocytic leukemia. Haematologica.2012;97(1):133-6.
6. Bajpai J1, Sharma A, Kumar L, Dabkara D, Raina V, Kochupillai V, Kumar R. Acute promyelocytic leukemia: an experience from a tertiary care centre in north India. Indian J Cancer. 2011 Jul-Sep;48(3):316-22.
7. Imagawa J, Harada Y, Shimomura T, Tanaka H, Okikawa Y, Harada H. High early death rate in elderly patients with acute promyelocyticleukemia treated with all-trans retinoic acid combined chemotherapy. Int J Hematol. 2013 Aug;98(2):264-6.
8. Serefhanoglu, S., et al. (2010). "Clinical features and outcomes of 49 Turkish patients with acute promyelocytic leukemia who received ATRA and anthracyclines (PETHEMA protocol) therapy." Leuk Res 34(12): e317-319
9. Lengfelder, E., et al. (2013). "Outcome of elderly patients with acute promyelocytic leukemia: results of the German Acute Myeloid Leukemia Cooperative Group." Ann Hematol 92(1): 41-52
10. Anand P. Jillella, Ibrahim Sadek, Devi Morrison, Kavita Natrajan, Farrukh Tauseef Awan, Ravindra Kolhe, Vamsi Kota. A simple but effective model to decrease early deaths in acute promyelocytic leukemia (APL). J Clin Oncol 30, 2012 (suppl; abstr 6573)
11. Rego EM, Kim HT, Ruiz-Arguelles GJ, Undurraga MS, Uriarte Mdel R, Jacomo RH, et al. Improving acute promyelocytic leukemia (APL) outcome in developing countries through networking, results of the International Consortium on APL. Blood. 2013;121(11):1935-43.

A Simplified Patient Care Strategy to Decrease Early Deaths in Acute Promyelocytic Leukemia (APL)

Appendix I

Acute Promyelocytic Leukemia Treatment Guidelines

Developed at Georgia Regents University, August 2013

AUTHORS: Dr. Anand Jillella Mobile: 706 -951- 5144 Office: 404-778-1900
Dr. Vamsi Kota Mobile: 706- 825- 4091 Office: 404-778-1900

WORK-UP

BASELINE

- Chest X-ray,
- Echocardiogram and EKG
- PICC line placement – **NO** central lines or invasive procedures (bronchoscopy, spinal tap)
- Bone marrow examination (aspirate, biopsy, flow, cytogenetics, FISH for PML-RARα, PCR for PML-RARα)

  Day 14 marrow is **NOT** necessary.

DAILY

- CBC, CMP, PT, PTT, fibrinogen **TWICE DAILY** until lab & clinical coagulopathy is resolved
- D-dimer **ONCE DAILY** for **ENTIRE** hospitalization.

PREVENTION/TREATMENT OF COAGULOPATHY

- Intracranial, pulmonary and GI hemorrhages are frequent
- Risk of hemorrhage is ↑ with the presence of any of the following - active sites of bleeding, ↓ fibrinogen, ↑ D-dimers, ↑ PT & PTT, ↑ WBC, ↑peripheral blasts, renal failure and poor performance status;
- **Keep platelets above 50,000**
- If there are any active sites of bleeding on presentation (e.g., needle sticks, bone marrow biopsy sites), give 4 units of fresh frozen plasma (FFP) at the start of ATRA and chemotherapy. **CONTINUE** FFP support **TWICE A DAY** until clinical bleeding resolves
- **Keep fibrinogen above 150**. Use cryoprecipitate if needed.
- When clinical & lab coagulopathy is resolved, blood product support is per standard guidelines.

APL IS A MEDICAL EMERGENCY - START ATRA ASAP

PREVENTION/TREATMENT OF APL DIFFERENTIATION SYNDROME

Differentiation Syndrome-symptoms: dyspnea, unexplained fever, ↑ weight, peripheral edema, hypotension, acute renal failure, CHF, pleuropericardial effusions, and interstitial pulmonary infiltrates.

- Daily weights - **BEDSIDE SCALES ONLY**
- Keep I/O matched – **METICULOUSLY**
- Diuretics if ↑fluid retention or ↑weight
- Dexamethasone 10 mg BID if onset of DS symptoms

- If WBC >10,000, dexamethasone 10 mg bid could be started before initiating ATRA
- Temporary discontinuation of ATRA or Arsenic Trioxide (ATO) is indicated only in severe cases of DS.
- Continue dexamethasone until 3 days after all symptoms are resolved. ATRA and/or arsenic can be resumed

SUPPORTIVE CARE

- Allopurinol 300 mg daily
- Antibiotic prophylaxis - levofloxacin 500 mg po daily or similar antibiotic
- Antifungal prophylaxis - posaconazole 200 mg po 3x daily, voriconazole 200 mg po 2x daily, Micafungin 50 mg daily or a similar drug
- Anti-viral prophylaxis - acyclovir 400 mg po 2x daily or valacyclovir 1000 mg PO daily
- RBC transfusion recommended when HGB ↓ 8.

ANTHRACYCLINE BASED INDUCTION

INDUCTION OF LOW AND INTERMEDIATE RISK PATIENTS

- **WBC < 10,000 and PLTS >40,000 or <40,000 -** AIDA protocol.
- ATRA on Day 1.
- Idarubicin 12 mg/m^2 on Days 2, 4, 6 and 8.
- ATRA at 45 mg/m^2 DAILY in divided doses twice a day until complete hematologic remission.
  OR
- ATRA 45 mg/M2; in divided doses twice a day plus ATO 0.15 mg/KG daily until complete hematologic remission (no anthracycline). Prednisone 0.5 mg/Kg for 14 days followed by a gradual taper if no evidence of DS.

INDUCTION OF HIGH RISK PATIENTS

- **WBC >10,000 and PLTS <40,000** - ATRA to be started as soon as diagnosis is suspected.
- Idarubicin 12 mg/m^2 to be started on the SAME DAY and given per the AIDA protocol on days 1, 3, 5 and 7.
- ATRA at 45 mg/m^2 in divided doses twice a day till complete hematologic remission
- Even if the genetic results are not available, it is reasonable to start Anthracycline.

ARSENIC TRIOXIDE USE IN INDUCTION

CONSIDER ATO

- Low or intermediate risk patient
- Older patients and patients with comorbid conditions
- Not a candidate for conventional chemotherapy for any reason.
- Arsenic at 0.15 mg/kg daily, continued till complete hematologic remission
- Combine with ATRA 45mg/m^2 DAILY in divided doses
- Watch for differentiation syndrome
- Watch for prolonged QT interval
- Keep Mg above 2.0 and K above 4.0
- Follow LFTs for grade 2 - 4 liver dysfunction. If this occurs HOLD arsenic.

SUGGESTED MANAGEMENT OF ARSENIC-INDUCED LEUKOCYTOSIS

- WBC 5 – 10k - hydroxyurea 500 mg q day
- WBC 10 – 15k - hydroxyurea 500mg BID
- WBC 15 – 20k - hydroxyurea 500mg TID
- WBC 20 – 50k - hydroxyurea 500 mg QID
- WBC > 50k - hydroxyurea 1000 mg QID
- If leukocytosis does not resolve one or two doses of Idarubicin could be considered
- **NO LEUKOPHERESIS**.

ELDERLY PATIENTS AND PATIENTS WITH SEVERE COMORBID CONDITIONS

- May not be candidates for cytotoxic chemotherapy
- Consider dose reduction of ATRA
- Consider dose reduction of ATO

A Simplified Patient Care Strategy to Decrease Early Deaths in Acute Promyelocytic Leukemia (APL)

Appendix II

APL Expert and NCORP Provider Communication

1. Date patient admitted -
2. Date APL suspected -
3. Date APL confirmed -
4. Date APL therapy started -
5. Date APL expert contacted -
6. Subsequent communications -

Date:

Date:

Date:

Date:

Date:

Date:

Date:

Date:

Date:

Date:

A Simplified Patient Care Strategy to Decrease Early Deaths in Acute Promyelocytic Leukemia (APL)

Appendix III

NCORP PROVIDER INFORMATION SHEET

This is a research study focused on decreasing induction mortality in Acute Promyelocytic Leukemia (APL). Historical data shows an induction mortality of 30% in the general population and the purpose of this study is to decrease it to < 15%. NCORP providers will be given this information sheet along with the treatment guidelines in Appendix I.

A. The study will consist of using the simplified APL treatment algorithm that is attached.

B. Equally important is for the provider at NCORP sites to call an APL expert listed in the protocol or the PI/Co-PI. Cell phone numbers of APL experts are in the Protocol.

C. The APL expert will function as a resource during induction to prevent and manage complications. It is suggested that the provider at NCORP sites call the APL expert as frequently as needed during the first 4 weeks. The experts are available 24/7.

D. NCORP providers will document discussion with the APL expert and clinical decisions in the Medical Record. APL experts will fill the Expert/NCORP Provider communication sheet.

E. There are no obvious discomforts to the patient since the patient will be treated on the accepted standard of care. Expert support may help in preventing and managing complications and improving outcome.

F We will retain all patient records and signed informed consent forms filed in a secure location. Database is maintained using web-based database system. Only research members are granted access to web-based database system with user ID and password.

G. Participation is voluntary and the patient may choose not to participate in the trial. In that case the patient will be treated per usual treatment guidelines. Participants may discontinue at any time without penalty or loss of benefits.

A Simplified Patient Care Strategy to Decrease Early Deaths in Acute Promyelocytic Leukemia (APL)

Appendix IV

Patient Thank You Letter

We ask that the physician use the template contained in this appendix to prepare a letter thanking the patient for enrolling in this trial. The template is intended as a guide and can be downloaded from the web site at http://www.ecog.org. As this is a personal letter, physicians may elect to further tailor the text to their situation.

This small gesture is a part of a broader program being undertaken by ECOG-ACRIN and the NCI to increase awareness of the importance of clinical trials and improve accrual and follow-through. We appreciate your help in this effort.

[PATIENT NAME] [DATE]

[PATIENT ADDRESS]

Dear Mr. / Mrs. / Ms. ___________,

Thank you for agreeing to take part in this important research study. Many questions remain unanswered in cancer. With the participation of people like you in clinical trials, we will improve treatment and quality of life for those with your type of cancer.

We believe you will receive high quality, complete care. I and my research staff will maintain very close contact with you. This will allow me to provide you with the best care while learning as much as possible to help you and other patients.

On behalf of ____________________ and ECOG-ACRIN, we thank you again and look forward to helping you.

Sincerely,

[PHYSICIAN NAME]

EA9131

A Simplified Patient Care Strategy to Decrease Early Deaths in Acute Promyelocytic Leukemia (APL)

STUDY CHAIR:	Anand P. Jillella, MD
STUDY CO-CHAIR:	Vamsi K. Kota, MD
STUDY STATISTICIAN:	Sandra Lee, ScD
PREVENTION COMMITTEE CHAIR:	Raymond Bergan, MD
CANCER CARE DELIVERY RESEARCH CHAIR:	Ruth Carlos, MD
PATIENT OUTCOMES & SURVIVORSHIPCHAIR:	Lynne I. Wagner, PhD
LEUKEMIA COMMITTEE CHAIR:	Mark R. Litzow, MD

Version Date: June 19, 2017

STUDY PARTICIPANTS	***ACTIVATION DATE***
**ALLIANCE /** Alliance for Clinical Trials in Oncology, NCORP Sites Only	***TBD***
**ECOG-ACRIN /** NCORP Sites Only	
**NRG /** NRG Oncology, NCORP Sites Only	
**SWOG /** SWOG, NCORP Sites Only	
**COG /** Children's Oncology Group, NCORP Sites Only	

NCTN participation is limited to the following sites:

FL080 / Mayo Clinic, Jacksonville (ECOG-ACRIN)
MN026 / Mayo Clinic, Rochester (ECOG-ACRIN)
NY016 / Memorial Sloan-Kettering Cancer Center (ECOG-ACRIN)
IL036 / Northwestern University (ECOG-ACRIN)
PA075 / University of Pennsylvania (ECOG-ACRIN)

STUDY CHAIR

Anand P. Jillella, MD
Georgia Cancer Center
Medical College of Georgia at Augusta University
1120 15th Street, BAA 5407
Augusta, GA, 30912
Office Phone: 706-721-2505
Cell Phone: 706-951-5144
Fax: 706-721-5566
Email: TBD

STUDY CO-CHAIR

Vamsi K. Kota, MD
Winship Cancer Institute of Emory University
1365 Clifton Road NE, Room C1152
Atlanta, GA 30322
Office Phone: 404-778-1900
Cell Phone: 706-825-4091
Fax: 404-778-4755
Email: vkota@emory.edu

STUDY CHAIR LIAISON (SCL)

Prachi Karkhanis
Winship Cancer Institute of Emory University
1365 Clifton Road NE, Room C1152
Atlanta, GA 30322
Office Phone: 404-778-3663
Fax: 404-778-4755

Email: prachi.karkhanis@emory.edu

ECOG-ACRIN STUDY EXPERTS

Anand P. Jillella, MD
Georgia Cancer Center
Medical College of Georgia at Augusta University
1120 15th Street, BAA 5407
Augusta, GA, 30912
Office Phone: 706-721-2505
Cell Phone: 706-951-5144
Fax: 706-721-5566
Email: TBD

Jessica K. Altman, MD
Northwestern University Feinberg School of Medicine
Robert H. Lurie Comprehensive Cancer Center
303 E. Superior Street, Chicago, IL 60611
Office Phone: 312-503-1817
Cell Phone: 773-575-2598
Fax: 312-695-6189
Email: j-altman@northwestern.edu

Vamsi K. Kota, MD
Winship Cancer Institute of Emory University
1365 Clifton Road NE, Room C1152
Atlanta, GA 30322
Office Phone: 404-778-1900
Cell Phone: 706-825-4091
Fax: 404-778-4755
Email: vkota@emory.edu

Selina Luger, MD
Abramson Cancer Center of the University of Pennsylvania
2 West, Perelman Center for Advanced Medicine
3400 Civic Center Road, Philadelphia, PA, 19104
Office Phone: 215-662-6348
Cell Phone: 215-279-0657
Fax: 215-662-4064
Email: selina.luger@uphs.upenn.edu

Martin S. Tallman, MD
Memorial Sloan-Kettering Cancer Center
Leukemia Service, Department of Medicine
1275 York Avenue, New York, NY 10065
Office Phone: 212-639-3842
Cell Phone: 646-467-4670
Fax: 212-639-3841
Email: tallmanm@mskcc.org

James M. Foran, MD, FRCPC
Mayo Clinic Florida
Division of Hematology & Medical Oncology
4500 San Pablo Road, Jacksonville, FL 32224
Office Phone: 904-953-7292
Cell Phone: 904-955-3679
Fax: 904-953-2315
Email: foran.james@mayo.edu

Mark R. Litzow, MD
The Mayo Clinic
Division of Hematology
200 First Street, SW, Rochester, MN 55905
Office Phone: 507-266-0523
Cell Phone: 507-269-4848
Fax: 507-266-4972
Email: litzow.mark@mayo.edu

Table of Contents

Schema 6
1. Introduction 7
 1.1 Rationale for Proposed Study 7
 1.2 Significance of the Study 9
 1.3 Study Overview 11
2. Objectives 12
 2.1 Primary Objective 12
 2.2 Secondary Objectives 12
3. Selection of Patients 13
 3.1 Eligibility Criteria 13
4. Registration Procedures 14
 4.1 Protocol Number 17
 4.2 Investigator Identification 17
 4.3 Patient Identification 17
 4.4 Eligibility Verification 17
 4.5 Additional Requirements 17
5. Methodology Plan 19
 5.1 Lead Sites 19
 5.2 Outlying NCORP Community Facility 20
 5.3 Study Accrual and Timeline 20
 5.4 Adverse Event Reporting Requirements 20
 5.5 Completion of Induction 22
 5.6 Consolidation 22
 5.7 Maintenance 22
 5.8 Duration of Follow-up 22
6. Study Parameters 23
7. Statistical Considerations 24
 7.1 Study Design and Objectives 24
 7.2 Sample Size Considerations and Monitoring Plan 24
 7.3 Statistical Analysis Plan 25
8. Electronic Data Capture 27
9. Patient Consent and Peer Judgment 27
10. References 27
Appendix I Acute Promyelocytic Leukemia Treatment Guidelines 28
Appendix II APL Expert and NCORP Provider Communication 31
Appendix III NCORP PROVIDER INFORMATION SHEET 32
Appendix IV Patient Thank You Letter 33

CANCER TRIALS SUPPORT UNIT (CTSU) ADDRESS AND CONTACT INFORMATION

**To submit site registration documents:**	**For patient enrollments:**	**Submit study data**
CTSU Regulatory Office<br>1818 Market Street, Suite 1100<br>Philadelphia, PA 19103<br>Phone – 1-866-651-CTSU<br>Fax – 215-569-0206<br>Email:<br>CTSURegulatory@ctsu.coccg.org<br>(for submitting regulatory documents only)	Please refer to the patient enrollment section of the protocol for instructions on using the Oncology Patient Enrollment Network (OPEN) which can be accessed at https://www.ctsu.org/OPEN_SYSTEM/ or https://OPEN.ctsu.org.<br>Contact the CTSU Help Desk with any OPEN-related questions at ctsucontact@westat.com.	Data collection for this study will be done exclusively through Medidata Rave.<br>Please see the data submission section of the protocol for further instructions.
The most current version of the **study protocol and all supporting documents** must be downloaded from the protocol-specific Web page of the CTSU Member Web site located at https://www.ctsu.org. Access to the CTSU members' website is managed through the Cancer Therapy and Evaluation Program - Identity and Access Management (CTEP-IAM) registration system and requires user log on with CTEP-IAM username and password.		
**For clinical questions (i.e., patient eligibility or treatment-related)** Contact the Study PI of the Coordinating Group.		
**For non-clinical questions (i.e., unrelated to patient eligibility, treatment, or data submission)** contact the CTSU Help Desk by phone or e-mail:<br>CTSU General Information Line – 1-888-823-5923, or ctsucontact@westat.com. All calls and correspondence will be triaged to the appropriate CTSU representative.		
**For detailed information on the regulatory and monitoring procedures for CTSU sites** please review the CTSU Regulatory and Monitoring Procedures policy located on the CTSU members' website https://www.ctsu.org > education and resources tab > CTSU Operations Information >CTSU Regulatory and Monitoring Policy		
**The CTSU Web site is located at** https://www.ctsu.org		

Schema

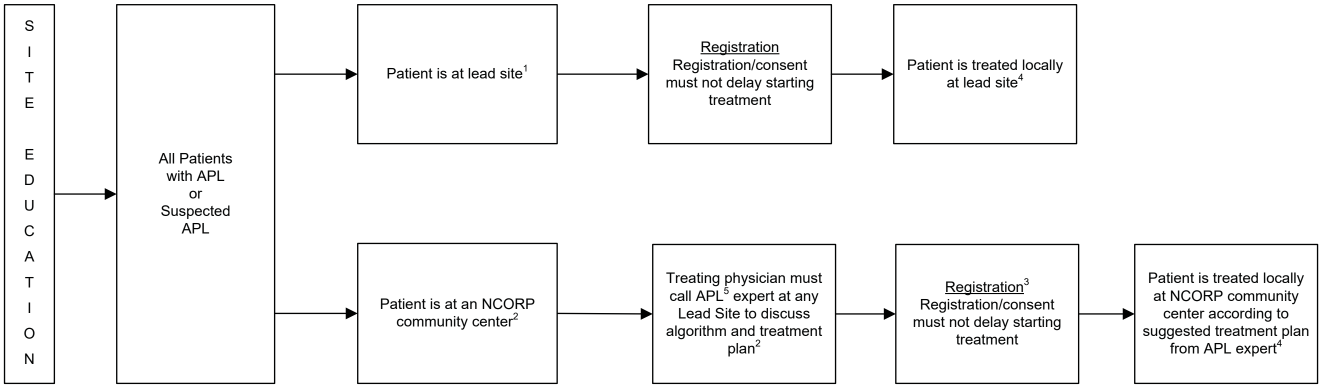

Total Accrual Goal= 200

1. Participation at lead sites will be limited to 30% of overall accrual. Lead sites are identified as: Medical College of George at Augusta University, MSKCC, Mayo Clinic - Rochester, MN, Northwestern, University of Pennsylvania, Mayo Clinic - Jacksonville, FL.
2. It is preferred the APL expert for the catchment area is contacted first. It is suggested that the call be placed as soon as a diagnosis is suspected.
3. Registration is done only if treating physician calls expert within 3 days of starting APL directed treatment.
4. Follow treatment guidelines according to Appendix I. If necessary, deviations from guidelines may occur per discretion of treating physician and consulting physician.
5. 7 investigators at the lead sites have been identified as APL experts.

1. Introduction

1.1 Rationale for Proposed Study

Acute Promyelocytic Leukemia (APL) is a highly curable malignancy with most large cooperative group trials showing a survival above 90%. Patients entered in cooperative group trials are a highly selected group of patients and may not be a representation of outcomes in the general population. It is also likely that patients may not even be seen at a large treatment center due to the advanced nature of the disease and/or socioeconomic and distance constraints. However in recent years, data from single institutions as well as large population data bases show that approximately 1 in 3 patients diagnosed with APL die within the first month of diagnosis.(1-5) The first group to systematically analyze this is a population-based study from Sweden. All patients diagnosed with APL between 1997 and 2006 in the Swedish Adult Leukemia Registry were included in the study. A total of 105 patients with APL were diagnosed during that time and thirty (29%) of these patients died within 30 days of diagnosis with 23 of these 30 patients dying within the first week of diagnosis. The major cause of death was reported as bleeding, with differentiation syndrome, sepsis and multi-organ failure being other causes contributing to early mortality.(1)

While high early mortality was suspected in the US, definite evidence was established only recently by a well-designed epidemiologic study conducted by Park et al by utilizing the Surveillance, Epidemiology and End Results (SEER) program.(2) A total of 1,400 patients diagnosed with APL between 1992 and 2000 were identified. Data from 13 population based cancer registries that participate in the National Cancer Institute's SEER program were analyzed. The overall early death rate of all patients diagnosed with APL was 17.3% and 24% in patients aged > 55 years. The authors from the SEER study also concluded that this might be an underestimate due to the flaws in the SEER reporting system and the actual death rate may be higher than what the study showed. MD Anderson Cancer Center also reported their data from various time periods and compared them with SEER data.(3) From 2002-2008, the 5 year relative survival in APL patients was 64% which is contrary to the reported outcomes in clinical trials. This data also suggests that the lower survival observed in the general population may not be due to deaths in only smaller treatment centers but may also be a problem in larger metropolitan areas (Figure 1). Single institution experience from Stanford University showed an early death rate of 26%, suggesting that this problem might be pervasive even in larger treatment centers.(5)

Figure 1: Early Deaths in APL (Day 1 to 30)

Study	Total patients	Patients died	Mortality rate	Percentage of patients with hemorrhage in early death
**Brazil (2007)[4]**	134	43	32%	66%
**Swedish registry (2011)[1]**	105	30	29%	41%
**SEER data (2011)[2]**	1400	238	17% (24% in > 55yr)	Not discussed
**Stanford (2012)[5]**	70	19	26%	54%
**Ankara, Turkey, (2010)[8]**	49	20	40%	65%
**AIIMS, India (2011)[6]**	33	6	18.8%	Not discussed
**Germany > 60 years (2013)[9]**	91	24	26%	Not discussed
**Hiroshima, Japan > 65 years (2013)[7]**	32	7	21.3%	Not discussed
**GRU (our center) ASCO (2012)[10]**	19	7	37%	57%

Georgia Regents University Experience and Pilot Project:

At Georgia Regents University (GRU), Augusta, GA, from July 2005 to June 2009, 19 patients with APL were treated. Seven of these 19 (37%) patients died during induction due to the following reasons; however the 11 surviving patients are in molecular remission and are presumed cured with a median follow up of 2358 days.

1. Delay in diagnosis and transfer to a treating center
2. Bleeding from coagulopathy
3. Differentiation syndrome
4. Infections
5. Inconsistency in the treatment algorithm and physician inexperience.

After a thorough review, the investigators at GRU formulated a strategy to decrease early deaths and implemented it in 2010. This strategy aided physicians to rapidly diagnose the condition when suspected, and also follow a mechanism to rapidly transfer the patient from rural areas to GRU. A 1.5 page concise treatment algorithm to simplify the treatment process was developed and implemented at GRU. The algorithm was made available to physicians at outlying smaller treatment centers as well. In addition, investigators at GRU communicated with treating physicians in the community and offered guidance.

We treated or co-managed 143 patients thus far using this model, with 10 deaths (6.9%) Furthermore, after implementing the algorithm, data showed an encouraging decrease in induction mortality from an estimated 37% (N=19) to 6.9% (N=143) (Figure 2). Interestingly 40% of patients were treated in experienced leukemia centers and 60% were treated in outlying centers. Patients who survive the first 30 days have an excellent quality of life with a low chance of relapse and treatment related complications.

Figure 2: Survival Pre and Post Algorithm

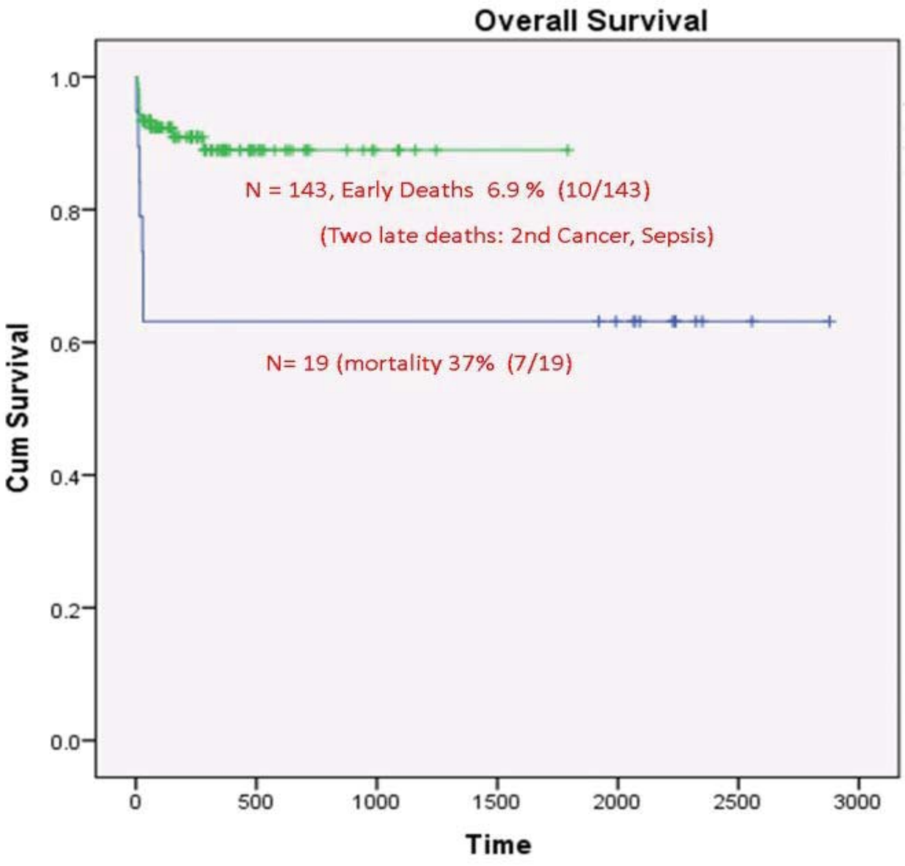

Ongoing Study:

Funding from the Leukemia & Lymphoma Society (LLS) supports the ongoing study efforts to follow the strategy developed at GRU as a model to implement it in the states of Georgia and South Carolina to cover a catchment population of 15 million; the goal is to reduce the induction mortality from an estimated 30% to under 15%.

1.2 Significance of the Study

As discussed earlier, the early mortality in APL continues to be high. The first large scale intervention to decrease early deaths in APL was undertaken by Rego et.al in Brazil, Mexico and Uruguay. In a report published in 2007, it was noted that in Brazil the mortality rate during induction in a cohort of 134 patients was 32%.[(4)] The cumulative mortality during induction and consolidation was 44.7%. The overall survival in this group was 52% which is significantly lower than what is expected in developed countries (62 to 86%). It was obvious that

decreasing induction mortality would make the most impact in improving overall survival in this patient population. Analysis of the causes of early death showed that delayed diagnosis, hemorrhage, differentiation syndrome and infection were major causes of death. This led to the development and implementation of a strategy that addressed all the above mentioned causes of early mortality. This effort was supported by the International Members Committee of the American Society of Hematology (ASH) in 2005. A simple and uniform protocol was developed and launched in Brazil, Mexico, Chile and Uruguay. A standard program for rapid diagnosis, early initiation of treatment, aggressive management of coagulopathy, expedited transfer to a larger center, management of differentiation syndrome and effective treatment of infection was implemented. From 2006 to 2010, 183 patients were enrolled; 122 in Brazil, 30 in Mexico, 23 in Chile and 8 in Uruguay. The cases were individually discussed by a core group of experts with the treating hematologists in outlying hospitals via Internet. One third of the patients were high risk and 52% of the patients were intermediate risk. The induction mortality was 15% a decrease from 32% prior to the implementation of this study. The major cause of early deaths was still hemorrhage (48% of deaths). In addition, the overall survival (OS) improved from 52% to 80% at 2 year follow-up.[(11)]

APL is an uncommon disease; however excellent treatment modalities are available with cure rates of 90%. Current trials that are changing the treatment schedules, adding new drugs, or withholding maintenance might only have a minimal impact on survival in the general population. The intervention that will increase population wide survival is decreasing early deaths. Treating oncologists, who are not routinely involved in the care of acute leukemia patients in smaller centers as well as in larger treatment centers, may not be familiar with the treatment or expected complications. Addressing these issues by educating these physicians and co-managing patients will help improve survival in a condition that has a very high chance of cure.

Patients that were managed using the simple algorithm (Appendix I) demonstrated remarkable improvement in the overall survival. More importantly, a network was established with other leukemia treatment centers in the catchment area and helped local oncologists manage patients and decreased the mortality to 6.9% in 143 patients managed thus far. By replicating these results across a much wider population under the ECOG-ACRIN umbrella with additional sites, it is expected that adherence to the treatment guidelines and consultation with APL experts will reduce mortality rates even more than what previous trials have shown.

Seven of the ten deaths in our experience was in elderly population and although this is significantly better than published literature that suggests non clinical trial deaths to be as high as 50-60% in the same age groups, it shows that patients above the age of 60 years need a different approach. In our approach which is similar to the published literature from Chinese experience, using lower doses or single agent ATRA might lead to better outcomes even in this high risk population.

If the study is positive, efforts will be made to implement the concept nationally and manage APL patients with use of the streamlined guidelines and expert support. A complement of experts is required to be made available permanently and the model should be followed for this project to be sustainable.

1.3 Study Overview

This is a multi-center study with 7 lead investigators accruing at 6 lead sites: Medical College of Georgia at Augusta University, Augusta, GA; Memorial Sloan Kettering Cancer Center, New York, NY; Mayo Clinic, Rochester, MN; Northwestern University, Chicago, IL; University of Pennsylvania, Philadelphia, PA and Mayo Clinic, Jacksonville, FL. The study will be restricted to the above mentioned **FIVE NCTN SITES.** All six sites will be referred to as lead sites**. Other Academic sites will not participate in this trial.**

Physicians treating patients at NCORP community centers will call one of the lead investigators as early as possible but no later than3 days after APL directed treatment is started. The algorithm will be made available and the contacted lead investigator will provide guidance during induction and follow up treatment. The providing physicians will be encouraged to call as and when there are problems.

1.3.1 Treatment guidelines:

The treatment guidelines were developed after extensive review of the literature on standard treatment of APL. Physicians at the NCORP community centers will be provided this algorithm. The study does not exclude patients by age or comorbidities and complete adherence might not be feasible in certain clinical circumstances. This study is directed at co-managing patients by discussions between APL experts and treating physicians to improve the care.

1.3.2 Feasibility

The incidence of APL in the US is approximately 1,500 per year. Our goal is to target an accrual of 200 patients over a 4 year period. As this will be a data collection study, we expect to enroll these patients in this time frame. We do not plan to change the treatment from present standard of care and therefore will not include any new interventions. As described earlier, decreasing early mortality in this curable malignancy will have the biggest impact in improving outcomes. APL is a rare disease and physicians in some centers may not see enough APL patients to be proficient enough. Transferring these patients to larger academic centers is not always feasible for multiple reasons. As an increasing number of community hospitals are treating patients with acute leukemia, it is important to improve the survival in inexperienced centers as well. This would also make it easier for the patient and family and help them remain closer to home. From our experience in rural Georgia and South Carolina, most leukemia treatment centers have adequate supportive care infrastructure such as pharmacy, blood bank, pathology and sub-specialty services. We believe that expert guidance will ensure better adherence to the guidelines and earlier recognition of impending complications and thereby improve survival.

2. Objectives

2.1 Primary Objective

2.1.1 To evaluate if the proposed patient care strategy, that includes use of simplified Guidelines along with APL expert support, decreases the one-month induction mortality rate from 30% to under 15%.

2.2 Secondary Objectives

2.2.1 To assess the overall survival 1 year after accrual is completed.

2.2.2 To assess incidence and severity of differentiation syndrome

2.2.3 To correlate outcomes with time to initiation of ATRA from diagnosis or suspicion of diagnosis.

2.2.4 To evaluate outcomes in academic and community centers separately.

2.2.5 To evaluate factors associated with outcome.

3. Selection of Patients

3.1 Eligibility Criteria

_____ 3.1.1 All patients ≥ 18 years of age who are confirmed to have a diagnosis of APL, which is defined as:

_____ 3.1.1.1 Positive t(15:17) by FISH or conventional karyotype

_____ 3.1.1.2 Positive PML/RAR alpha by PCR

_____ 3.1.2 Patients must accept treatment and supportive care guidelines..

_____ 3.1.3 Referrals must be made as early as possible by treating physician (provider) but no later than 3 calendar days after ATRA or APL directed therapy is initiated. Consent can be obtained up untill day 7 or earlier.

_____ 3.1.4 Co-management can be started as soon as referral is made including weekends. The Physician at the NCORP community facility should make every effort to call the APL expert at the first suspicion of APL.

4. Registration Procedures

CTEP Investigator Registration Procedures

Food and Drug Administration (FDA) regulations and National Cancer Institute (NCI) policy require all investigators participating in any NCI-sponsored clinical trial to register and to renew their registration annually.

Registration requires the submission of:

- a completed ***Statement of Investigator Form*** (FDA Form 1572) with an original signature
- a current Curriculum Vitae (CV)
- a completed and signed ***Supplemental Investigator Data Form*** (IDF)
- a completed ***Financial Disclosure Form*** (FDF) with an original signature

Fillabe PDF forms and additional information can be found on the CTEP website at <http://ctep.cancer.gov/investigatorResources/investigator_registration.htm>. For questions, please contact the ***CTEP Investigator Registration Help Desk*** by email at <pmbregpend@ctep.nci.nih.gov>.

CTEP Associate Registration Procedures / CTEP-IAM Account

The Cancer Therapy Evaluation Program (CTEP) Identity and Access Management (IAM) application is a web-based application intended for use by both Investigators (i.e., all physicians involved in the conduct of NCI-sponsored clinical trials) and Associates (i.e., all staff involved in the conduct of NCI-sponsored clinical trials).

Associates will use the CTEP-IAM application to register (both initial registration and annual re-registration) with CTEP and to obtain a user account.

Investigators will use the CTEP-IAM application to obtain a user account only. (See CTEP Investigator Registration Procedures above for information on registering with CTEP as an Investigator, which must be completed before a CTEP-IAM account can be requested.)

An active CTEP-IAM user account will be needed to access all CTEP and CTSU (Cancer Trials Support Unit) websites and applications, including the CTSU members' website.

Additional information can be found on the CTEP website at <http://ctep.cancer.gov/branches/pmb/associate_registration.htm>. For questions, please contact the ***CTEP Associate Registration Help Desk*** by email at <ctepreghelp@ctep.nci.nih.gov>.

CTSU Registration Procedures

This study is supported by the NCI Cancer Trials Support Unit (CTSU).

IRB Approval:

Each investigator or group of investigators at a clinical site must obtain IRB approval for this protocol and submit IRB approval and supporting documentation to the CTSU Regulatory Office before they can be approved to enroll patients. Study centers can check the status of their registration packets by querying the Regulatory Support System (RSS) site registration status page of the CTSU members' website by entering

credentials at https://www.ctsu.org. For sites under the CIRB initiative, IRB data will automatically load to RSS.

Downloading Site Registration Documents:

Site registration forms may be downloaded from the **EA9131**protocol page located on the CTSU members' website. Add if a restricted access protocol: Permission to view and download this protocol and its supporting documents is restricted and is based on person and site roster assignment housed in the CTSU RSS.

- Go to https://www.ctsu.org and log in to the members' area using your CTEP-IAM username and password
- Click on the Protocols tab in the upper left of your screen
- Click on the **ECOG-ACRIN** link to expand, then select trial protocol **EA9131**
- Click on the Site Registration Documents link

Requirements for EA9131 Site Registration:

- CTSU IRB Certification
- CTSU IRB/Regulatory Approval Transmittal Sheet (for sites not participating via the NCI CIRB)

Submitting Regulatory Documents

Submit completed forms along with a copy of your IRB Approval and Model Informed Consent to the CTSU Regulatory Office, where they will be entered and tracked in the CTSU RSS.

CTSU Regulatory Office
1818 Market Street, Suite 1100
Philadelphia, PA 19103
Phone: 1-866-651-2878
FAX: (215) 569-0206
E-mail: CTSURegulatory@ctsu.coccg.org (for regulatory document submission only)

Required Protocol Specific Regulatory Documents

1. CTSU Regulatory Transmittal Form.
2. Copy of IRB Informed Consent Document.

NOTE: At the main sites, the PI and other designated investigators will obtain the consent.

If a patient is being treated at an NCORP community site, the local PI or Co/Sub-PI will consent the patient. The treating physician at the NCORP community site will contact an APL expert as soon as the diagnosis is suspected but no later than 3 days after ATRA has been initiated.

NOTE: Any deletion or substantive modification of information concerning risks or alternative procedures contained in the sample informed consent document must be justified in writing by the investigator and approved by the IRB.

3. A. CTSU IRB Certification Form.
   Or
   B. Signed HHS OMB No. 0990-0263 (replaces Form 310).
   Or
   C. IRB Approval Letter

   NOTE: The above submissions must include the following details:

   - Indicate all sites approved for the protocol under an assurance number.
   - OHRP assurance number of reviewing IRB
   - Full protocol title and number
   - Version Date
   - Type of review (full board vs. expedited)
   - Date of review.
   - Signature of IRB official

Checking Your Site's Registration Status:

Check the status of your site's registration packets by querying the RSS site registration status page of the members' section of the CTSU website.

NOTE: Sites will not receive formal notification of regulatory approval from the CTSU Regulatory Office.

- Go to https://www.ctsu.org and log in to the members' area using your CTEP-IAM username and password
- Click on the Regulatory tab at the top of your screen
- Click on the Site Registration tab
- Enter your 5-character CTEP Institution Code and click on Go

Patient enrollment will be facilitated using the Oncology Patient Enrollment Network (OPEN). OPEN is a web-based registration system available on a 24/7 basis. To access OPEN, the site user must have an active CTEP-IAM account (check at < https://eapps-ctep.nci.nih.gov/iam/index.jsp >) and a 'Registrar' role on either the LPO or participating organization roster.

The Study Chair Liaison will use OPEN to enroll patients to this study. It is integrated with the CTSU Enterprise System for regulatory and roster data, and upon enrollment, initializes the patient in the Rave database. OPEN can be accessed at https://open.ctsu.org or from the OPEN tab on the CTSU members' side of the website at https://www.ctsu.org.

Patient enrollment for this study will be facilitated using the Slot-Reservation System in conjunction with the Registration system in the Oncology Patient Enrollment Network (OPEN). Prior to discussing protocol entry with the patient, all site staff must contact the Study Chair Liaison, who will use the CTSU OPEN Slot Reservation System to insure that a slot on the protocol is available to the patient. Once a slot- reservation confirmation is obtained, site staff may then proceed to consent the patient to this study. Upon receipt of the consent form, the Study Chair Liaison will then enroll the patient.

Prior to accessing OPEN, site staff should verify the following:

- All eligibility criteria have been met within the protocol stated timeframes.

- All patients have signed an appropriate consent form and HIPAA authorization form (if applicable).

NOTE: The OPEN system will provide the site with a printable confirmation of registration and treatment information. The Study Chair Liaison will print this confirmation and provide it to the site for records.

Further instructional information is provided on the OPEN tab of the CTSU members' side of the CTSU website at https://www.ctsu.org or at https://open.ctsu.org. For any additional questions contact the CTSU Help Desk at 1-888-823-5923 or ctsucontact@westat.com.

4.1 Protocol Number

4.2 Investigator Identification

- Institution and affiliate name
- Investigator's name

4.3 Patient Identification

- Patient's initials (first and last)
- Patient's Hospital ID and/or Social Security number
- Patient demographics
    - Gender
    - Birth date (mm/yyyy)
    - Race
    - Ethnicity
    - Nine-digit ZIP code
    - Method of payment
    - Country of residence

4.4 Eligibility Verification

Patients must meet all of the eligibility requirements listed in Section 3.

4.5 Additional Requirements

4.5.1 Patients must provide a signed and dated, written informed consent form.

NOTE: Copies of the consent are not collected by the ECOG-ACRIN Operations Office

4.5.1.1 Data collection for this study will be done exclusively through the Medidata Rave clinical data management system. Access to the trial in Rave is granted through the iMedidata application to all persons with the appropriate roles assigned in Regulatory Support System (RSS). To access Rave via iMedidata, the site user must have an active CTEP-IAM account (check at < https://eapps-ctep.nci.nih.gov/iam/index.jsp >) and the appropriate Rave role (Rave CRA, Read-Only, Site

Investigator) on either the LPO or participating organization roster at the enrolling site.

Upon initial site registration approval for the study in RSS, all persons with Rave roles assigned on the appropriate roster will be sent a study invitation e-mail from iMedidata. To accept the invitation, site users must log into the Select Login (https://login.imedidata.com/selectlogin) using their CTEP-IAM user name and password, and click on the “accept” link in the upper right-corner of the iMedidata page. Please note, site users will not be able to access the study in Rave until all required Medidata and study specific trainings are completed. Trainings will be in the form of electronic learnings (eLearnings), and can be accessed by clicking on the link in the upper right pane of the iMedidata screen.

Users that have not previously activated their iMedidata/Rave account at the time of initial site registration approval for the study in RSS will also receive a separate invitation from iMedidata to activate their account. Account activation instructions are located on the CTSU website, Rave tab under the Rave resource materials (Medidata Account Activation and Study Invitation Acceptance). Additional information on iMedidata/Rave is available on the CTSU members’ website under the Rave tab at www.ctsu.org/RAVE/ or by contacting the CTSU Help Desk at 1-888-823-5923 or by e-mail at ctsucontact@westat.com.

5. Methodology Plan

There will be 7 investigators accruing at 6 different centers (LEAD SITES) who will serve as the APL experts for this proposal. The patients treated at these 6 centers will be enrolled at their particular institution. **The study will not be open at other academic centers**. The APL expert at each site will either manage the patient or provide consultative oversight to the treating team. The sub-investigators at lead academic sites can consent patients but it is expected that the local PI will provide oversight. We expect to enroll a maximum of 30% of the patients at these academic sites. Induction mortality is a problem in larger centers as well from extensive data that has been published. We postulate that using the guidelines along with expert input will improve outcome in lead sites. This will give us the opportunity to assess the problem as well as outcome in academic centers and compare the results to community centers. Since the accrual goal is 200 patients over a 4 year period, the accrual at lead academic centers will be limited to 15 (30%) patients per year. The Study Chair and ECOG-ACRIN office will monitor this closely and once the target is achieved in a year, enrollment at lead sites will be put on hold. Investigators from the LEAD sites will meet or have a conference call twice a year to discuss the progress and results.

Prior to enrolling patients, while the study is being reviewed by the IRBs at the respective sites, the study investigators will contact NCORP community centers that are likely to treat APL in their catchment area. These visits will also be a part of publicizing the trial to ensure other treating physicians are aware of the study and also the risk of early deaths in this disease. In addition to physician visits, flyers and regular emails will also be sent out to ensure that there are frequent reminders about the study. At sites that open the trial there will also be a webinar to raise awareness for the trial and provide training on the treatment guidelines prior to study activation.

The trial will be conducted at NCORP community sites in addition to the Lead Academic Sites. We conducted a survey of NCORP sites to assess their interest in participating in the study. The survey had 3 questions. a. Does your center treat patients with APL? b. Are you aware the induction mortality in APL is approximately 30%? c. Will your Center participate in a trial focused on decreasing induction mortality? Eleven sites responded and expressed interest. The PI and Co-PI will make personal contact with the other NCORP sites and present the goals of the trial and get other sites to open. We have also asked the NCORP leadership to give us an opportunity to participate in their annual meeting and conference calls and publicize the study to get more sites to participate.

5.1 <u>Lead Sites</u>

5.1.1 Lead Sites

- Medical College of Georgia at Augusta University.
- Memorial Sloan-Kettering Cancer Center, New York, NY
- The Mayo Clinic, Rochester, MN
- Northwestern University Feinberg School of Medicine, Chicago, IL
- Abramson Cancer Center of the University of Pennsylvania, Philadelphia, PA
- The Mayo Clinic, Jacksonville, FL

5.1.2 Patients treated at Lead Sites will be treated locally at the Lead Site using treatment guidelines. The local PI will provide expert support to

the treating team. The presentation of APL can be highly variable; hence adherence to the guidelines is not always feasible. Deviations that are made from the guidelines based on particular clinical situations will be documented in the medical record.

5.2 Outlying NCORP Community Facility

5.2.1 The study will be opened only at the NCORP community sites. **It will not be open at academic NCORP centers.** The treating physician from the NCORP community Facility should call the APL Expert in their catchment area for treatment guidance. Seeking advice from an APL expert is a key component of our model. The call should be made as soon as APL is suspected but no later than 3 days after starting ATRA and communication should be ongoing based on the clinical developments until a hematologic remission is achieved. The presentation in APL can be highly variable; hence complete adherence to the guidelines is not always feasible. Deviations that are made from the guidelines based on particular clinical situations will be documented in the medical record.

5.2.1.1 The provider at NCORP community centers will be the Hematologist/Oncologist attending caring for the patient. Initial contact has to be made by a Hematologist/Oncologist.

5.2.1.2 It is expected that the initial contacting Hematologist/Oncologist will continue the communication; alternatively, other members of the practice can communicate with the APL expert on an ongoing basis.

5.2.1.3 Trainees or advanced level practice providers (physician assistants or nurse practitioners) and trainees can be in communication subsequently with APL experts to continue the care.

5.2.2 A log will be maintained by the APL expert (Please see Appendix II). This will include information on Date admitted, date APL suspected, date APL confirmed, date APL therapy started and date APL expert was contacted. Subsequent calls and decisions made will be documented on the log sheet.

5.2.3 It will be suggested that the treating physician will also document the communication on an ongoing basis.

5.3 Study Accrual and Timeline

The study will accrue from years one to 4. Follow up will be for one year after last patient is accrued and accrual is completed. The total study duration is 5 years.

5.4 Adverse Event Reporting Requirements

The adverse events to treatment will be collected from the hospital records obtained from the patients induction period and also follow up at the treating physician's office. An AE log need not be maintained since this study will not include any new drugs. The complications from treatment will be noted in the database and will be used for analysis of outcomes. The only SAE that has to be reported is death.

5.4.1 Reporting Procedure

This study requires that expedited serious adverse event reporting use CTEP's Adverse Event Reporting System (CTEP-AERS). The CTEP's guidelines for CTEP-AERS can be found at http://ctep.cancer.gov. A CTEP-AERS report must be submitted electronically to ECOG-ACRIN and the appropriate regulatory agencies via the CTEP-AERS Web-based application located at http://ctep.cancer.gov.

In the rare event when Internet connectivity is disrupted a 24-hour notification is to be made by telephone to

- the SAE Team at ECOG-ACRIN (617-632-3610)
- the NCI (301-897-7497)

An electronic report MUST be submitted immediately upon re-establishment of internet connection.

Supporting and follow up data: Any supporting or follow up documentation must be uploaded to the Supplemental Data Folder in Medidata Rave within 48-72 hours. In addition, supporting or follow up documentation must be faxed to the NCI (301- 230-0159) in the same timeframe.

CTEP Technical Help Desk: For any technical questions or system problems regarding the use of the CTEP-AERS application, please contact the NCI Technical Help Desk at ncictephelp@ctep.nci.nih.gov or by phone at 1-888-283-7457.

5.4.2 Expedited Reporting Requirements for EA9131

**Grade 5**[a]	
Unexpected	Expected
24-Hour 5 Calendar Days	24-Hour 5 Calendar Days
24-Hour 5 Calendar Days	24-Hour 5 Calendar Days
**24-Hour; 5 Calendar Days** – The AE must initially be reported via CTEP-AERS within 24 hours of learning of the AE, followed by a complete expedited report within 5 calendar days of the initial 24-hour report.	
[a] This includes all deaths within one year of registration, regardless of cause. Thereafter, deaths must only be reported on the protocol specific case report forms.	

5.4.3 Other recipients of adverse event reports and supplemental data

ECOG-ACRIN will forward CTEP-AERS reports to the appropriate regulatory agencies and pharmaceutical company, if applicable.

Serious Adverse events determined to be reportable via CTEP-AERS must also be reported by the institution, according to the local policy and procedures, to the Institutional Review Board responsible for oversight of the patient.

5.5 Completion of Induction

Induction will be continued until the patient achieves hematologic remission (as per guidelines).

5.6 Consolidation

Consolidation will be at the treating physician's discretion.

5.7 Maintenance

Maintenance will be per treating physician's discretion.

5.8 Duration of Follow-up

For this protocol, all patients, including those who discontinue treatment early, will be followed for survival for 1 year after the accrual is completed.

6. Study Parameters

NOTE: Please refer to Appendix I for all recommended tests and procedures

	**Baseline**
Confirmed APL Dianosis[1]	X
Referral to APL Expert[2]	X
Informed Consent[3]	X
Subject Registration[4]	X

1. Positive t(15:17) by FISH or conventional karyotype and PML/RARα by PCR, rare variants can also be included if confirmed.
2. It is preferred that the APL expert for the catchment area is contacted, however, any APL Expert at any Lead Site may be contacted. Referrals must be made within 3 calendar days after ATRA or APL directed therapy is initiated.
3. Written informed consent must be obtained within 7 days of confirmed diagnosis.
4. Registration can be done after initiating treatment.

7. Statistical Considerations

7.1 Study Design and Objectives

This study will accrue 200 APL patients over a 4-year period. The primary hypothesis is that the proposed process will lower the one-month mortality rate from 30% to < 15%. The primary endpoint is the one-month mortality rate Secondary endpoints include: i) overall survival, one-year survival rate, ii) safety, iii) initiation of treatment from diagnosis, iv) survival in academic and community centers v) compliance with treatment guidelines and vi) evaluation of factors associated with outcome.

7.2 Sample Size Considerations and Monitoring Plan

Of the 200 patients accrued, it is expected to have 180 eligible patients after adjusting for ineligible and/or lost-to-follow up cases. We allowed 10% for ineligible and/or lost-to-follow-up cases. We often allow additional cases (up to 10%) to account for ineligible cases in ECOG studies. We do not expect any lost-to-fu cases at 1 month. However, we used 10% ineligibility and/or lost-to-follow-up to be conservative and general in this study.

After 100 patients (for 90 eligible) are enrolled and have followed for one-month mortality endpoint, one interim analysis will be conducted. Using the critical value corresponding to the O'Brien and Fleming boundary at 50% information time, 95% Repeated Confidence Interval (RCI) for the one-month mortality rate will be constructed. The result will be discussed with the ECOG-ACRIN DSMC members. If deemed efficacious, accrual will continue to 200 (for 180 eligible) patients. This design will have at least 90% power with a two-sided type I error rate of < 0.05. Another rationale for the sample size of 200 is that we would like to build a 95% RCI with a tight interval. For the expected one-month mortality rate, if the observed rate is 10% (18/180) the 95% RCI will be 6-15.4%.If the observed one-month mortality rate is 15%, the 95% RCI will be 10.1-21.1%.

We plan to limit the accrual from the large academic centers to a maximum of 30% of the total patients. Thus 60 patients (54 eligible) from major academic centers and 140 (126 eligible) from NCORP community sites are anticipated. The primary analysis and monitoring will be based on all patients regardless of enrollment site. After the study is completed, one-month mortality rate will be estimated for patients enrolled from NCORP and academic sites separately as a secondary analysis. With 126 eligible patients from NCORP sites, there will be at least 90% power for the hypothesis testing of reducing mortality rate from 30% to 15%, with a two-sided error rate of 0.05. There is sufficient power to test if one-month mortality rate for NCORP sites can be reduced from 30% to < 15%

For NCORP sites, 95% CI is 5.6-17% for the observed one-month mortality rate of 10.3% (13/126) and 95% CI is 9.3-22.5% for the observed one-month mortality rate of 15.1%. (19/126). With 54 eligible patients from academic sites, there will be less than 80% power for the hypothesis testing of reducing mortality rate from 30% to 15%, with a two-sided error rate of 0.05. For academic sites, 95% CI is 3-20.3%% for the observed one-month mortality rate of 9.3% (5/54) and 95% CI is 6.6-27.1% for the observed one-month mortality rate of 14.8%. (8/54). Statistical analysis will be mostly descriptive for the secondary endpoints.

At 50% information time (i.e. 90 eligible patients have one-month mortality assessed), 95% Repeated Confidence Interval will be built for the one-month mortality estimate using the O'Brien-Fleming boundary for 50% information time. This analysis will be presented to the ECOG-ACRIN DSMC and discussed further.

We do not expect major safety issues in this study. However adverse events data will be monitored in ongoing basis and will be brought to ECOG-ACRIN DSMC attention.

7.3 Statistical Analysis Plan

7.3.1 Primary Objective

One-month mortality rate will be estimated by dividing total number of deaths within one month of study entry by total number of cases with at least one-month follow up data. 95% RCI will be provided for this endpoint. We will also evaluate the OS data using the Kaplan-Meier plot.

7.3.2 Secondary Objectives

Using the method of Kaplan-Meier, overall survival (OS) data will be described. The median OS and one-year OS rate will be estimated and 95% CI will be provided.

We confirm that the primary objective is to assess one-month mortality rate among all cases. Secondary analysis will involve evaluating outcome data in NCORP and academic sites separately. We clarified this further in Section 7.2. Overall the expected one-month mortality rate is in the range of 10-15% across all patients as well as in two subgroups of NCORP and academic sites.

With 200 (180 eligible) patients, we expect to build a confidence interval not wider than 11% for the expected one-month mortality rate of 10-15%. With 140 (126 eligible) cases from NCORP sites, the confidence interval for the one-month mortality rate will be not be wider than 13%.

For the safety endpoints, the severity and duration of coagulopathy, bleeding, infections and differentiation syndrome will be measured based on the CTC version 4.0. Analysis of the safety data will be mostly descriptive. The proportion of patients with the worst degree toxicity of grade 3 or higher will be assessed and presented with 95% CI. The length of hospital stay is defined as a period between the discharge and admission dates for the first hospitalization. For patients with no hospitalization, this will be defined as 0. To assess the associations between the length of hospital stay and toxicity measures (bleeding, infections, differentiation syndrome), each toxicity measures will be dichotomized as "mild" vs. "severe" and the length of hospital stay will be compared using the two-sample t-test.

The time from diagnosis and initiation of treatment will be assessed and dichotomized as "short' vs. "long" using the median value. One-month mortality rate will be compared in these two groups (short vs. long) using the Fisher's exact test. Overall-survival will also be

compared in these two groups using the logrank test. In this analysis, the time of diagnosis will be determined by both: i) confirmed diagnosis date and ii) date of suspicious APL. The dichotomized value of long vs. short based on the both times will be evaluated. Similarly the one-month morality rate and overall survival between the lead institutions and NCORP community centers will be compared. For all comparisons, two-sided p-values will be reported.

Compliance with treatment guidelines will be assessed as binary outcome and the successful compliance rate will be described with 95% confidence interval. A regression model will be developed to evaluate the factors associated with outcome. Data items such as age, risk status, comorbidity will be collected and evaluated.

8. Electronic Data Capture

Please refer to the **EA9131** Forms Completion Guidelines for the forms submission schedule. Data collection will be performed exclusively in Medidata Rave. In case of withdrawal from the study, data will be collected to the point of withdrawal.

9. Patient Consent and Peer Judgment

Current FDA, NCI, state, federal and institutional regulations concerning informed consent will be followed.

10. References

1. Lehmann S, Ravn A, Carlsson L, Antunovic P, Deneberg S, Mollgard L, et al. Continuing high early death rate in acute promyelocytic leukemia: a population-based report from the Swedish Adult Acute Leukemia Registry. Leukemia. 2011;25(7):1128-34.
2. Park JH, Qiao B, Panageas KS, Schymura MJ, Jurcic JG, Rosenblat TL, et al. Early death rate in acute promyelocytic leukemia remains high despite all-trans retinoic acid. Blood. 2011;118(5):1248-54.
3. Chen Y, Kantarjian H, Wang H, Cortes J, Ravandi F. Acute promyelocytic leukemia: a population-based study on incidence and survival in the United States, 1975-2008. Cancer. 2012;118(23):5811-8.
4. Jacomo RH, Melo RA, Souto FR, de Mattos ER, de Oliveira CT, Fagundes EM, et al. Clinical features and outcomes of 134 Brazilians with acute promyelocytic leukemia who received ATRA and anthracyclines. Haematologica. 2007;92(10):1431-2.
5. McClellan JS, Kohrt HE, Coutre S, Gotlib JR, Majeti R, , et al.Treatment advances have not improved the early death rate in acute promyelocytic leukemia. Haematologica.2012;97(1):133-6.
6. Bajpai J1, Sharma A, Kumar L, Dabkara D, Raina V, Kochupillai V, Kumar R. Acute promyelocytic leukemia: an experience from a tertiary care centre in north India. Indian J Cancer. 2011 Jul-Sep;48(3):316-22.
7. Imagawa J, Harada Y, Shimomura T, Tanaka H, Okikawa Y, Harada H. High early death rate in elderly patients with acute promyelocyticleukemia treated with all-trans retinoic acid combined chemotherapy. Int J Hematol. 2013 Aug;98(2):264-6.
8. Serefhanoglu, S., et al. (2010). "Clinical features and outcomes of 49 Turkish patients with acute promyelocytic leukemia who received ATRA and anthracyclines (PETHEMA protocol) therapy." Leuk Res 34(12): e317-319
9. Lengfelder, E., et al. (2013). "Outcome of elderly patients with acute promyelocytic leukemia: results of the German Acute Myeloid Leukemia Cooperative Group." Ann Hematol 92(1): 41-52
10. Anand P. Jillella, Ibrahim Sadek, Devi Morrison, Kavita Natrajan, Farrukh Tauseef Awan, Ravindra Kolhe, Vamsi Kota. A simple but effective model to decrease early deaths in acute promyelocytic leukemia (APL). J Clin Oncol 30, 2012 (suppl; abstr 6573)
11. Rego EM, Kim HT, Ruiz-Arguelles GJ, Undurraga MS, Uriarte Mdel R, Jacomo RH, et al. Improving acute promyelocytic leukemia (APL) outcome in developing countries through networking, results of the International Consortium on APL. Blood. 2013;121(11):1935-43.

A Simplified Patient Care Strategy to Decrease Early Deaths in Acute Promyelocytic Leukemia (APL)

Appendix I

Acute Promyelocytic Leukemia Treatment Guidelines

Developed at Georgia Regents University, August 2013 Revised May 2017

AUTHORS: Dr. Anand P. Jillella Mobile: 706 -951- 5144 Office: 706-721-2505
Dr. Vamsi K. Kota Mobile: 706- 825- 4091 Office: 404-778-1900

WORK-UP

BASELINE

- Chest X-ray
- Echocardiogram and EKG
- PICC line placement – NO central lines in chest or neck or invasive procedures (bronchoscopy, spinal tap, endoscopy)
- Bone marrow examination (aspirate, biopsy, flow, cytogenetics, FISH for t15:17, PCR for PML-RARa). Day 14 marrow is **NOT** necessary

DAILY

- CBC, CMP, PT, PTT, fibrinogen **TWICE DAILY** until lab & clinical coagulopathy is resolved. CMP daily and twice a day as dictated by clinical condition.
- D-dimer **ONCE DAILY** for **ENTIRE** hospitalization.

PREVENTION/TREATMENT OF COAGULOPATHY

- Intracranial, pulmonary and GI hemorrhages are frequent
- Risk of hemorrhage is ↑ with the presence of any of the following - active sites of bleeding, ↓ fibrinogen, ↑ D-dimers, ↑ PT & PTT, ↑ WBC, ↑peripheral blasts, renal failure and poor performance status;
- **Keep platelets above 50,000**
- If there are any active sites of bleeding on presentation (e.g., needle sticks, bone marrow biopsy sites,), and prolonged PT/PTT), give 4 units of fresh frozen plasma (FFP) at the start of ATRA and chemotherapy. **CONTINUE** FFP support **TWICE A DAY** until clinical bleeding resolves
- **Keep fibrinogen above 150**. Use 10 units of cryoprecipitate if < 150.
- When clinical & lab coagulopathy is resolved, blood product support is per standard guidelines.

⟶ APL IS A MEDICAL EMERGENCY - START ATRA ASAP ⟵

PREVENTION/TREATMENT OF APL DIFFERENTIATION SYNDROME

Differentiation Syndrome-symptoms are – dyspnea, unexplained fever, ↑ weight, peripheral edema, unexplained hypotension, acute renal failure, CHF, pleuro-pericardial effusions, and interstitial pulmonary infiltrates.

- Daily weights - **BEDSIDE SCALES ONLY**
- Keep I/O matched – **METICULOUSLY**

- Diuretics if ↑fluid retention or ↑weight
- Prednisone 0.5 to 1 mg/Kg in all patients for 2 weeks as prophylaxis starting on day 1 followed by a taper
- Increase Dexamethasone to 10 mg IV BID from the prophylactic dose above, if onset of DS symptoms
- If WBC >10,000, dexamethasone 10 mg IV bid tocould be started on day 1before initiating ATRA
- Temporary discontinuation of ATRA or Arsenic Trioxide (ATO) is indicated only in case of severe cases of DS.
- Continue dexamethasone until 3 days after all symptoms are resolved. ATRA and/or arsenic can be resumed.

SUPPORTIVE CARE

- Allopurinol 300 mg daily
- Antibiotic prophylaxis - levofloxacin 500 mg po daily or similar antibiotic
- Antifungal prophylaxis - posaconazole 200 mg po 3 x3x daily, voriconazole 200 mg po 2 x2x daily, Micafungin 50 mg daily or a similar drug as per institutional standard.
- Anti-viral prophylaxis - acyclovir 400 mg po 2 x2x daily or valacyclovir 1000 mg PO daily
- RBC transfusion recommended when HGB ↓ 8.

INDUCTION OF LOW AND INTERMEDIATE RISK PATIENTS WBC < 10,000 and PLTS < 40,000 -

- ATRA 45mg/m2 in divided doses twice a day plus ATO 0.15 kg daily until complete hematologic remission (no anthracycline). Prednisone 0.5 mg/kg for 14 days followed by a gradual taper if no evidence of DS.

  OR
- ATRA on Day 1 at 45 mg/m2 DAILY in divided doses twice a day until complete hematologic remission.
- Idarubicin 12 mg/m2 on Days 2, 4, 6 and 8. Prednisone 0.5 mg/kg for 14 days followed by a gradual taper if no evidence of DS.

INDUCTION OF HIGH RISK PATIENTS > 10,000

- ATRA to be started as soon as diagnosis is suspected at 45 mg/m2 in divided doses twice a day until complete hematologic remission.
- Idarubicin to be started on the SAME DAY OR AS SOON AS POSSIBLE WHEN THE DIAGNOSIS IS CONFIRMED at 12 mg/M2 on days 1, 3, 5 and 7.
- Dexamethasone 10 mg IV bid on days 1 to 14 followed by a taper.
- Arsenic at 0.15 mg/Kg can be started on day 10
- Even if the genetic results are not available, it is reasonable to start Idarubicin if the diagnosis is otherwise strongly suggested.

ARSENIC TRIOXIDE USE IN INDUCTION

CONSIDER ATO

- Age >60
- Not a candidate for conventional chemotherapy for any reason.

- Arsenic at 0.15 mg/kg daily, continued until complete hematologic remission
- Combine with ATRA 45mg/m2 DAILY in divided doses
- Prednisone 0.5 mg/Kg for 14 days and taper from day 1 of therapy; if high risk, dexamethasone 10 mg IB bid on days 1 to 14 followed by a taper.
- Watch for differentiation syndrome
- Watch for prolonged QTc interval
- Keep Mg above 2.0 and K above 4.0
- Follow LFTs for grade 2 - 4 liver dysfunction. If this occurs HOLD arsenic.

SUGGESTED MANAGEMENT OF ATRA/ARSENIC-INDUCED LEUKOCYTOSIS

- WBC 5 - 50 K- Hydroxyurea 500 mg qid
- WBC > 50K- Hydroxyurea 1000 mg qid
- If leukocytosis does not resolve one or two doses of Idarubicin could be considered
- **NO LEUKOPHERESIS.**

ELDERLY PATIENTS AND PATIENTS WITH SEVERE COMORBID CONDITIONS

- May not be candidates for cytotoxic chemotherapy
- Consider dose reduction of ATRA. Single agent and consider 25 mg/M2 for 14 days.

Consider dose reduction of ATO and consider 0.075 mg/M2 and add on day 15

A Simplified Patient Care Strategy to Decrease Early Deaths in Acute Promyelocytic Leukemia (APL)

Appendix II

APL Expert and NCORP Provider Communication

1. Date patient admitted -
2. Date APL suspected -
3. Date APL confirmed -
4. Date APL therapy started -
5. Date APL expert contacted -
6. Subsequent communications -

Date:

Date:

Date:

Date:

Date:

Date:

Date:

Date:

Date:

Date:

A Simplified Patient Care Strategy to Decrease Early Deaths in Acute Promyelocytic Leukemia (APL)

Appendix III

NCORP PROVIDER INFORMATION SHEET

This is a research study focused on decreasing induction mortality in Acute Promyelocytic Leukemia (APL). Historical data shows an induction mortality of 30% in the general population and the purpose of this study is to decrease it to < 15%. NCORP providers will be given this information sheet along with the treatment guidelines in Appendix I.

A. The study will consist of using the simplified APL treatment algorithm that is attached.

B. Equally important is for the provider at NCORP sites to call an APL expert listed in the protocol or the PI/Co-PI. Cell phone numbers of APL experts are in the Protocol.

C. The APL expert will function as a resource during induction to prevent and manage complications. It is suggested that the provider at NCORP sites call the APL expert as frequently as needed during the first 4 weeks. The experts are available 24/7.

D. NCORP providers will document discussion with the APL expert and clinical decisions in the Medical Record. APL experts will fill the Expert/NCORP Provider communication sheet.

E. There are no obvious discomforts to the patient since the patient will be treated on the accepted standard of care. Expert support may help in preventing and managing complications and improving outcome.

F We will retain all patient records and signed informed consent forms filed in a secure location. Database is maintained using web-based database system. Only research members are granted access to web-based database system with user ID and password.

G. Participation is voluntary and the patient may choose not to participate in the trial. In that case the patient will be treated per usual treatment guidelines. Participants may discontinue at any time without penalty or loss of benefits.

A Simplified Patient Care Strategy to Decrease Early Deaths in Acute Promyelocytic Leukemia (APL)

Appendix IV

Patient Thank You Letter

We ask that the physician use the template contained in this appendix to prepare a letter thanking the patient for enrolling in this trial. The template is intended as a guide and can be downloaded from the web site at http://www.ecog.org. As this is a personal letter, physicians may elect to further tailor the text to their situation.

This small gesture is a part of a broader program being undertaken by ECOG-ACRIN and the NCI to increase awareness of the importance of clinical trials and improve accrual and follow-through. We appreciate your help in this effort.

[PATIENT NAME] [DATE]

[PATIENT ADDRESS]

Dear Mr. / Mrs. / Ms. ___________,

Thank you for agreeing to take part in this important research study. Many questions remain unanswered in cancer. With the participation of people like you in clinical trials, we will improve treatment and quality of life for those with your type of cancer.

We believe you will receive high quality, complete care. I and my research staff will maintain very close contact with you. This will allow me to provide you with the best care while learning as much as possible to help you and other patients.

On behalf of ___________________ and ECOG-ACRIN, we thank you again and look forward to helping you.

Sincerely,

[PHYSICIAN NAME]

EA9131

A Simplified Patient Care Strategy to Decrease Early Deaths in Acute Promyelocytic Leukemia (APL)

STUDY CHAIR: Anand P. Jillella, MD
STUDY CO-CHAIR: Vamsi K. Kota, MD
STUDY STATISTICIAN: Sandra Lee, ScD
PREVENTION COMMITTEE CHAIR: Raymond Bergan, MD
CANCER CARE DELIVERY RESEARCH COMMITTEE CHAIR: Ruth Carlos, MD
Rev. Add2 CANCER CONTROL & SURVIVORSHIP COMMITTEE CHAIR: Lynne I. Wagner, PhD
LEUKEMIA COMMITTEE CHAIR: Mark R. Litzow, MD

Version Date: May 25, 2018

STUDY PARTICIPANTS

ALLIANCE / Alliance for Clinical Trials in Oncology, NCORP Sites Only

ECOG-ACRIN / NCORP Sites Only

NRG / NRG Oncology, NCORP Sites Only

SWOG / SWOG, NCORP Sites Only

COG / Children's Oncology Group, NCORP Sites Only

NCTN participation is limited to the following sites:

FL080 / Mayo Clinic, Jacksonville (ECOG-ACRIN)

MN026 / Mayo Clinic, Rochester (ECOG-ACRIN)

NY016 / Memorial Sloan-Kettering Cancer Center (ECOG-ACRIN)

IL036 / Northwestern University (ECOG-ACRIN)

PA075 / University of Pennsylvania (ECOG-ACRIN)

GA020 / Medical College of Georgia at August University (ECOG-ACRIN)

ACTIVATION DATE

August 16, 2017

Addendum #1 – Prior to Activation

Addendum #2

STUDY CHAIR

Anand P. Jillella, MD
Georgia Cancer Center
Medical College of Georgia at Augusta University
1120 15th Street, BAA 5407
Augusta, GA, 30912
Office Phone: 706-721-2505
Cell Phone: 706-951-5144
Fax: 706-721-5566
Email: ajillella@augusta.edu

STUDY CO-CHAIR

Vamsi K. Kota, MD
Winship Cancer Institute of Emory University
1365 Clifton Road NE, Room C1152
Atlanta, GA 30322
Office Phone: 404-778-1900
Cell Phone: 706-825-4091
Fax: 404-778-4755
Email: vkota@emory.edu

Rev. Add2

STUDY CHAIR LIAISON (SCL)

Prachi Karkhanis
Georgia Cancer Center
Medical College of Georgia at Augusta University
1120 15th Street, BAA 5407
Augusta, GA 30912
Office Phone: 706-721-9491
Fax: 706-721-5566
Email: pkarkhanis@augusta.edu

ECOG-ACRIN STUDY EXPERTS

Anand P. Jillella, MD
Georgia Cancer Center
Medical College of Georgia at Augusta University
1120 15th Street, BAA 5407
Augusta, GA, 30912
Office Phone: 706-721-2505
Cell Phone: 706-951-5144
Fax: 706-721-5566
Email: ajillella@augusta.edu

Jessica K. Altman, MD
Northwestern University Feinberg School of Medicine
Robert H. Lurie Comprehensive Cancer Center
303 E. Superior Street, Chicago, IL 60611
Office Phone: 312-503-1817
Cell Phone: 773-575-2598
Fax: 312-695-6189
Email: j-altman@northwestern.edu

Vamsi K. Kota, MD
Winship Cancer Institute of Emory University
1365 Clifton Road NE, Room C1152
Atlanta, GA 30322
Office Phone: 404-778-1900
Cell Phone: 706-825-4091
Fax: 404-778-4755
Email: vkota@emory.edu

Selina Luger, MD
Abramson Cancer Center of the University of Pennsylvania
2 West, Perelman Center for Advanced Medicine
3400 Civic Center Road, Philadelphia, PA, 19104
Office Phone: 215-662-6348
Cell Phone: 215-279-0657
Fax: 215-662-4064
Email: selina.luger@uphs.upenn.edu

Martin S. Tallman, MD
Memorial Sloan-Kettering Cancer Center
Leukemia Service, Department of Medicine
1275 York Avenue, New York, NY 10065
Office Phone: 212-639-3842
Cell Phone: 646-467-4670
Fax: 212-639-3841
Email: tallmanm@mskcc.org

James M. Foran, MD, FRCPC
Mayo Clinic Florida
Division of Hematology & Medical Oncology
4500 San Pablo Road, Jacksonville, FL 32224
Office Phone: 904-953-7292
Cell Phone: 904-955-3679
Fax: 904-953-2315
Email: foran.james@mayo.edu

Mark R. Litzow, MD
The Mayo Clinic
Division of Hematology
200 First Street, SW, Rochester, MN 55905
Office Phone: 507-266-0523
Cell Phone: 507-269-4848
Fax: 507-266-4972
Email: litzow.mark@mayo.edu

Table of Contents

Schema 6
1. Introduction 7
1.1 Rationale for Proposed Study 7
1.2 Significance of the Study 9
1.3 Study Overview 11
2. Objectives 12
2.1 Primary Objective 12
2.2 Secondary Objectives 12
3. Selection of Patients 13
3.1 Eligibility Criteria 13
4. Registration Procedures 14
4.1 Protocol Number 17
4.2 Investigator Identification 17
4.3 Patient Identification 18
4.4 Eligibility Verification 18
4.5 Additional Requirements 18
5. Methodology Plan 20
5.1 Lead Sites 20
5.2 Outlying NCORP Community Facility 21
5.3 Study Accrual and Timeline 21
5.4 Adverse Event Reporting Requirements 21
5.5 Completion of Induction 23
5.6 Consolidation 23
5.7 Maintenance 23
5.8 Duration of Follow-up 23
6. Study Parameters 24
7. Statistical Considerations 25
7.1 Study Design and Objectives 25
7.2 Sample Size Considerations and Monitoring Plan 25
7.3 Statistical Analysis Plan 26
7.4 Race, Gender, and Ethnicity Accrual Estimates 27
8. Electronic Data Capture 28
9. Patient Consent and Peer Judgment 28
10. References 28
Appendix I Acute Promyelocytic Leukemia Treatment Guidelines 29
Appendix II APL Expert and NCORP Provider Communication 32
Appendix III NCORP PROVIDER INFORMATION SHEET 33
Appendix IV Patient Thank You Letter 34

CANCER TRIALS SUPPORT UNIT (CTSU) ADDRESS AND CONTACT INFORMATION

Rev. Add2

For regulatory requirements:	For patient enrollments:	Submit study data
Regulatory documentation must be submitted to the CTSU via the Regulatory Submission Portal.<br>Regulatory Submission Portal: (Sign in at www.ctsu.org, and select the Regulatory Submission sub-tab under the Regulatory tab.)<br>Institutions with patients waiting that are unable to use the Portal should alert the CTSU Regulatory Office immediately at 1-866-651-2878 to receive further instruction and support.<br>Contact the CTSU Regulatory Help Desk at 1-866-651-2878 for regulatory assistance.	Please refer to the patient enrollment section of the protocol for instructions on using the Oncology Patient Enrollment Network (OPEN) which can be accessed at https://www.ctsu.org/OPEN_SYSTEM/ or https://OPEN.ctsu.org.<br>Contact the CTSU Help Desk with any OPEN-related questions at ctsucontact@westat.com.	Data collection for this study will be done exclusively through Medidata Rave.<br>Please see the data submission section of the protocol for further instructions.
The most current version of the **study protocol and all supporting documents** must be downloaded from the protocol-specific Web page of the CTSU Member Web site located at https://www.ctsu.org. Access to the CTSU members' website is managed through the Cancer Therapy and Evaluation Program - Identity and Access Management (CTEP-IAM) registration system and requires user log on with CTEP-IAM username and password.		
**For clinical questions (i.e., patient eligibility or treatment-related)** Contact the Study PI of the Coordinating Group.		
**For non-clinical questions (i.e., unrelated to patient eligibility, treatment, or data submission)** contact the CTSU Help Desk by phone or e-mail:<br>CTSU General Information Line – 1-888-823-5923, or ctsucontact@westat.com. All calls and correspondence will be triaged to the appropriate CTSU representative.		
**The CTSU Web site is located at** https://www.ctsu.org		

Schema

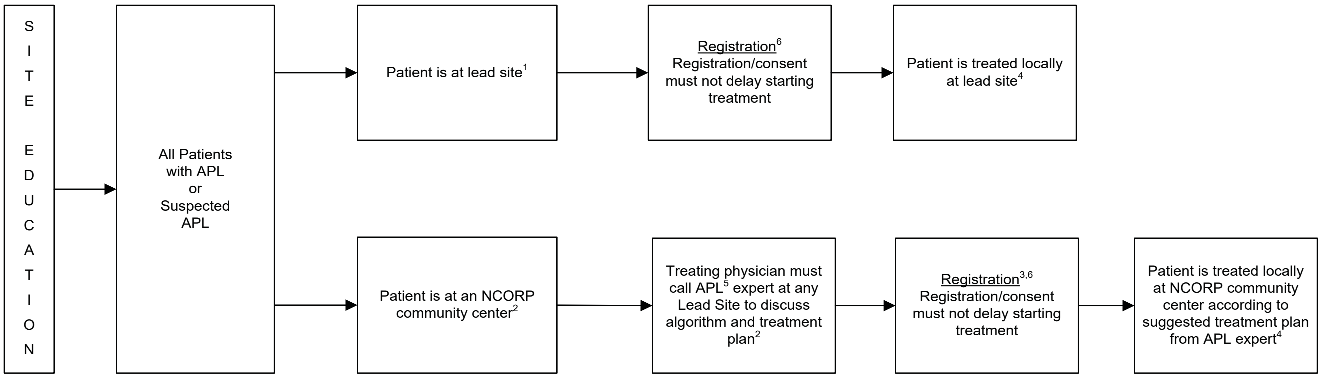

Total Accrual Goal= 200

1. Participation at lead sites will be limited to 30% of overall accrual. Lead sites are identified as: Medical College of George at Augusta University, MSKCC, Mayo Clinic - Rochester, MN, Northwestern, University of Pennsylvania, Mayo Clinic - Jacksonville, FL.
2. It is preferred the APL expert for the catchment area is contacted first. It is suggested that the call be placed as soon as a diagnosis is suspected.
3. Registration is done only if treating physician calls expert within 3 days of starting APL directed treatment.
4. Follow treatment guidelines according to Appendix I. If necessary, deviations from guidelines may occur per discretion of treating physician and consulting physician.
5. 7 investigators at the lead sites have been identified as APL experts.
6. Consent patient after APL diagnosis is confirmed. Rev. Add2

1. Introduction

1.1 Rationale for Proposed Study

Acute Promyelocytic Leukemia (APL) is a highly curable malignancy with most large cooperative group trials showing a survival above 90%. Patients entered in cooperative group trials are a highly selected group of patients and may not be a representation of outcomes in the general population. It is also likely that patients may not even be seen at a large treatment center due to the advanced nature of the disease and/or socioeconomic and distance constraints. However in recent years, data from single institutions as well as large population data bases show that approximately 1 in 3 patients diagnosed with APL die within the first month of diagnosis.[1-5] The first group to systematically analyze this is a population-based study from Sweden. All patients diagnosed with APL between 1997 and 2006 in the Swedish Adult Leukemia Registry were included in the study. A total of 105 patients with APL were diagnosed during that time and thirty (29%) of these patients died within 30 days of diagnosis with 23 of these 30 patients dying within the first week of diagnosis. The major cause of death was reported as bleeding, with differentiation syndrome, sepsis and multi-organ failure being other causes contributing to early mortality.[1]

While high early mortality was suspected in the US, definite evidence was established only recently by a well-designed epidemiologic study conducted by Park et al by utilizing the Surveillance, Epidemiology and End Results (SEER) program.[2] A total of 1,400 patients diagnosed with APL between 1992 and 2000 were identified. Data from 13 population based cancer registries that participate in the National Cancer Institute's SEER program were analyzed. The overall early death rate of all patients diagnosed with APL was 17.3% and 24% in patients aged > 55 years. The authors from the SEER study also concluded that this might be an underestimate due to the flaws in the SEER reporting system and the actual death rate may be higher than what the study showed. MD Anderson Cancer Center also reported their data from various time periods and compared them with SEER data.[3] From 2002-2008, the 5 year relative survival in APL patients was 64% which is contrary to the reported outcomes in clinical trials. This data also suggests that the lower survival observed in the general population may not be due to deaths in only smaller treatment centers but may also be a problem in larger metropolitan areas (Figure 1). Single institution experience from Stanford University showed an early death rate of 26%, suggesting that this problem might be pervasive even in larger treatment centers.[5]

Figure 1: Early Deaths in APL (Day 1 to 30)

Study	Total patients	Patients died	Mortality rate	Percentage of patients with hemorrhage in early death
**Brazil (2007)[4]**	134	43	32%	66%
**Swedish registry (2011)[1]**	105	30	29%	41%
**SEER data (2011)[2]**	1400	238	17% (24% in > 55yr)	Not discussed
**Stanford (2012)[5]**	70	19	26%	54%
**Ankara, Turkey, (2010)[8]**	49	20	40%	65%
**AIIMS, India (2011)[6]**	33	6	18.8%	Not discussed
**Germany > 60 years (2013)[9]**	91	24	26%	Not discussed
**Hiroshima, Japan > 65 years (2013)[7]**	32	7	21.3%	Not discussed
**GRU (our center) ASCO (2012)[10]**	19	7	37%	57%

Georgia Regents University Experience and Pilot Project:

At Georgia Regents University (GRU), Augusta, GA, from July 2005 to June 2009, 19 patients with APL were treated. Seven of these 19 (37%) patients died during induction due to the following reasons; however the 11 surviving patients are in molecular remission and are presumed cured with a median follow up of 2358 days.

1. Delay in diagnosis and transfer to a treating center
2. Bleeding from coagulopathy
3. Differentiation syndrome
4. Infections
5. Inconsistency in the treatment algorithm and physician inexperience.

After a thorough review, the investigators at GRU formulated a strategy to decrease early deaths and implemented it in 2010. This strategy aided physicians to rapidly diagnose the condition when suspected, and also follow a mechanism to rapidly transfer the patient from rural areas to GRU. A 1.5 page concise treatment algorithm to simplify the treatment process was developed and implemented at GRU. The algorithm was made available to physicians at outlying

smaller treatment centers as well. In addition, investigators at GRU communicated with treating physicians in the community and offered guidance. We treated or co-managed 143 patients thus far using this model, with 10 deaths (6.9%) Furthermore, after implementing the algorithm, data showed an encouraging decrease in induction mortality from an estimated 37% (N=19) to 6.9% (N=143) (Figure 2). Interestingly 40% of patients were treated in experienced leukemia centers and 60% were treated in outlying centers. Patients who survive the first 30 days have an excellent quality of life with a low chance of relapse and treatment related complications.

Figure 2: Survival Pre and Post Algorithm

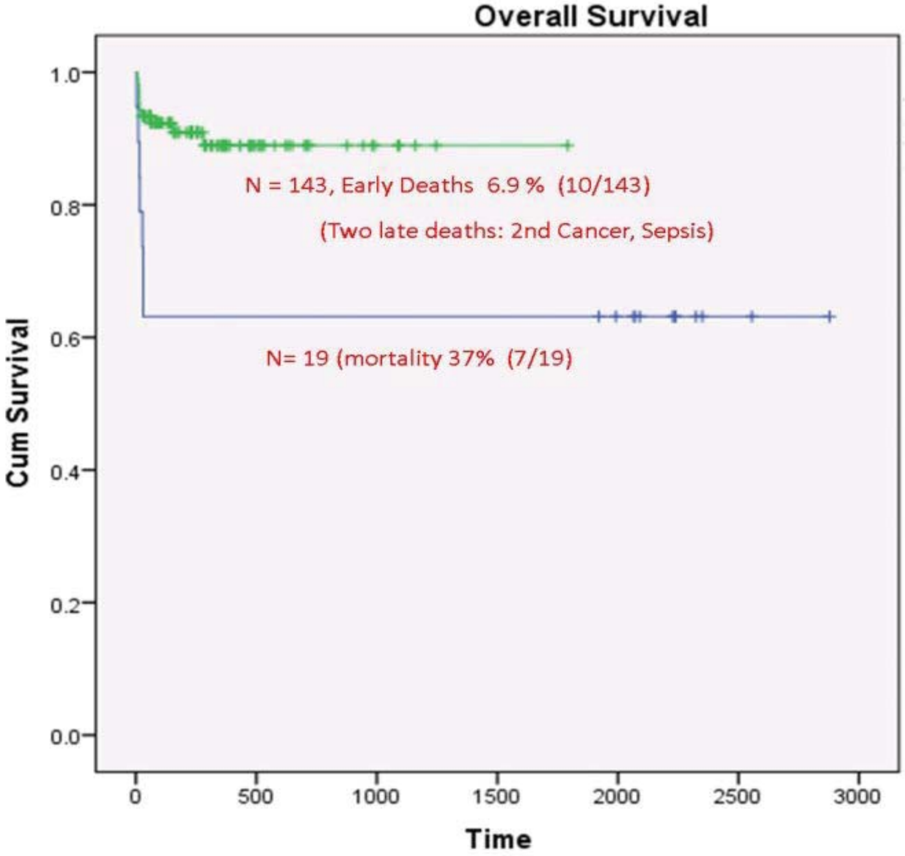

Ongoing Study:

Funding from the Leukemia & Lymphoma Society (LLS) supports the ongoing study efforts to follow the strategy developed at GRU as a model to implement it in the states of Georgia and South Carolina to cover a catchment population of 15 million; the goal is to reduce the induction mortality from an estimated 30% to under 15%.

1.2 Significance of the Study

As discussed earlier, the early mortality in APL continues to be high. The first large scale intervention to decrease early deaths in APL was undertaken by Rego et.al in Brazil, Mexico and Uruguay. In a report published in 2007, it was noted that in Brazil the mortality rate during induction in a cohort of 134 patients was 32%.[4] The cumulative mortality during induction and consolidation was

44.7%. The overall survival in this group was 52% which is significantly lower than what is expected in developed countries (62 to 86%). It was obvious that decreasing induction mortality would make the most impact in improving overall survival in this patient population. Analysis of the causes of early death showed that delayed diagnosis, hemorrhage, differentiation syndrome and infection were major causes of death. This led to the development and implementation of a strategy that addressed all the above mentioned causes of early mortality. This effort was supported by the International Members Committee of the American Society of Hematology (ASH) in 2005. A simple and uniform protocol was developed and launched in Brazil, Mexico, Chile and Uruguay. A standard program for rapid diagnosis, early initiation of treatment, aggressive management of coagulopathy, expedited transfer to a larger center, management of differentiation syndrome and effective treatment of infection was implemented. From 2006 to 2010, 183 patients were enrolled; 122 in Brazil, 30 in Mexico, 23 in Chile and 8 in Uruguay. The cases were individually discussed by a core group of experts with the treating hematologists in outlying hospitals via Internet. One third of the patients were high risk and 52% of the patients were intermediate risk. The induction mortality was 15% a decrease from 32% prior to the implementation of this study. The major cause of early deaths was still hemorrhage (48% of deaths). In addition, the overall survival (OS) improved from 52% to 80% at 2 year follow-up.(11)

APL is an uncommon disease; however excellent treatment modalities are available with cure rates of 90%. Current trials that are changing the treatment schedules, adding new drugs, or withholding maintenance might only have a minimal impact on survival in the general population. The intervention that will increase population wide survival is decreasing early deaths. Treating oncologists, who are not routinely involved in the care of acute leukemia patients in smaller centers as well as in larger treatment centers, may not be familiar with the treatment or expected complications. Addressing these issues by educating these physicians and co-managing patients will help improve survival in a condition that has a very high chance of cure.

Patients that were managed using the simple algorithm (Appendix I) demonstrated remarkable improvement in the overall survival. More importantly, a network was established with other leukemia treatment centers in the catchment area and helped local oncologists manage patients and decreased the mortality to 6.9% in 143 patients managed thus far. By replicating these results across a much wider population under the ECOG-ACRIN umbrella with additional sites, it is expected that adherence to the treatment guidelines and consultation with APL experts will reduce mortality rates even more than what previous trials have shown.

Seven of the ten deaths in our experience was in elderly population and although this is significantly better than published literature that suggests non clinical trial deaths to be as high as 50-60% in the same age groups, it shows that patients above the age of 60 years need a different approach. In our approach which is similar to the published literature from Chinese experience, using lower doses or single agent ATRA might lead to better outcomes even in this high risk population.

If the study is positive, efforts will be made to implement the concept nationally and manage APL patients with use of the streamlined guidelines and expert

support. A complement of experts is required to be made available permanently and the model should be followed for this project to be sustainable.

1.3 Study Overview

This is a multi-center study with 7 lead investigators accruing at 6 lead sites: Medical College of Georgia at Augusta University, Augusta, GA; Memorial Sloan Kettering Cancer Center, New York, NY; Mayo Clinic, Rochester, MN; Northwestern University, Chicago, IL; University of Pennsylvania, Philadelphia, PA and Mayo Clinic, Jacksonville, FL. The study will be restricted to the above

Rev. Add2 mentioned **SIX NCTN SITES.** All six sites will be referred to as lead sites**. Other Academic sites will not participate in this trial.**

Physicians treating patients at NCORP community centers will call one of the lead investigators as early as possible but no later than 3 days after APL directed treatment is started. The algorithm will be made available and the contacted lead investigator will provide guidance during induction and follow up treatment. The providing physicians will be encouraged to call as and when there are problems.

1.3.1 Treatment guidelines:

The treatment guidelines were developed after extensive review of the literature on standard treatment of APL. Physicians at the NCORP community centers will be provided this algorithm. The study does not exclude patients by age or comorbidities and complete adherence might not be feasible in certain clinical circumstances. This study is directed at co-managing patients by discussions between APL experts and treating physicians to improve the care.

1.3.2 Feasibility

The incidence of APL in the US is approximately 1,500 per year. Our goal is to target an accrual of 200 patients over a 4 year period. As this will be a data collection study, we expect to enroll these patients in this time frame. We do not plan to change the treatment from present standard of care and therefore will not include any new interventions. As described earlier, decreasing early mortality in this curable malignancy will have the biggest impact in improving outcomes. APL is a rare disease and physicians in some centers may not see enough APL patients to be proficient enough. Transferring these patients to larger academic centers is not always feasible for multiple reasons. As an increasing number of community hospitals are treating patients with acute leukemia, it is important to improve the survival in inexperienced centers as well. This would also make it easier for the patient and family and help them remain closer to home. From our experience in rural Georgia and South Carolina, most leukemia treatment centers have adequate supportive care infrastructure such as pharmacy, blood bank, pathology and sub-specialty services. We believe that expert guidance will ensure better adherence to the guidelines and earlier recognition of impending complications and thereby improve survival.

2. Objectives

2.1 Primary Objective

2.1.1 To evaluate if the proposed patient care strategy, that includes use of simplified Guidelines along with APL expert support, decreases the one-month induction mortality rate from 30% to under 15%.

2.2 Secondary Objectives

2.2.1 To assess the overall survival 1 year after accrual is completed.

2.2.2 To assess incidence and severity of differentiation syndrome

2.2.3 To correlate outcomes with time to initiation of ATRA from diagnosis or suspicion of diagnosis.

2.2.4 To evaluate outcomes in academic and community centers separately.

2.2.5 To evaluate factors associated with outcome.

3. Selection of Patients

3.1 Eligibility Criteria

_____ 3.1.1 All patients ≥ 18 years of age who are **confirmed** to have a diagnosis of APL, which is defined as:

_____ 3.1.1.1 Positive t(15:17) by FISH, OR

_____ 3.1.1.2 Positive t(15:17) by conventional karyotype, OR

_____ 3.1.1.3 Positive PML/RAR alpha by PCR

_____ 3.1.2 Patients must accept treatment and supportive care guidelines.

_____ 3.1.3 Referrals must be made as early as possible by treating physician (provider) but no later than 3 calendar days after ATRA or APL directed therapy is initiated. Consent can be obtained up until day 7 after initiating APL Directed Therapy.

Rev. Add2

_____ 3.1.4 Co-management can be started as soon as referral is made including weekends. The Physician at the NCORP community facility should make every effort to call the APL expert at the first suspicion of APL.

4. Registration Procedures

Rev. Add2

CTEP Registration Procedures

Food and Drug Administration (FDA) regulations and National Cancer Institute (NCI) policy require all individuals contributing to NCI-sponsored trials to register and to renew their registration annually. To register, all individuals must obtain a Cancer Therapy Evaluation Program (CTEP) Identity and Access Management (IAM) account (https://ctepcore.nci.nih.gov/iam). In addition, persons with a registration type of Investigator (IVR), Non-Physician Investigator (NPIVR), or Associate Plus (AP) (i.e., clinical site staff requiring write access to OPEN, RAVE, or TRIAD or acting as a primary site contact) must complete their annual registration using CTEP's web-based Registration and Credential Repository (RCR) (https://ctepcore.nci.nih.gov/rcr). Documentation requirements per registration type are outlined in the table below.

Documentation Required	IVR	NPIVR	AP	A
FDA Form 1572	✔	✔		
Financial Disclosure Form	✔	✔	✔	
NCI Biosketch (education, training, employment, license, and certification)	✔	✔	✔	
HSP/GCP training	✔	✔	✔	
Agent Shipment Form (if applicable)	✔			
CV (optional)	✔	✔	✔	

An active CTEP-IAM user account and appropriate RCR registration is required to access all CTEP and CTSU (Cancer Trials Support Unit) websites and applications. In addition, IVRs and NPIVRs must list all clinical practice sites and IRBs covering their practice sites on the FDA Form 1572 in RCR to allow the following:

- Added to a site roster
- Assigned the treating, credit, consenting, or drug shipment (IVR only) tasks in OPEN
- Act as the site-protocol PI on the IRB approval

Additional information can be found on the CTEP website at https://ctep.cancer.gov/investigatorresources/default.htm. For questions, please contact the RCR *Help Desk* by email at RCRHelpDesk@nih.gov.

CTEP Associate Registration Procedures / CTEP-IAM Account

The Cancer Therapy Evaluation Program (CTEP) Identity and Access Management (IAM) application is a web-based application intended for use by both Investigators (i.e., all physicians involved in the conduct of NCI-sponsored clinical trials) and Associates (i.e., all staff involved in the conduct of NCI-sponsored clinical trials).

Associates will use the CTEP-IAM application to register (both initial registration and annual re-registration) with CTEP and to obtain a user account.

Investigators will use the CTEP-IAM application to obtain a user account only. (See CTEP Investigator Registration Procedures above for information on registering with CTEP as an Investigator, which must be completed before a CTEP-IAM account can be requested.)

An active CTEP-IAM user account will be needed to access all CTEP and CTSU (Cancer Trials Support Unit) websites and applications, including the CTSU members' website.

Additional information can be found on the CTEP website at <http://ctep.cancer.gov/branches/pmb/associate_registration.htm>. For questions, please contact the ***CTEP Associate Registration Help Desk*** by email at <ctepreghelp@ctep.nci.nih.gov>.

CTSU Registration Procedures

This study is supported by the NCI Cancer Trials Support Unit (CTSU).

Rev. Add2

IRB Approval:

Each investigator or group of investigators at a clinical site must obtain IRB approval for this protocol and submit IRB approval and supporting documentation to the CTSU Regulatory Office before they can be approved to enroll patients. Assignment of site registration status in the CTSU Regulatory Support System (RSS) uses extensive data to make a determination of whether a site has fulfilled all regulatory criteria including but not limited to the following:

- An active Federal Wide Assurance (FWA) number
- An active roster affiliation with the Lead Network or a participating organization
- A valid IRB approval
- Compliance with all protocol specific requirements.

In addition, the site-protocol Principal Investigator (PI) must meet the following criteria:

- Active registration status
- The IRB number of the site IRB of record listed on their Form FDA 1572
- An active status on a participating roster at the registering site.

Sites participating on the NCI CIRB initiative that are approved by the CIRB for this study are not required to submit IRB approval documentation to the CTSU Regulatory Office. For sites using the CIRB, IRB approval information is received from the CIRB and applied to the RSS in an automated process. Signatory Institutions must submit a Study Specific Worksheet for Local Context (SSW) to the CIRB via IRB Manager to indicate their intent to open the study locally. The CIRB's approval of the SSW is then communicated to the CTSU Regulatory Office. In order for the SSW approval to be processed, the Signatory Institution must inform the CTSU which CIRB-approved institutions aligned with the Signatory Institution are participating in the study.

Rev. Add2

Downloading Site Registration Documents:

Site registration forms may be downloaded from the **EA9131**protocol page located on the CTSU members' website.

- Go to https://www.ctsu.org and log in to the members' area using your CTEP-IAM username and password
- Click on the Protocols tab in the upper left of your screen
- Either enter the protocol # in the search field at the top of the protocol tree, or
- Click on the By Lead Organization folder to expand
- Click on the **ECOG-ACRIN** link to expand, then select trial protocol **EA9131**

- Click on LPO Documents, select the Site Registration documents link, and download and complete the forms provided.

Rev. Add2 **Requirements for EA9131 Site Registration:**

- IRB approval (For sites not participating via the NCI CIRB; local IRB documentation, an IRB-signed CTSU IRB Certification Form, Protocol of Human Subjects Assurance Identification/IRB Certification/Declaration of Exemption Form, or combination is accepted)

Rev. Add2 **Submitting Regulatory Documents**

Submit required forms and documents to the CTSU Regulatory Office via the Regulatory Submission Portal, where they will be entered and tracked in the CTSU RSS.

Regulatory Submission Portal: www.ctsu.org (members' area) → Regulatory Tab → Regulatory Submission

When applicable, original documents should be mailed to:

CTSU Regulatory Office
1818 Market Street, Suite 1100
Philadelphia, PA 19103

Institutions with patients waiting that are unable to use the Portal should alert the CTSU Regulatory Office immediately at 1-866-651-2878 in order to receive further instruction and support.

Rev. Add2 **Required Protocol Specific Regulatory Documents**

1. Copy of IRB Informed Consent Document.

   NOTE: At the main sites, the PI and other designated investigators will obtain the consent.

   If a patient is being treated at an NCORP community site, the local PI or Co/Sub-PI will consent the patient. The treating physician at the NCORP community site will contact an APL expert as soon as the diagnosis is suspected but no later than 3 days after ATRA has been initiated.

   NOTE: Any deletion or substantive modification of information concerning risks or alternative procedures contained in the sample informed consent document must be justified in writing by the investigator and approved by the IRB.

2. A. CTSU IRB Certification Form.
   Or
   B. Signed HHS OMB No. 0990-0263 (replaces Form 310).
   Or
   C. IRB Approval Letter

   NOTE: The above submissions must include the following details:

   - Indicate all sites approved for the protocol under an assurance number.
   - OHRP assurance number of reviewing IRB
   - Full protocol title and number
   - Version Date
   - Type of review (full board vs. expedited)

- Date of review.
- Signature of IRB official

Rev. Add2 **Checking Your Site's Registration Status:**

You can verify your site registration status on the members' section of the CTSU website.

- Go to https://www.ctsu.org and log in to the members' area using your CTEP-IAM username and password
- Click on the Regulatory tab
- Click on the Site Registration tab
- Enter your 5-character CTEP Institution Code and click on Go

NOTE: The status given only reflects compliance with IRB documentation and institutional compliance with protocol-specific requirements outlined by the Lead Network. It does not reflect compliance with protocol requirements for individuals participating on the protocol or the enrolling investigator's status with the NCI or their affiliated networks.

Patient enrollment will be facilitated using the Oncology Patient Enrollment Network (OPEN). OPEN is a web-based registration system available on a 24/7 basis. To access OPEN, the site user must have an active CTEP-IAM account (check at <https://ctepcore.nci.nih.gov/iam>) and a 'Registrar' role on either the LPO or participating organization roster. Registrars must hold a minimum of an AP registration type.

The Study Chair Liaison will use OPEN to enroll patients to this study. It is integrated with the CTSU Enterprise System for regulatory and roster data, and upon enrollment, initializes the patient in the Rave database. OPEN can be accessed at https://open.ctsu.org or from the OPEN tab on the CTSU members' side of the website at https://www.ctsu.org. To assign an IVR or NPIVR as the treating, crediting, consenting, drug shipment (IVR only), or investigator receiving a transfer in OPEN, the IVR or NPIVR must list on their Form FDA 1572 in RCR the IRB number used on the site's IRB approval.

Prior to accessing OPEN, site staff should verify the following:

- All eligibility criteria have been met within the protocol stated timeframes.
- All patients have signed an appropriate consent form and HIPAA authorization form (if applicable).

NOTE: The OPEN system will provide the site with a printable confirmation of registration and treatment information. The Study Chair Liaison will print this confirmation and provide it to the site for records.

Further instructional information is provided on the OPEN tab of the CTSU members' side of the CTSU website at https://www.ctsu.org or at https://open.ctsu.org. For any additional questions contact the CTSU Help Desk at 1-888-823-5923 or ctsucontact@westat.com.

4.1 Protocol Number

4.2 Investigator Identification

- Institution and affiliate name
- Investigator's name

4.3 Patient Identification

- Patient's initials (first and last)
- Patient's Hospital ID and/or Social Security number
- Patient demographics
    - Gender
    - Birth date (mm/yyyy)
    - Race
    - Ethnicity
    - Nine-digit ZIP code
    - Method of payment
    - Country of residence

4.4 Eligibility Verification

Patients must meet all of the eligibility requirements listed in Section 3.

4.5 Additional Requirements

Rev. Add2 4.5.1 Patients must provide a signed and dated, written informed consent form. This document may be signed patient's next of kin or person with power of attorney, if necessary.

NOTE: Copies of the consent are not collected by the ECOG-ACRIN Operations Office

Rev. Add2 **NOTE:** Patients should be consented only after APL diagnosis is confirmed.

Rev. Add2 4.5.1.1 Data collection for this study will be done exclusively through the Medidata Rave clinical data management system. Access to the trial in Rave is granted through the iMedidata application to all persons with the appropriate roles assigned in Regulatory Support System (RSS). To access Rave via iMedidata, the site user must have an active CTEP-IAM account (check at https://ctepcore.nci.nih.gov/iam) and the appropriate Rave role (Rave CRA, Read-Only, CRA, Lab Admin, SLA, or Site Investigator) on either the LPO or participating organization roster at the enrolling site. To the hold Rave CRA role or CRA Lab Admin role, the user must hold a minimum of an AP registration type. To hold the Rave Site Investigator role, the individual must be registered as an NPIVR or IVR. Associates can hold read-only roles in Rave.

Upon initial site registration approval for the study in RSS, all persons with Rave roles assigned on the appropriate roster will be sent a study invitation e-mail from iMedidata. To accept the invitation, site users must log into the Select Login (https://login.imedidata.com/selectlogin) using their CTEP-IAM user name and password, and click on the "accept" link in the upper right-corner of the iMedidata page. Please note, site users will not be able to access the

study in Rave until all required Medidata and study specific trainings are completed. Trainings will be in the form of electronic learnings (eLearnings), and can be accessed by clicking on the link in the upper right pane of the iMedidata screen.

Users that have not previously activated their iMedidata/Rave account at the time of initial site registration approval for the study in RSS will also receive a separate invitation from iMedidata to activate their account. Account activation instructions are located on the CTSU website, Rave tab under the Rave resource materials (Medidata Account Activation and Study Invitation Acceptance). Additional information on iMedidata/Rave is available on the CTSU members' website under the Rave tab at www.ctsu.org/RAVE/ or by contacting the CTSU Help Desk at 1-888-823-5923 or by e-mail at ctsucontact@westat.com.

5. Methodology Plan

There will be 7 investigators accruing at 6 different centers (LEAD SITES) who will serve as the APL experts for this proposal. The patients treated at these 6 centers will be enrolled at their particular institution. **The study will not be open at other academic centers**. The APL expert at each site will either manage the patient or provide consultative oversight to the treating team. The sub-investigators at lead sites can consent patients but it is expected that the local PI will provide oversight. We expect to enroll a maximum of 30% of the patients at these lead sites. Induction mortality is a problem in larger centers as well from extensive data that has been published. We postulate that using the guidelines along with expert input will improve outcome in lead sites. This will give us the opportunity to assess the problem as well as outcome in academic centers and compare the results to community centers. Since the accrual goal is 200 patients
Rev. Add2 over a 4 year period, the accrual at lead centers will be limited to 60 patients over a 4-year period (30%). The Study Chair and ECOG-ACRIN office will monitor this closely and once the target is achieved, enrollment at lead sites will be put on hold. Investigators from the LEAD sites will meet or have a conference call twice a year to discuss the progress and results.

Prior to enrolling patients, while the study is being reviewed by the IRBs at the respective sites, the study investigators will contact NCORP community centers that are likely to treat APL in their catchment area. These visits will also be a part of publicizing the trial to ensure other treating physicians are aware of the study and also the risk of early deaths in this disease. In addition to physician visits, flyers and regular emails will also be sent out to ensure that there are frequent reminders about the study. At sites that open the trial there will also be a webinar to raise awareness for the trial and provide training on the treatment guidelines prior to study activation.

The trial will be conducted at NCORP community sites in addition to the Lead Sites. We conducted a survey of NCORP sites to assess their interest in participating in the study. The survey had 3 questions. a. Does your center treat patients with APL? b. Are you aware the induction mortality in APL is approximately 30%? c. Will your Center participate in a trial focused on decreasing induction mortality? Eleven sites responded and expressed interest. The PI and Co-PI will make personal contact with the other NCORP sites and present the goals of the trial and get other sites to open. We have also asked the NCORP leadership to give us an opportunity to participate in their annual meeting and conference calls and publicize the study to get more sites to participate.

5.1 Lead Sites

5.1.1 Lead Sites

- Medical College of Georgia at Augusta University.
- Memorial Sloan-Kettering Cancer Center, New York, NY
- The Mayo Clinic, Rochester, MN
- Northwestern University Feinberg School of Medicine, Chicago, IL
- Abramson Cancer Center of the University of Pennsylvania, Philadelphia, PA
- The Mayo Clinic, Jacksonville, FL

5.1.2 Patients treated at Lead Sites will be treated locally at the Lead Site using treatment guidelines. The local PI will provide expert support to

the treating team. The presentation of APL can be highly variable; hence adherence to the guidelines is not always feasible. Deviations that are made from the guidelines based on particular clinical situations will be documented in the medical record.

5.2 Outlying NCORP Community Facility

5.2.1 The study will be opened only at the NCORP community sites. **It will not be open at academic NCORP centers.** The treating physician from the NCORP community Facility should call the APL Expert in their catchment area for treatment guidance. Seeking advice from an APL expert is a key component of our model. The call should be made as soon as APL is suspected but no later than 3 days after starting ATRA and communication should be ongoing based on the clinical developments until a hematologic remission is achieved. The presentation in APL can be highly variable; hence complete adherence to the guidelines is not always feasible. Deviations that are made from the guidelines based on particular clinical situations will be documented in the medical record.

5.2.1.1 The provider at NCORP community centers will be the Hematologist/Oncologist attending caring for the patient. Initial contact has to be made by a Hematologist/Oncologist.

5.2.1.2 It is expected that the initial contacting Hematologist/Oncologist will continue the communication; alternatively, other members of the practice can communicate with the APL expert on an ongoing basis.

5.2.1.3 Trainees or advanced level practice providers (physician assistants or nurse practitioners) and trainees can be in communication subsequently with APL experts to continue the care.

5.2.2 A log will be maintained by the APL expert (Please see Appendix II). This will include information on Date admitted, date APL suspected, date APL confirmed, date APL therapy started and date APL expert was contacted. Subsequent calls and decisions made will be documented on the log sheet.

5.2.3 It will be suggested that the treating physician will also document the communication on an ongoing basis.

5.3 Study Accrual and Timeline

The study will accrue from years one to 4. Follow up will be for one year after last patient is accrued and accrual is completed. The total study duration is 5 years.

5.4 Adverse Event Reporting Requirements

The adverse events to treatment will be collected from the hospital records obtained from the patients induction period and also follow up at the treating physician’s office. An AE log need not be maintained since this study will not include any new drugs. The complications from treatment will be noted in the database and will be used for analysis of outcomes. The only SAE that has to be reported is death.

5.4.1 Reporting Procedure

This study requires that expedited serious adverse event reporting use CTEP's Adverse Event Reporting System (CTEP-AERS). The CTEP's guidelines for CTEP-AERS can be found at http://ctep.cancer.gov. A CTEP-AERS report must be submitted electronically to ECOG-ACRIN and the appropriate regulatory agencies via the CTEP-AERS Web-based application located at http://ctep.cancer.gov.

In the rare event when Internet connectivity is disrupted a 24-hour notification is to be made by telephone to

Rev. Add2

- the SAE Team at ECOG-ACRIN (857-504-2900)
- the NCI (301-897-7497)

An electronic report MUST be submitted immediately upon re-establishment of internet connection.

Supporting and follow up data: Any supporting or follow up documentation must be uploaded to the Supplemental Data Folder in Medidata Rave within 48-72 hours. In addition, supporting or follow up documentation must be faxed to the NCI (301- 230-0159) in the same timeframe.

CTEP Technical Help Desk: For any technical questions or system problems regarding the use of the CTEP-AERS application, please contact the NCI Technical Help Desk at ncictephelp@ctep.nci.nih.gov or by phone at 1-888-283-7457.

Rev. Add2

5.4.2 Expedited Reporting Requirements for EA9131

**Grade 5[a]**	
Unexpected	Expected
24-Hour 5 Calendar Days	24-Hour 5 Calendar Days
**24-Hour; 5 Calendar Days** – The AE must initially be reported via CTEP-AERS within 24 hours of learning of the AE, followed by a complete expedited report within 5 calendar days of the initial 24-hour report.	
[a] This includes all deaths within one year of registration, regardless of cause. Thereafter, deaths must only be reported on the protocol specific case report forms.<br>**NOTE:** A death due to progressive disease should be reported as a Grade 5 "Disease progression" under the System Organ Class (SOC) "General disorder and administration site conditions". Evidence that the death was a manifestation of underlying disease (e.g. radiological changes suggesting tumor growth or progression: clinical deterioration associated with a disease process) should be submitted.	

5.4.3 Other recipients of adverse event reports and supplemental data

ECOG-ACRIN will forward CTEP-AERS reports to the appropriate regulatory agencies and pharmaceutical company, if applicable.

Serious Adverse events determined to be reportable via CTEP-AERS must also be reported by the institution, according to the local policy

and procedures, to the Institutional Review Board responsible for oversight of the patient.

5.5 Completion of Induction

Induction will be continued until the patient achieves hematologic remission (as per guidelines).

5.6 Consolidation

Consolidation will be at the treating physician's discretion.

5.7 Maintenance

Maintenance will be per treating physician's discretion.

5.8 Duration of Follow-up

For this protocol, all patients, including those who discontinue treatment early, will be followed for survival for 1 year after the accrual is completed.

6. Study Parameters

NOTE: Please refer to Appendix I for all recommended tests and procedures

	**Baseline**
Confirmed APL Dianosis[1]	X
Referral to APL Expert[2]	X
Informed Consent[3]	X
Subject Registration[4]	X

Rev. Add2

1. Positive t(15:17) by FISH OR conventional karyotype OR PML/RARα by PCR, Rare variants can also be included if confirmed.
2. It is preferred that the APL expert for the catchment area is contacted, however, any APL Expert at any Lead Site may be contacted. Referrals must be made within 3 calendar days after ATRA or APL directed therapy is initiated.

Rev. Add2

3. Written informed consent must be obtained within 7 days of initiating APL directed therapy.
4. Registration can be done after initiating treatment.

7. Statistical Considerations

7.1 Study Design and Objectives

This study will accrue 200 APL patients over a 4-year period. The primary hypothesis is that the proposed process will lower the one-month mortality rate from 30% to < 15%. The primary endpoint is the one-month mortality rate Secondary endpoints include: i) overall survival, one-year survival rate, ii) safety, iii) initiation of treatment from diagnosis, iv) survival in academic and community centers v) compliance with treatment guidelines and vi) evaluation of factors associated with outcome.

7.2 Sample Size Considerations and Monitoring Plan

Of the 200 patients accrued, it is expected to have 180 eligible patients after adjusting for ineligible and/or lost-to-follow up cases. We allowed 10% for ineligible and/or lost-to-follow-up cases. We often allow additional cases (up to 10%) to account for ineligible cases in ECOG studies. We do not expect any lost-to-fu cases at 1 month. However, we used 10% ineligibility and/or lost-to-follow-up to be conservative and general in this study.

After 100 patients (for 90 eligible) are enrolled and have followed for one-month mortality endpoint, one interim analysis will be conducted. Using the critical value corresponding to the O'Brien and Fleming boundary at 50% information time, 95% Repeated Confidence Interval (RCI) for the one-month mortality rate will be constructed. The result will be discussed with the ECOG-ACRIN DSMC members. If deemed efficacious, accrual will continue to 200 (for 180 eligible) patients. This design will have at least 90% power with a two-sided type I error rate of < 0.05. Another rationale for the sample size of 200 is that we would like to build a 95% RCI with a tight interval. For the expected one-month mortality rate, if the observed rate is 10% (18/180) the 95% RCI will be 6-15.4%.If the observed one-month mortality rate is 15%, the 95% RCI will be 10.1-21.1%.

We plan to limit the accrual from the large academic centers to a maximum of 30% of the total patients. Thus 60 patients (54 eligible) from major academic centers and 140 (126 eligible) from NCORP community sites are anticipated. The primary analysis and monitoring will be based on all patients regardless of enrollment site. After the study is completed, one-month mortality rate will be estimated for patients enrolled from NCORP and academic sites separately as a secondary analysis. With 126 eligible patients from NCORP sites, there will be at least 90% power for the hypothesis testing of reducing mortality rate from 30% to 15%, with a two-sided error rate of 0.05. There is sufficient power to test if one-month mortality rate for NCORP sites can be reduced from 30% to < 15%

For NCORP sites, 95% CI is 5.6-17% for the observed one-month mortality rate of 10.3% (13/126) and 95% CI is 9.3-22.5% for the observed one-month mortality rate of 15.1%. (19/126). With 54 eligible patients from academic sites, there will be less than 80% power for the hypothesis testing of reducing mortality rate from 30% to 15%, with a two-sided error rate of 0.05. For academic sites, 95% CI is 3-20.3%% for the observed one-month mortality rate of 9.3% (5/54) and 95% CI is 6.6-27.1% for the observed one-month mortality rate of 14.8%. (8/54). Statistical analysis will be mostly descriptive for the secondary endpoints.

At 50% information time (i.e. 90 eligible patients have one-month mortality assessed), 95% Repeated Confidence Interval will be built for the one-month mortality estimate using the O'Brien-Fleming boundary for 50% information time. This analysis will be presented to the ECOG-ACRIN DSMC and discussed further.

We do not expect major safety issues in this study. However adverse events data will be monitored in ongoing basis and will be brought to ECOG-ACRIN DSMC attention.

7.3 Statistical Analysis Plan

7.3.1 Primary Objective

One-month mortality rate will be estimated by dividing total number of deaths within one month of study entry by total number of cases with at least one-month follow up data. 95% RCI will be provided for this endpoint. We will also evaluate the OS data using the Kaplan-Meier plot.

7.3.2 Secondary Objectives

Using the method of Kaplan-Meier, overall survival (OS) data will be described. The median OS and one-year OS rate will be estimated and 95% CI will be provided.

We confirm that the primary objective is to assess one-month mortality rate among all cases. Secondary analysis will involve evaluating outcome data in NCORP and academic sites separately. We clarified this further in Section 7.2. Overall the expected one-month mortality rate is in the range of 10-15% across all patients as well as in two subgroups of NCORP and academic sites.

With 200 (180 eligible) patients, we expect to build a confidence interval not wider than 11% for the expected one-month mortality rate of 10-15%. With 140 (126 eligible) cases from NCORP sites, the confidence interval for the one-month mortality rate will be not be wider than 13%.

For the safety endpoints, the severity and duration of coagulopathy, bleeding, infections and differentiation syndrome will be measured based on the CTC version 4.0. Analysis of the safety data will be mostly descriptive. The proportion of patients with the worst degree toxicity of grade 3 or higher will be assessed and presented with 95% CI. The length of hospital stay is defined as a period between the discharge and admission dates for the first hospitalization. For patients with no hospitalization, this will be defined as 0. To assess the associations between the length of hospital stay and toxicity measures (bleeding, infections, differentiation syndrome), each toxicity measures will be dichotomized as "mild" vs. "severe" and the length of hospital stay will be compared using the two-sample t-test.

The time from diagnosis and initiation of treatment will be assessed and dichotomized as "short' vs. "long" using the median value. One-month mortality rate will be compared in these two groups (short vs. long) using the Fisher's exact test. Overall-survival will also be

compared in these two groups using the logrank test. In this analysis, the time of diagnosis will be determined by both: i) confirmed diagnosis date and ii) date of suspicious APL. The dichotomized value of long vs. short based on the both times will be evaluated. Similarly the one-month morality rate and overall survival between the lead institutions and NCORP community centers will be compared. For all comparisons, two-sided p-values will be reported.

Compliance with treatment guidelines will be assessed as binary outcome and the successful compliance rate will be described with 95% confidence interval. A regression model will be developed to evaluate the factors associated with outcome. Data items such as age, risk status, comorbidity will be collected and evaluated.

Rev. Add2

7.4 Race, Gender, and Ethnicity Accrual Estimates

Racial Categories	Ethnic Categories				Total
	Hispanic or Latino		Not Hispanic or Latino		
	Females	Males	Females	Males	
American Indian or Alaskan Native	0	0	0	0	0
Asian	0	0	0	2	2
Native Hawaiian or other Pacific Islander	0	0	0	0	0
Black or African American	0	0	12	4	16
White	0	0	84	98	182
Total	0	0	96	104	200

8. Electronic Data Capture

Please refer to the **EA9131** Forms Completion Guidelines for the forms submission schedule. Data collection will be performed exclusively in Medidata Rave. In case of withdrawal from the study, data will be collected to the point of withdrawal.

9. Patient Consent and Peer Judgment

Current FDA, NCI, state, federal and institutional regulations concerning informed consent will be followed.

10. References

1. Lehmann S, Ravn A, Carlsson L, Antunovic P, Deneberg S, Mollgard L, et al. Continuing high early death rate in acute promyelocytic leukemia: a population-based report from the Swedish Adult Acute Leukemia Registry. Leukemia. 2011;25(7):1128-34.
2. Park JH, Qiao B, Panageas KS, Schymura MJ, Jurcic JG, Rosenblat TL, et al. Early death rate in acute promyelocytic leukemia remains high despite all-trans retinoic acid. Blood. 2011;118(5):1248-54.
3. Chen Y, Kantarjian H, Wang H, Cortes J, Ravandi F. Acute promyelocytic leukemia: a population-based study on incidence and survival in the United States, 1975-2008. Cancer. 2012;118(23):5811-8.
4. Jacomo RH, Melo RA, Souto FR, de Mattos ER, de Oliveira CT, Fagundes EM, et al. Clinical features and outcomes of 134 Brazilians with acute promyelocytic leukemia who received ATRA and anthracyclines. Haematologica. 2007;92(10):1431-2.
5. McClellan JS, Kohrt HE, Coutre S, Gotlib JR, Majeti R, , et al.Treatment advances have not improved the early death rate in acute promyelocytic leukemia. Haematologica.2012;97(1):133-6.
6. Bajpai J1, Sharma A, Kumar L, Dabkara D, Raina V, Kochupillai V, Kumar R. Acute promyelocytic leukemia: an experience from a tertiary care centre in north India. Indian J Cancer. 2011 Jul-Sep;48(3):316-22.
7. Imagawa J, Harada Y, Shimomura T, Tanaka H, Okikawa Y, Harada H. High early death rate in elderly patients with acute promyelocyticleukemia treated with all-trans retinoic acid combined chemotherapy. Int J Hematol. 2013 Aug;98(2):264-6.
8. Serefhanoglu, S., et al. (2010). "Clinical features and outcomes of 49 Turkish patients with acute promyelocytic leukemia who received ATRA and anthracyclines (PETHEMA protocol) therapy." Leuk Res 34(12): e317-319
9. Lengfelder, E., et al. (2013). "Outcome of elderly patients with acute promyelocytic leukemia: results of the German Acute Myeloid Leukemia Cooperative Group." Ann Hematol 92(1): 41-52
10. Anand P. Jillella, Ibrahim Sadek, Devi Morrison, Kavita Natrajan, Farrukh Tauseef Awan, Ravindra Kolhe, Vamsi Kota. A simple but effective model to decrease early deaths in acute promyelocytic leukemia (APL). J Clin Oncol 30, 2012 (suppl; abstr 6573)
11. Rego EM, Kim HT, Ruiz-Arguelles GJ, Undurraga MS, Uriarte Mdel R, Jacomo RH, et al. Improving acute promyelocytic leukemia (APL) outcome in developing countries through networking, results of the International Consortium on APL. Blood. 2013;121(11):1935-43.

A Simplified Patient Care Strategy to Decrease Early Deaths in Acute Promyelocytic Leukemia (APL)

Appendix I

Acute Promyelocytic Leukemia Treatment Guidelines

Developed at Georgia Regents University, August 2013 Revised May 2017

AUTHORS: Dr. Anand P. Jillella Mobile: 706 -951- 5144 Office: 706-721-2505
Dr. Vamsi K. Kota Mobile: 706- 825- 4091 Office: 404-778-1900

WORK-UP

BASELINE

- Chest X-ray
- Echocardiogram and EKG
- PICC line placement – NO central lines in chest or neck or invasive procedures (bronchoscopy, spinal tap, endoscopy)
- Bone marrow examination (aspirate, biopsy, flow, cytogenetics, FISH for t15:17, PCR for PML-RARa). Day 14 marrow is **NOT** necessary.

DAILY

- CBC, CMP, PT, PTT, fibrinogen **TWICE DAILY** until lab & clinical coagulopathy is resolved. CMP daily and twice a day as dictated by clinical condition.
- D-dimer **ONCE DAILY** for **ENTIRE** hospitalization.

PREVENTION/TREATMENT OF COAGULOPATHY

- Intracranial, pulmonary and GI hemorrhages are frequent
- Risk of hemorrhage is ↑ with the presence of any of the following - active sites of bleeding, ↓ fibrinogen, ↑ D-dimers, ↑ PT & PTT, ↑ WBC, ↑peripheral blasts, renal failure and poor performance status;

Rev. Add2
- **Keep platelets above 50,000 (until resolution of DIC)**
- If there are any active sites of bleeding on presentation (e.g., needle sticks, bone marrow biopsy sites,), and prolonged PT/PTT), give 4 units of fresh frozen plasma (FFP) at the start of ATRA and chemotherapy. **CONTINUE** FFP support **TWICE A DAY** until clinical bleeding resolves.

Rev. Add2
- **Keep fibrinogen above 150**. Use 10 units of cryoprecipitate if < 150 (until DIC resolves).
- When clinical & lab coagulopathy is resolved, blood product support is per standard guidelines.

⟶ APL IS A MEDICAL EMERGENCY - START ATRA ASAP ⟵

PREVENTION/TREATMENT OF APL DIFFERENTIATION SYNDROME

Differentiation Syndrome-symptoms are – dyspnea, unexplained fever, ↑ weight, peripheral edema, unexplained hypotension, acute renal failure, CHF, pleuro-pericardial effusions, and interstitial pulmonary infiltrates.

- Daily weights - **BEDSIDE SCALES ONLY**
- Keep I/O matched – **METICULOUSLY**

Rev. Add2
- Diuretics if increase in fluid retention or weight.
- Prednisone 0.5 to 1 mg/Kg in all patients for 2 weeks as prophylaxis starting on day 1 followed by a taper.
- Increase Dexamethasone to 10 mg IV BID from the prophylactic dose above, if onset of DS symptoms.
- If WBC > 10,000, dexamethasone 10 mg IV bid could be started on day 1 before initiating ATRA.
- Temporary discontinuation of ATRA or Arsenic Trioxide (ATO) is indicated only in case of severe cases of DS.
- Continue dexamethasone until 3 days after all symptoms are resolved. ATRA and/or arsenic can be resumed.

SUPPORTIVE CARE

- Allopurinol 300 mg daily.
- Antibiotic prophylaxis - levofloxacin 500 mg po daily or similar antibiotic.
- Antifungal prophylaxis - posaconazole 200 mg po 3 x3x daily, voriconazole 200 mg po 2 x2x daily, Micafungin 50 mg daily or a similar drug as per institutional standard.
- Anti-viral prophylaxis - acyclovir 400 mg po 2 x2x daily or valacyclovir 1000 mg PO daily.
- RBC transfusion recommended when HGB ↓ 8.

INDUCTION OF LOW AND INTERMEDIATE RISK PATIENTS WBC < 10,000 and PLTS < 40,000 -

- ATRA 45mg/m2 in divided doses twice a day plus ATO 0.15 kg daily until complete hematologic remission (no anthracycline). Prednisone 0.5 mg/kg for 14 days followed by a gradual taper if no evidence of DS.

  OR
- ATRA on Day 1 at 45 mg/m2 DAILY in divided doses twice a day until complete hematologic remission.
- Idarubicin 12 mg/m2 on Days 2, 4, 6 and 8. Prednisone 0.5 mg/kg for 14 days followed by a gradual taper if no evidence of DS.

Rev. Add2 **INDUCTION OF HIGH RISK PATIENTS WBC > 10,000**

- ATRA to be started as soon as diagnosis is suspected at 45 mg/m2 in divided doses twice a day until complete hematologic remission.
- Idarubicin to be started on the SAME DAY OR AS SOON AS POSSIBLE WHEN THE DIAGNOSIS IS CONFIRMED at 12 mg/M2 on days 1, 3, 5 and 7.
- Dexamethasone 10 mg IV bid on days 1 to 14 followed by a taper.
- Arsenic at 0.15 mg/Kg can be started on day 10.
- Even if the genetic results are not available, it is reasonable to start Idarubicin if the diagnosis is otherwise strongly suggested.

Rev. Add2 **ARSENIC ADMINISTRATION**

- Prednisone 0.5 mg/Kg from days 1-14 days followed by a taper. If high risk, dexamethasone 10 mg IV bid on days 1 to 14 followed by a taper.
- Watch for differentiation syndrome.

- Watch for prolonged QTc interval.
- Keep Mg above 2.0 and K above 4.0.
- Follow LFTs for grade 2 - 4 liver dysfunction. If this occurs HOLD arsenic.

SUGGESTED MANAGEMENT OF ATRA/ARSENIC-INDUCED LEUKOCYTOSIS

- WBC 5 - 50 K- Hydroxyurea 500 mg qid
- WBC > 50K- Hydroxyurea 1000 mg qid
- If leukocytosis does not resolve one or two doses of Idarubicin or Cytarabine could be considered.
- **NO LEUKOPHERESIS.**

ELDERLY PATIENTS / ≥ 60 years AND PATIENTS WITH SEVERE COMORBID CONDITIONS

- May not be candidates for cytotoxic chemotherapy.
- Consider dose reduction of ATRA. Single agent and consider 25 mg/M2 for 14 days.
- Consider dose reduction of ATO and consider 0.075 mg/M2 and add on day 15.

A Simplified Patient Care Strategy to Decrease Early Deaths in Acute Promyelocytic Leukemia (APL)

Appendix II

APL Expert and NCORP Provider Communication

1. Date patient admitted -
2. Date APL suspected -
3. Date APL confirmed -
4. Date APL therapy started -
5. Date APL expert contacted -
6. Subsequent communications -

Date:

Date:

Date:

Date:

Date:

Date:

Date:

Date:

Date:

Date:

A Simplified Patient Care Strategy to Decrease Early Deaths in Acute Promyelocytic Leukemia (APL)

Appendix III

NCORP PROVIDER INFORMATION SHEET

This is a research study focused on decreasing induction mortality in Acute Promyelocytic Leukemia (APL). Historical data shows an induction mortality of 30% in the general population and the purpose of this study is to decrease it to < 15%. NCORP providers will be given this information sheet along with the treatment guidelines in Appendix I.

A. The study will consist of using the simplified APL treatment algorithm that is attached.

B. Equally important is for the provider at NCORP sites to call an APL expert listed in the protocol or the PI/Co-PI. Cell phone numbers of APL experts are in the Protocol.

C. The APL expert will function as a resource during induction to prevent and manage complications. It is suggested that the provider at NCORP sites call the APL expert as frequently as needed during the first 4 weeks. The experts are available 24/7.

D. NCORP providers will document discussion with the APL expert and clinical decisions in the Medical Record. APL experts will fill the Expert/NCORP Provider communication sheet.

E. There are no obvious discomforts to the patient since the patient will be treated on the accepted standard of care. Expert support may help in preventing and managing complications and improving outcome.

F We will retain all patient records and signed informed consent forms filed in a secure location. Database is maintained using web-based database system. Only research members are granted access to web-based database system with user ID and password.

G. Participation is voluntary and the patient may choose not to participate in the trial. In that case the patient will be treated per usual treatment guidelines. Participants may discontinue at any time without penalty or loss of benefits.

A Simplified Patient Care Strategy to Decrease Early Deaths in Acute Promyelocytic Leukemia (APL)

Appendix IV

Patient Thank You Letter

We ask that the physician use the template contained in this appendix to prepare a letter thanking the patient for enrolling in this trial. The template is intended as a guide and can be downloaded from the web site at http://www.ecog.org. As this is a personal letter, physicians may elect to further tailor the text to their situation.

This small gesture is a part of a broader program being undertaken by ECOG-ACRIN and the NCI to increase awareness of the importance of clinical trials and improve accrual and follow-through. We appreciate your help in this effort.

[PATIENT NAME] [DATE]

[PATIENT ADDRESS]

Dear Mr. / Mrs. / Ms. ___________,

Thank you for agreeing to take part in this important research study. Many questions remain unanswered in cancer. With the participation of people like you in clinical trials, we will improve treatment and quality of life for those with your type of cancer.

We believe you will receive high quality, complete care. I and my research staff will maintain very close contact with you. This will allow me to provide you with the best care while learning as much as possible to help you and other patients.

On behalf of ___________________ and ECOG-ACRIN, we thank you again and look forward to helping you.

Sincerely,

[PHYSICIAN NAME]
