# Supplementary material for: Academic Community Partnership in Acute Promyelocytic Leukemia and Early Mortality: The ECOG-ACRIN EA9131 Trial
Source: JAMA Oncol. 2025 Feb 27;11(4):400–7. doi: 10.1001/jamaoncol.2024.7033 (PMC11869096; doi:10.1001/jamaoncol.2024.7033)
Supplement: Supplement 2. — eTable. Resource Utilization Community Centers vs Lead Sites eFigure. Example Email Sent to Physicians [file jamaoncol-e247033-s002.pdf]

Supplemental Online Content

Jillella AP, Lee SJ, Altman JK, et al. Academic community partnership in acute promyelocytic leukemia and early mortality: the ECOG-ACRIN EA9131 trial. *JAMA Oncol.* Published online February 27, 2025. doi:10.1001/jamaoncol.2024.7033

eTable. Resource Utilization Community Centers vs Lead Sites

eFigure. Example Email Sent to Physicians

This supplemental material has been provided by the authors to give readers additional information about their work.

© 2025 Jillella AP et al. *JAMA Oncology*.

eTable. Resource Utilization Community Centers vs Lead Sites

	Community sites	Lead sites
Duration of stay (n=200)<br>Median days (range)	33 (12-86)	35 (2-55)
Blood product utilization<br>(n = 170 available),<br>median units (range)	N = 111 available<br>15 units (1-84)	N =59 available<br>18 units (1-62)
Admission to ICU (n=184)	3/59 (5.1%)	8/125 (6.4%

The hospital stay, total blood product utilization and ICU transfer rates were not statistically different between the lead and community sites.

© 2025 Jillella AP et al. *JAMA Oncology*.

eFigure. Example Email Sent to Physicians

On Jul 14, 2020, at 6:40 PM, XX* wrote:

35 year old with suspected high risk APL. No comorbid conditions and patient is not obese.

1. CBC, CMP, PT, PTT, fibrinogen at admission and twice a day after that.
2. D dimers q day.
3. Chest X-ray at admission
4. Echocardiogram
5. PICC line- NO central lines in chest or neck.
6. Allopurinol 300 q day
7. Antibiotic prophylaxis - levofloxacin 500 mg daily. micafungin 50 mg daily, Acyclovir 400 bid.
8. Keep fibrinogen above 150. Give 10 units of cryo if Fibrinogen is less than 150.
9. Keep platelets above 50 K.
10. Daily weights - **ON A BEDSIDE SCALE**. **Keep I/O matched - use diuretics for fluid retention or weight gain and do not let the patient gain weight. Would leave the fluids at KVO.**
11. **Dexamethasone 10 mg IV bid.**
12. ATRA 45 mg/m2 in divided doses.
13. Idarubicin 12 mg/m2 on days 1, 3 and 5
14. Can start ATO on Day 10 -- 0.15 mg/Kg

I am available 24/7. If you cannot reach me for any reason please call Dr. XX
Please email to this string so we can maintain a communication log that is a study requirement.
Regards and many thanks for your support of our study. XX

*APL Expert

© 2025 Jillella AP et al. *JAMA Oncology*.
