# Supplementary material for: Academic Community Partnership in Acute Promyelocytic Leukemia and Early Mortality: The ECOG-ACRIN EA9131 Trial
Source: JAMA Oncol. 2025 Feb 27;11(4):400–7. doi: 10.1001/jamaoncol.2024.7033 (PMC11869096; doi:10.1001/jamaoncol.2024.7033)
Supplement: Supplement 3. — Data Sharing Statement [file jamaoncol-e247033-s003.pdf]

Data Sharing Statement

Jillella. Academic-Community Partnership and Deaths in Promyelocytic Leukemia. *JAMA Oncol*. Published February 27, 2025. doi:10.1001/jamaoncol.2024.7033

Data
Additional Information: NCT03253848
Data available: Yes
Data types: Deidentified participant data
How to access data: Data available from ECOG databases
When available: With publication

Supporting Documents
Document types: None

Additional Information
Who can access the data: anyone requesting the data through the ECOG publications
Types of analyses: for any purpose
Mechanisms of data availability: After approval of proposal from the ECOG Leukemia group
